# [BO_2_]^−^ as a Synthon for the Generation of Boron‐Centered Carbamate and Carboxylate Isosteres

**DOI:** 10.1002/anie.202005674

**Published:** 2020-06-04

**Authors:** Anne‐Frédérique Pécharman, Michael S. Hill, Claire L. McMullin, Mary F. Mahon

**Affiliations:** ^1^ Department of Chemistry University of Bath Bath BA2 7AY UK

**Keywords:** boryl, density functional theory, dioxoborane, magnesium, main group chemistry

## Abstract

Oxoborane carbamate and carboxylate analogues result from the in situ trapping of [BO_2_]^−^ produced by elimination of 2,3‐dimethyl‐2‐butene from a pinacolatoboryl anion.

## Graphical abstract

**Playing the OBO**: Oxoborane carbamate and carboxylate analogues result from the in situ trapping of [BO_2_]^−^ produced by elimination of 2,3‐dimethyl‐2‐butene from a magnesium pinacolatoboryl anion.

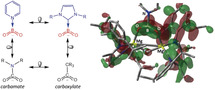

Organoboron oxides comprising three‐coordinate boron have been known since the mid‐1930s,^1^ and typically exist as cyclotrimeric anhydrides, [RBO]_3_, of the corresponding organoboronic acids.^2^ Similarly, naturally occurring inorganic metaborates such as NaBO_2_ comprise the trimeric [B_3_O_6_]^3−^ unit rather than discrete [BO_2_]^−^ anions.^3^ These observations are a thermodynamic consequence of the strong B−O bond (809 kJ mol^−1^) and the latent Lewis acidity of the boron center.^4^ A number of noteworthy recent advances in the chemistry of lower nuclearity oxoborane derivatives, however, have been achieved either through the incorporation of kinetically stabilizing substituents,^5^ for example in Aldridge's isolated oxoborane anion (**1**, Figure 1 a),^6^ or by saturation of the Lewis basic oxo and Lewis acidic boron units.^7^, ^8^ This latter approach is exemplified by Rivard and co‐workers’ isolation of [(IPr)(HO)B=OB(C_6_F_5_)_3_] (**2**, IPr=*N*,*N*′‐bis(2,6‐di‐isopropylphenylimidazol‐2‐ylidene, Figure 1 a) in which the stability of the HOB=O unit is maintained through the donor‐acceptor combination of an N‐heterocyclic carbene (NHC) and the potent Lewis acid, B(C_6_F_5_)_3_.^8^ Isoelectronic carbon‐for‐boron replacement identifies compound **2** as a neutral B(C_6_F_5_)_3_‐stabilized boron analogue of a carboxylic acid. Although further chemistry of **2** is yet to be described, recognition of this relationship prompts speculation that the conjugate bases of such species (**A**, Figure 1 b) may be exploited in a similar manner to carboxylate anions, which are among the most commonly applied narrow bite angle bidentate or bridging ligands in coordination, supramolecular, biomedical and bioinorganic chemistry.^9^ Compound **2** is also a progenitor to other classes of unprecedented boron‐centered anions with isoelectronic organic equivalents, for example, the carbamate analogue **B** (Figure 1 b).

**Figure 1:**
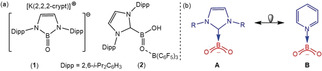
a) Compounds **1** and **2**; b) boron‐centered isosteres of carboxylate (**A**) and carbamate (**B**) anions.

Although viable quantities of **2** were achieved by Si−OH/B−Cl metathesis between Ph_3_SiOH and the chloroboroxane, [(IPr)ClB=OB(C_6_F_5_)_3_], the reaction required forcing conditions and generic syntheses of such species are currently unavailable.^8^ More attractive routes to anions such as **A** and **B**, therefore, would emulate those applied in the synthesis of their wholly organic analogues. The reaction of an organyl or amide anion with CO_2_, for example, provides a classical means to access carboxylate and carbamate anions, respectively. In contrast, similar routes to boron analogues are precluded by the unavailability of any suitable “boron dioxide” synthon. While the radical species, BO_2_, and the isoelectronic equivalent of CO_2_, [BO_2_]^−^, have been identified spectroscopically as short lived intermediates in borane flames or under matrix isolation conditions,^10^ and both have attracted significant theoretical attention as highly oxidising “hypohalogens”,^11^ these species neither exist as discrete entities nor have they been implicated in any productive synthesis. This lacuna is reminiscent of oxoborane (BO) chemistry prior to Braunschweig's report of *trans*‐[(Cy_3_P)_2_BrPt(BO)], which achieved the in situ generation of a terminal B≡O ligand through the reversible elimination of Me_3_SiBr from the B‐Br oxidative addition product of Br_2_BOSiMe_3_ and [Pt(PCy_3_)_2_].^4^, ^12^ In this contribution, we demonstrate the accessibility of [BO_2_]^−^ as a synthon through alkene elimination from isolable magnesium pinacolatoboryl species and its in situ trapping to provide boron‐centered analogues of carbamate and carboxylate anions.

The current work emerged from our studies of magnesium‐centered boryl nucleophiles and,^13^, ^14^, ^15^ specifically, their use in the construction of B−B′ bonds (Scheme 1).^16^, ^17^ We have previously reported that treatment of [(BDI)MgBu] (BDI=HC{C(Me)N‐2,6‐*i*‐Pr_2_C_6_H_3_}_2_] with bis(pinacolato)diboron (B_2_pin_2_) provides the diboranate derivative, compound **3** (Scheme 1). The [B(sp^2^)−B(sp^3^)] bond of compound **3** cleaves heterolytically when treated with bases such as 4‐dimethylaminopyridine (DMAP), providing compound **4** that comprises a terminal boryl anion without the direct use of a strong reductant (Scheme 1).^13^, ^14^, ^18^ The boron center of compound **4** displays nucleophilic character and reacts with carbon‐ and boron‐centered electrophiles to enable the construction of C−B and B−B′ bonds.^15^, ^16^, ^17^ Although the copper(I) derivative [(IPr)CuBpin] has recently been utilized in a similar manner,^19^ examples of unsymmetrical [B(sp^2^)−B(sp^2^)] diboranes were previously limited to compounds obtained by the desymmetrization of pre‐existing diborane(4) B−B bonds.^20^, ^21^ A notable case in point is pinB‐B(Mes)_2_ (**6**), synthesized by reaction of B_2_pin_2_ with mesityl magnesium bromide, which has been shown to effect the activation of a variety of small molecule substrates.^21^, ^22^, ^23^

**Scheme 1:**
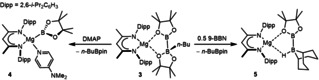
Synthesis of compounds **4** and **5**.^13^, ^16^

In an attempt to develop an alternative synthesis of compound **6**, therefore, we carried out the reaction of compound **4** with Mes_2_BF (Mes=2,4,6‐trimethylphenyl). The formation of compound **6** was identified in the resultant ^1^H NMR spectrum after five hours at 60 °C. This analysis, however, also revealed that the adduct complex, Mes_2_BF⋅DMAP (**7**), identified by its independent synthesis, accounted for ca. 50 % of the initially added fluoroborane. While minor quantities (<10 %) of the anticipated dimeric magnesium fluoride by‐product, [(BDI)MgF]_2_ (**8**) were observed,^24^ the majority of the magnesium β‐diketiminate ^1^H NMR resonances could be ascribed to a single new BDI‐containing product (**9**). Compound **9** was isolated in 48 % yield by fractional crystallization and identified by single crystal X‐ray diffraction as a dinuclear magnesium μ_2_‐fluoride in which charge balance is maintained by a bridging [4‐Me_2_NC_5_H_4_NBO_2_]^−^ anion (Scheme 2). Insight into the fate of the [pinB]^−^ anion of **4** and the origin of the [4‐Me_2_NC_5_H_4_NBO_2_]^−^ ligand was provided by a further experiment performed in C_6_D_6_. Although this reaction proceeded identically, vacuum transfer of the volatile products delivered a single component, most clearly manifested as a singlet resonance centered at 1.62 ppm in its ^1^H NMR spectrum, that was readily identified as 2,3‐dimethyl‐2‐butene.

**Scheme 2:**
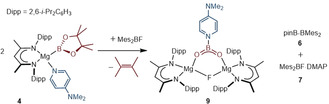
Synthesis of compound **9**.

The structure of **9** comprises two effectively identical dinuclear complexes (Figure 2, only the Mg1/Mg2‐containing molecule is discussed) in which the magnesium centers are connected by a single μ_2_‐bridging fluoride and an unprecedented boron‐centered [4‐Me_2_NC_5_H_4_NBO_2_]^−^ monoanion. The Mg−O bond lengths in **9** [1.918(2); 1.904(2) Å] are somewhat shorter than those observed in the only similarly dinuclear magnesium carbamates [ca. 1.95–2.0 Å], albeit the group 2 centers of these previously reported compounds are five‐ rather than four‐coordinate.^25^ Like the {CBO_2_} unit of compound **2**, N5, B1, O1 and O2 in **9** are coplanar and this plane subtends an angle of only 5.31° with the mean plane defined by the DMAP ligand. Despite this near coplanarity, the B1−N5 distance [1.589(4) Å] is elongated in comparison to typical covalent B−N bonds (e.g. the borylamidinate, pinB‐N(*i*‐Pr)HC=N*i*‐Pr, 1.42731(6) Å).^26^ In contrast, the identical B1−O1 [1.320(4) Å] and B1−O2 [1.324(4) Å] distances are comparable to the shorter of the B−O bonds [1.311(3) Å] of **2**.^8^ Natural bond orbital (NBO) analysis of compound **9** also afforded Wiberg bond indices for the B−O bonds (1.1365, 1.1390) that are closely comparable to that reported for the shorter B−O linkage in **2** (1.123).^8^ The values are indicative of multiple bond character, such that the planarity of the dioxoborane unit is a consequence of pronounced B(2p)−O(2p) π–π overlap across the {O‐B‐O} unit (Figure 2 b). These observations support the legitimacy of the simple valence bond depiction (structure **B** in Figure 1 b) of this anion as a boron‐centered carbamate analogue.

**Figure 2:**
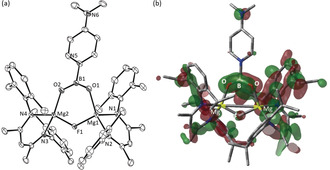
a) ORTEP of the Mg1/Mg2‐containing molecule of compound **9** (30 % probability ellipsoids). Hydrogen atoms, isopropyl methyl groups and occluded solvent are removed for clarity. Selected bond lengths [Å] and angles [°]: B1–O1 1.320(4), B1–O2 1.324(4), B1–N5 1.589(4), Mg1–O1 1.918(2), Mg2–O2 1.904(2), F1–Mg1 1.8953(18), F1–Mg2 1.8865(19); O1‐B1‐O2 134.1(3), O1‐B1‐N5 113.7(3), O2‐B1‐N5 112.2(3): b) Calculated Natural Bond Orbital (NBO) surface for HOMO−19 of compound **9**.^28^

Although the complexity of the reaction precluded more quantitative analysis, re‐examination of the aliphatic region of the ^1^H NMR spectra recorded at one hour intervals revealed the emergence of a further BDI‐magnesium species (**10**) [δ(^1^H) 4.97 ppm], which, although comprising ca. 20 % of the total BDI signals after three hours, diminished significantly in intensity during the latter stages of the reaction. Two signals at almost identical frequencies in the ^19^F{^1^H} NMR spectra (δ−113.77 and −113.83 ppm) displayed an analogous increase and decrease in relative intensity during the same time period and are also, therefore, attributed to compound **10**. Significantly, these latter resonances appeared in a strict 1:4 ratio of intensities throughout the reaction (Figure S5) and are, thus, assigned to natural abundance boron‐fluorine bonded ^10^B and ^11^B isotopomers of [(BDI)Mg{pinB‐BF(Mes_2_)}] (**10**).^27^ Although a dimesitylfluorodiboranate analogue of compound **5** (Scheme 1), which proved stable to boron‐to‐magnesium hydride elimination,^17^ compound **10** evidently degrades via boron‐to‐magnesium fluoride transfer and elimination of Yamashita's diborane (**6**).^21^

These observations lead us to suggest that the formation of compound **9** is a consequence of two competitive pathways, the thermodynamic viability of which have been confirmed by density functional theory (DFT) calculations. Scheme 3 summarizes the results of this analysis (see also Tables S2 and S3 in the SI).

**Scheme 3:**
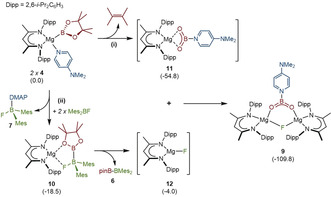
DFT computed pathway to compound **9**. Free energies (kcal mol^−1^, relative to **4**) of computed structures are shown in parenthesis.

The route identified as pathway (i) requires the elimination of 2,3‐dimethyl‐2‐butene and the in situ generation of a [BO_2_]^−^ equivalent. While [BO_2_]^−^ is not viable as a persistent species, its immediate trapping by a molecule of DMAP provides a cogent rationale for the generation of the [4‐Me_2_NC_5_H_4_NBO_2_]^−^ anion. Although the intermediacy of a monomeric species, **11**, is questionable, **9** may be considered to result from its combination with the putative three‐coordinate magnesium fluoride, compound **12**. We suggest, however, that the concurrent accumulation of minor quantities of the dimeric fluoride, [(BDI)MgF]_2_ (**8**), provides circumstantial evidence for the generation of **12** as a common intermediate.

The credibility of pathway (i) relies on the instability of the {Mg‐Bpin} unit toward alkene extrusion under the applied reaction conditions. Yamashita and co‐workers have reported that treatment of pinB‐B(Mes)_2_ (**6**) with 2,6‐dimethylphenyl isonitrile results in pinB ring contraction to provide a spirocyclic compound comprising a 4‐membered cyclic {BOC_2_} 1,2‐oxaboretane structure.^23^ In this earlier case, however, C−O bond cleavage was deduced to proceed via a carbocationic mechanism. The alkene elimination process identified in the formation of compound **9**, therefore, appears to be a unique observation that could carry important implications for Bpin‐related chemistry in general.

Calculations by Schleyer and co‐workers as long ago as 1995 highlighted that the stability of singlet model boryl derivatives, X_2_B−Li, is predicated on not only electronegative X substitution (e.g. F, O, N) but also the direct interaction of boron with the more electropositive lithium.^29^ These theoretical deductions were foreshadowed by Corey's even earlier demonstration that desulfurization of a cyclic pinacol‐derived thionocarbonate derivative (Scheme 4 a) results in elimination of 2,3‐dimethyl‐2‐butene due to the relative instability of the resultant carbene toward olefin and carbon dioxide formation.^30^ DFT calculations indicated that analogous transformation of the isoelectronic [pinB]^−^ anion to 2,3‐dimethyl‐2‐butene and [BO_2_]^−^ is significantly exergonic (Δ*G_f_*=−79.5 kcal mol^−1^, Scheme 4 b).

**Scheme 4:**
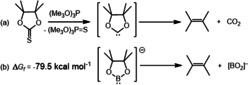
a) Corey's carbene decarboxylation strategy for the synthesis of 2,3‐dimethyl‐2‐butene;^30^ b) analogous elimination of 2,3‐dimethyl‐2‐butene from the [pinB]^−^ anion.

The thermodynamic viability of this process prompted us to attempt similar boryl decomposition to provide a boron‐centered carboxylate analogue akin to the NHC‐based anion **A** (Figure 1). The magnesium boryl [(BDI)Mg(Bpin)(*i*‐Pr‐NHC)] (**13**, *i*‐Pr‐NHC=1,3‐di‐isopropyl‐4,5‐dimethylimidazol‐2‐ylidene)^31^ was, therefore, prepared by an equimolar reaction of compound **3** and the N‐heterocyclic carbene (Scheme 5). Characterization by single‐crystal X‐ray diffraction analysis (Figure 3 a) revealed that the resultant Mg−B bond of compound **13** [2.3192(19) Å] is closely comparable to that observed in compound **4** [2.324(2) Å] indicating that incorporation of *i*‐Pr‐NHC results in only very limited perturbation to the electronic character of the {Bpin} ligand.

**Figure 3:**
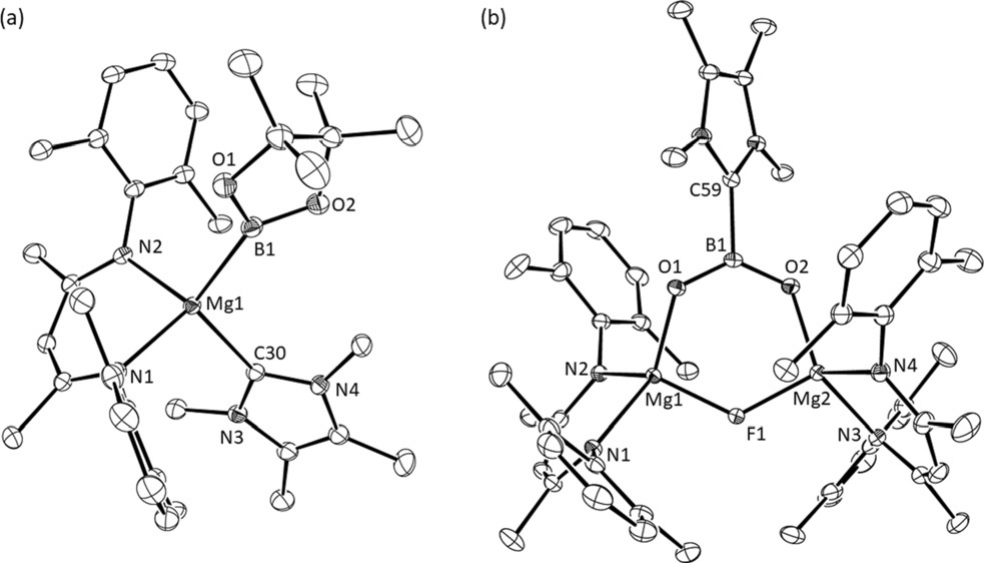
ORTEPs of (a) compound **13** and (b) compound **15** (30 % probability ellipsoids) Hydrogen atoms and isopropyl methyl groups are removed for clarity. Selected bond lengths [Å] and angles [°]: (**13**) Mg1–C30 2.2929(15), Mg1–B1 2.3192(19); N1‐Mg1‐C30 102.52(6), N1‐Mg1‐B1 124.92(7), N2‐Mg1‐N1 90.59(6), N2‐Mg1‐C30 113.71(6), N2‐Mg1‐B1 108.07(6), C30‐Mg1‐B1 114.86(7); (**15**) Mg1–F1 1.8926(8), Mg1–O1 1.9131(9), Mg2–F1 1.8959(8), Mg2–O2, 1.9170(9), O1–B1 1.3336(17), O2–B1 1.3324(17), C59–B1 1.6596(17); Mg1‐F1‐Mg2 125.84(4), B1‐O1‐Mg1 125.17(8), B1‐O2‐Mg2 125.07(8), O1‐B1‐C59 114.93(11), O2‐B1‐O1 130.54(11), O2‐B1‐C59 114.53(11).

**Scheme 5:**
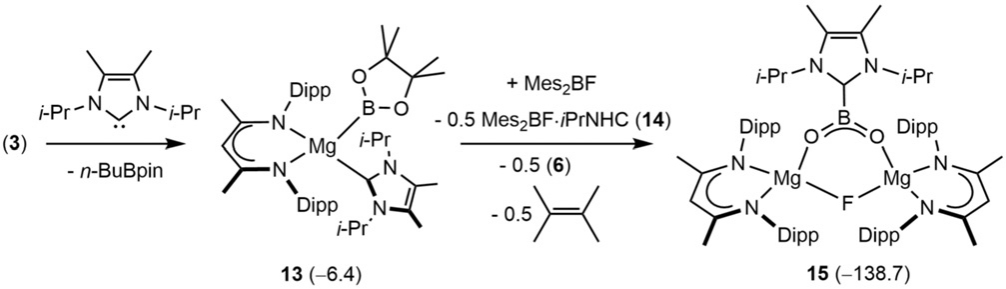
Synthesis of compounds **13** and **15**. Free energies (relative to **3** at 0.1 kcal mol^−1^) for DFT computed structures are shown in parenthesis.

A reaction of compound **13** and Mes_2_BF provided broadly analogous observations to those resulting from the reaction with compound **4**, albeit the transformation was significantly more facile and complete after only one hour at room temperature. Approximately 50 % of the Mes_2_BF was converted to the adduct species, Mes_2_BF⋅(*i*‐Pr‐NHC) (**14**), which was identified through its independent synthesis and clearly characterized in the resultant ^1^H NMR spectrum by the emergence of two deshielded (1 H) multiplet resonances at *δ* 5.45 and 5.01 ppm and a series of twelve differentiated (3 H) methyl resonances. The formation of 2,3‐dimethyl‐2‐butene was also clearly identifiable as a 12 H signal at *δ* 1.62 ppm, alongside the simultaneous production of an approximately equimolar quantity of pinB‐BMes_2_ [**6**, *δ* 2.35 (s, 12 H), 2.15 (s, 6 H), 1.07 (s, 12 H) ppm]. Most significantly, these transformations were accompanied by the emergence of a series of broadened BDI‐ligand resonances attributed to the formation of a single new compound, identified by a subsequent X‐ray diffraction analysis as the dinuclear β‐diketiminato magnesium complex (**15**). The overall stoichiometry of the reaction, therefore, may be rationalized as depicted in Scheme 5.

Like **9**, compound **15** (Figure 3 b) comprises a dinuclear [(BDI)Mg‐μ_2_‐F‐Mg(BDI)] unit. In the case of **15**, however, the coordination environment of each Mg center is completed by a dioxoboron monoanion in which the final bond to the trigonal boron is provided by an equivalent of the *i*‐Pr‐NHC donor. This unit as a whole, therefore, may be classified as a conjugate base of a boron‐centered carboxylic acid analogue (cf. **A**, Figure 1 b). The Mg−O bonds of **15** [1.9131(9), 1.9170(9) Å] are effectively identical to the shorter of the comparable measurements in the similarly four‐coordinate carboxylate derivatives, [(BDI)Mg(μ‐O_2_CR)]_2_ [R=Me, 1.918(2), 1.941(2); R=Ph, 1.918(2), 1.958(2) Å].^32^ As in the case of compound **9**, the B−O bond distances are similar [O1−B1 1.3336(17), O2−B1 1.3324(17) Å], while the NHC‐to‐boron interaction [C59−B1 1.6596(17) Å] is only marginally elongated in comparison to the C−B distance [C−B 1.636(3) Å] reported for the formally charge neutral {CBO_2_} unit of compound **2**.^8^

In conclusion, we report the synthesis of unique dioxoborane analogues of the ubiquitous carbamate and carboxylate anions. Both moieties result from the apparent in situ trapping of the highly reactive [BO_2_]^−^ anion by a neutral *N*‐ or *C*‐centered base, subsequent to the kinetically facile and thermodynamically viable elimination of 2,3‐dimethyl‐2‐butene from well‐defined magnesium‐coordinated boryl anions. We are continuing to study this reactivity and to elaborate the more general coordination chemistry of these unprecedented anions.

Deposition Numbers 1997822, 1997823, 1997824, and 1997825 contain the supplementary crystallographic data for this paper. These data are provided free of charge by the joint Cambridge Crystallographic Data Centre and Fachinformationszentrum Karlsruhe Access Structures service www.ccdc.cam.ac.uk/structures.

## Conflict of interest

The authors declare no conflict of interest.

## Supporting information

As a service to our authors and readers, this journal provides supporting information supplied by the authors. Such materials are peer reviewed and may be re‐organized for online delivery, but are not copy‐edited or typeset. Technical support issues arising from supporting information (other than missing files) should be addressed to the authors.

Supplementary
