# Supplementary material for: [BO2]− as a Synthon for the Generation of Boron‐Centered Carbamate and Carboxylate Isosteres
Source: Angew Chem Int Ed Engl. 2020 Jun 4;59(32):13628–32. doi: 10.1002/anie.202005674 (PMC7496551; doi:10.1002/anie.202005674)
Supplement: Supplementary file 1 — Supplementary [file ANIE-59-13628-s001.pdf]

Angewandte Chemie
Eine Zeitschrift der Gesellschaft Deutscher Chemiker

Supporting Information

$[BO_2]^-$ as a Synthon for the Generation of Boron-Centered Carbamate and Carboxylate Isosteres

Anne-Frédérique Pécharman, Michael S. Hill, Claire L. McMullin,* and Mary F. Mahon*

anie_202005674_sm_miscellaneous_information.pdf

Experimental Procedures

General considerations and starting materials

All manipulations were carried out using standard Schlenk line and glovebox techniques under an inert atmosphere of argon. NMR experiments were conducted in J Young tap NMR tubes made up and sealed in a Glovebox. NMR spectra were recorded on a Bruker AV400 spectrometer or an Agilent ProPulse spectrometer operating at 500 MHz (^{1}H), 126 MHz (^{13}C), 160.4 MHz (^{11}B), 470.4 (^{19}F). The spectra were referenced relative to residual solvent resonances or an external $BF_3.OEt_2$ standard (^{11}B). GCMS data were acquired on an Agilent GC-MSD equipped with thermal desorption for volatile and semi-volatile organic compounds. Elemental analysis was carried out by Elemental Microanalysis, Okehampton, UK. Solvents (toluene, hexane) were dried by passage through a commercially available (Innovative Technologies) solvent purification system, under nitrogen and stored in ampoules over molecular sieves. d_8-toluene was purchased from Fluorochem Ltd. and Sigma-Aldrich Ltd. and dried over molten potassium before distilling under argon and storing over molecular sieves. Bis(pinacolato)diboron and dimesitylboron fluoride were purchased from Sigma Aldridge Ltd and sublimed before use. [(BDI)MgBu], [(BDI)Mg{pinB}(DMAP)] (**4**) and 1,3-di-isopropyl-4,5-dimethylimidazol-2-ylidene were synthesized by literature procedures.[1-3]

Synthesis of compound 9, [{(BDI)Mg}$_2$(F)($O_2BNC_5H_4$-4-NMe_2)]

Toluene-d_8 (0.5 mL) was added to a mixture of compound **4** (40 mg, 0.058 mmol) and Mes_2BF (15.44 mg, 0.058 mmol) in a J Youngs NMR tube. The resultant solution was heated at 60 °C for five hours. Volatiles were removed under reduced pressure and the solid residue was crystallized from a hexane solution to yield compound **9** as colorless crystals (15 mg, 48%). ^{1}H NMR (500 MHz, toluene-d_8, 298 K) δ 7.72 (br s, 2H DMAP), 7.12 (ar C*H*), 7.04 (ar C*H*), 7.00 (ar C*H*), 5.44 (br s, 2H DMAP), 4.90 (s, 2H, NC(CH_3)C*H*), 3.54 (hept, 4H, J_{HH} = 6.8Hz, C*H*(CH_3)$_2$), 1.87 (s, 6H, N(CH_3)$_2$, DMAP), 1.72 (s, 12H, NC(CH_3)CH), 1.49 (br s, 6H, CH_3), 1.35 (d, 12H, CH_3) ppm (some resonances were unobservable at room temperature but resolved into additional broad signals when the solution was cooled to –50 °C, see Figure S10). ^{19}F{^{1}H} NMR (470 MHz, toluene-d_8, 298 K) δ –181.3 ppm. ^{11}B NMR no observable signal. ^{13}C{^{1}H} NMR (126 MHz, toluene-d_8, 298 K) δ 167.6 (N$\underline{C}$(CH_3)CH), 155.5, 143.9, 142.00 ($\underline{C}$H ar DMAP), 124.3 ($\underline{C}$H ar), 123.1 ($\underline{C}$H ar), 104.0 ($\underline{C}$H ar DMAP), 94.2 (NC(CH_3)$\underline{C}$H), 37.7 ($\underline{C}H_3$ DMAP), 28.0 ($\underline{C}H_3$), 27.7 ($\underline{C}H_3$), 24.9 ($\underline{C}H_3$), 24.1 ($\underline{C}H_3$), 24.0 (NC($\underline{C}H_3$)CH) ppm. Elemental analysis: Found C, 73.37; H, 8.61, N, 7.70%. $C_{65}H_{92}BFMg_2N_6O_2$ requires: C, 73.11; H, 8.68; N, 7.87%.

Synthesis of compound 13, [(BDI)Mg(Bpin)(C{N(*i*-Pr)C(Me)}$_2$)]

Toluene-d_8 (0.5 mL) was added to a mixture of [(BDI)MgBu] (200 mg, 0.40 mmol) and bis(pinacolato)diboron (107 mg, 0.41 mmol) to form **3**. After two hours, an equimolar quantity of 1,3-di-isopropyl-4,5-dimethylimidazol-2-ylidene (72.2 mg, 0.40 mmol) was added. After 15 minutes at

room temperature, volatiles were removed under reduced pressure and the resultant solid was crystallized from hexane solution at –35 ˚C to yield compound **13** in (155 mg, 52%). Colorless crystals suitable for X-ray diffraction studies were obtained from a saturated toluene/hexane solution at room temperature. ^{1}H NMR (500 MHz, toluene-d_8, 298 K) δ 7.27 (m, 2H, CH ar), 7.12-7.00 (m, 4H, CH ar), 6.34 (hept, 1H, J_{HH} = 6.7Hz, NCH(CH_3)$_2$), 5.21(hept, 1H, J_{HH} = 6.7Hz, NCH(CH_3)$_2$), 4.89 (s, 1H, NC(CH_3)CH), 3.76 (hept, 2H, J_{HH} = 6.7Hz, CH(CH_3)$_2$), 3.13 (hept, 2H, J_{HH} = 6.8Hz, CH(CH_3)$_2$), 1.85 (s, 3H, CH_3C(N)CCH_3), 1.79 (s, 3H, CH_3C(N)CCH_3), 1.75 (s, 6H, NC(CH_3)CH), 1.69 (d, 6H, J_{HH} = 6.7 Hz, CH(CH_3)$_2$), 1.39 (d, 6H, J_{HH} = 6.7 Hz, NCH(CH_3)$_2$), 1.36 (d, 6H, J_{HH} = 6.7 Hz, CH(CH_3)$_2$), 1.26 (d, 6H, J_{HH} = 6.7 Hz, NCH(CH_3)$_2$), 1.18 (d, 6H, J_{HH} = 6.7 Hz, CH(CH_3)$_2$), 0.93 (s, 12H, B(OC(CH_3)$_2$)$_2$), 0.83(d, 6H, J_{HH} = 6.7 Hz, CH(CH_3)$_2$) ppm. ^{11}B NMR no observable signal. ^{13}C{^{1}H} NMR (126 MHz, toluene-d_8, 298 K) δ 186.4($N_2\underline{C}$, NHC), 166.7 (N$\underline{C}$(CH_3)CH), 146.7, 143.1, 141.7, 125.2 ($\underline{C}$H ar), 124.1 ($\underline{C}$H ar), 124.1 ($\underline{C}$H ar), 123.6 ($\underline{C}$H ar), 122.7 ($\underline{C}$H ar), 93.6 (NC(CH_3)$\underline{C}$H), 78.6 ((B(O$\underline{C}$(CH_3)$_2$)$_2$), 53.0 (N$\underline{C}$H(CH_3)$_2$), 51.2 (N$\underline{C}$H(CH_3)$_2$), 28.5 ($\underline{C}$H(CH_3)$_2$), 26.8 ($\underline{C}$H(CH_3)$_2$), 25.6 ((B(OC($\underline{C}H_3$)$_2$)$_2$), 25.0 (CH($\underline{C}H_3$)$_2$), 24.8 (CH($\underline{C}H_3$)$_2$), 24.6 (CH($\underline{C}H_3$)$_2$), 24.4 (CH($\underline{C}H_3$)$_2$), 24.1 (NC($\underline{C}H_3$)CH), 22.5 (CH($\underline{C}H_3$)$_2$), 22.0 (CH($\underline{C}H_3$)$_2$), 9.8 (NCH($\underline{C}H_3$)$_2$), 9.8 (NCH($\underline{C}H_3$)$_2$) ppm. Despite multiple attempts, no microanalysis could be obtained for this highly air- and moisture-sensitive compound.

Synthesis of compound 15, [{(BDI)Mg}$_2$(F)(O_2BC{N(*i*-Pr)C(Me)}$_2$)]

Toluene-d_8 (0.5 mL) was added to a mixture of compound **13** (30 mg, 0,04 mmol) and Mes_2BF (10.7 mg, 0.04 mmol) before volatiles were removed under reduced pressure after one hour at room temperature. The resultant colorless solid was dissolved in hexane and crystallized at –35 °C to provide compound **15** as colorless crystals (12 mg, 53%). ^{1}H NMR (500 MHz, toluene-d_8, 298 K) δ 7.31-7.19 (m, 6H, CH ar), 6.95-6.84 (m, 6H, CH ar), 5.26 (br s, 2H, NCH(CH_3)$_2$), 4.83 (s, 1H, NC(CH_3)CH), 3.84 (br s, 2H, CH(CH_3)$_2$), 3.42 (br s, 4H, CH(CH_3)$_2$), 3.14 (br s, 2H, CH(CH_3)$_2$), 1.64-1.50 (m, 12H, CH_3), 1.44-1.31 (m, 18H, CH_3), 1.24-1.16 (m, 36H, CH_3), 0.59 (br s, 6H, CH_3), 0.24 (br s, 6H, CH_3) ppm. ^{19}F{^{1}H} NMR (377 MHz, toluene-d_8, 298 K) δ –176.4. ^{13}C{^{1}H} NMR (126 MHz, toluene) δ 167.6 ($N_2\underline{C}$, NHC), 167.5 (N$\underline{C}$(CH_3)CH), 147.8, 147.7, 144.3, 143.5, 142.7, 142.3, 142.2, 142.0, 125.4 ($\underline{C}$H ar), 125.2 ($\underline{C}$H ar), 123.7 ($\underline{C}$H ar), 123.6 ($\underline{C}$H ar), 123.1 ($\underline{C}$H ar), 122.90 ($\underline{C}$H ar), 122.7 ($\underline{C}$H ar), 122.1 ($\underline{C}$H ar), 110.0, 94.6 (NC(CH_3)$\underline{C}$H), 49.5 (N$\underline{C}$H(CH_3)$_2$), 28.1 ($\underline{C}$H(CH_3)$_2$), 27.4 ($\underline{C}$H(CH_3)$_2$), 27.2 ($\underline{C}$H(CH_3)$_2$), 25.8 ($\underline{C}H_3$), 25.6 ($\underline{C}H_3$), 25.2 ($\underline{C}H_3$), 25.0 ($\underline{C}H_3$), 24.9 ($\underline{C}H_3$), 24.6 ($\underline{C}H_3$), 24.4 ($\underline{C}H_3$), 24.3 ($\underline{C}H_3$), 23.8 ($\underline{C}H_3$), 23.3 ($\underline{C}H_3$), 23.2 ($\underline{C}H_3$), 23.0 ($\underline{C}H_3$), 22.7 ($\underline{C}H_3$), 22.1 ($\underline{C}H_3$), 21.3 ($\underline{C}H_3$), 13.9 ($\underline{C}H_3$), 9.5 ($\underline{C}H_3$) ppm. ^{11}B NMR provided no observable signals. Elemental analysis: Found C, 73.38; H, 8.95, N, 7.35%. $C_{69}H_{103}BFMg_2N_6O_2$ requires: C, 73.53; H, 9.21; N, 7.46%.

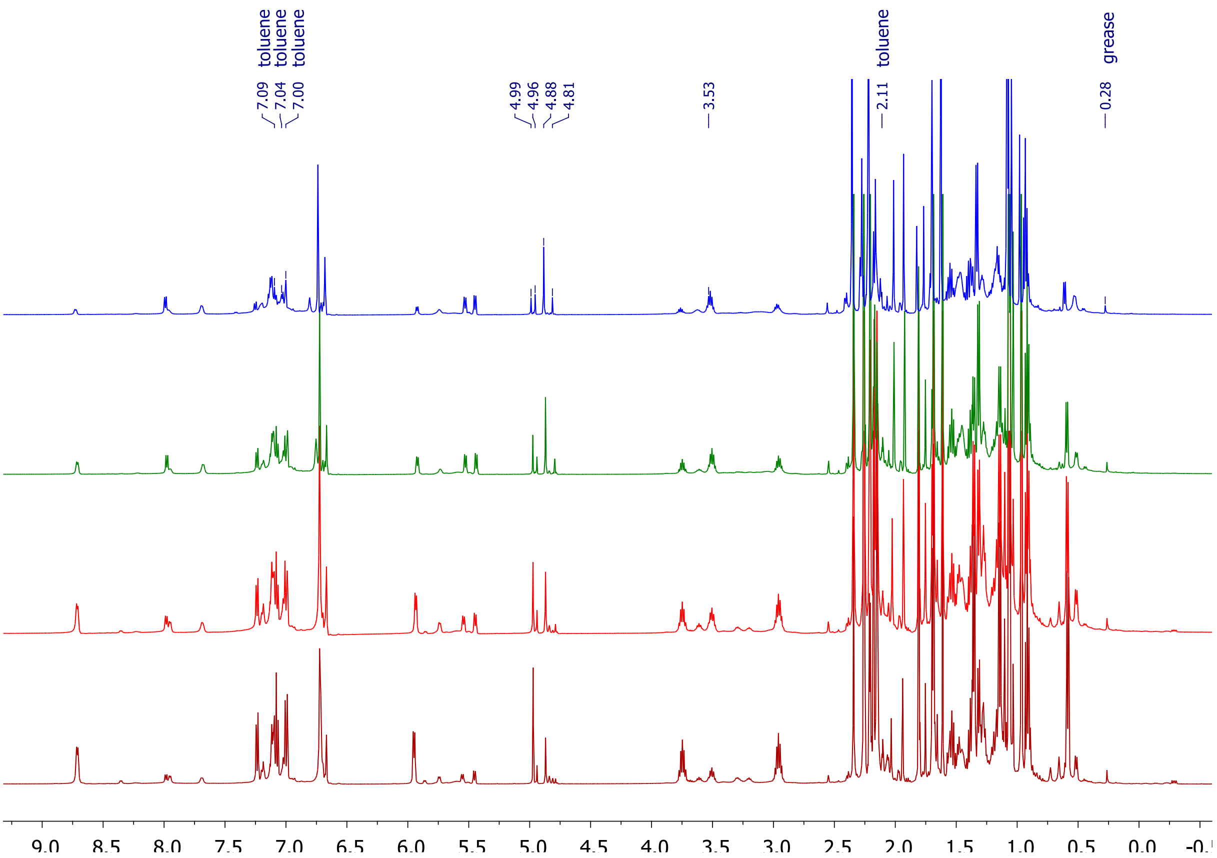

Figure S1: Stacked ^{1}H NMR spectra (toluene-d_8, 500 MHz, 298 K) of the reaction of compound **4** and Mes_2BF at 60 °C after 1 hour (brown), 2 hours (red), 3 hours (green) and 5 hours (blue) and highlighting the respective BDI singlet and *i*-Pr multiplet methine signals of compound **9** at δ 4.88 and 3.53 ppm.

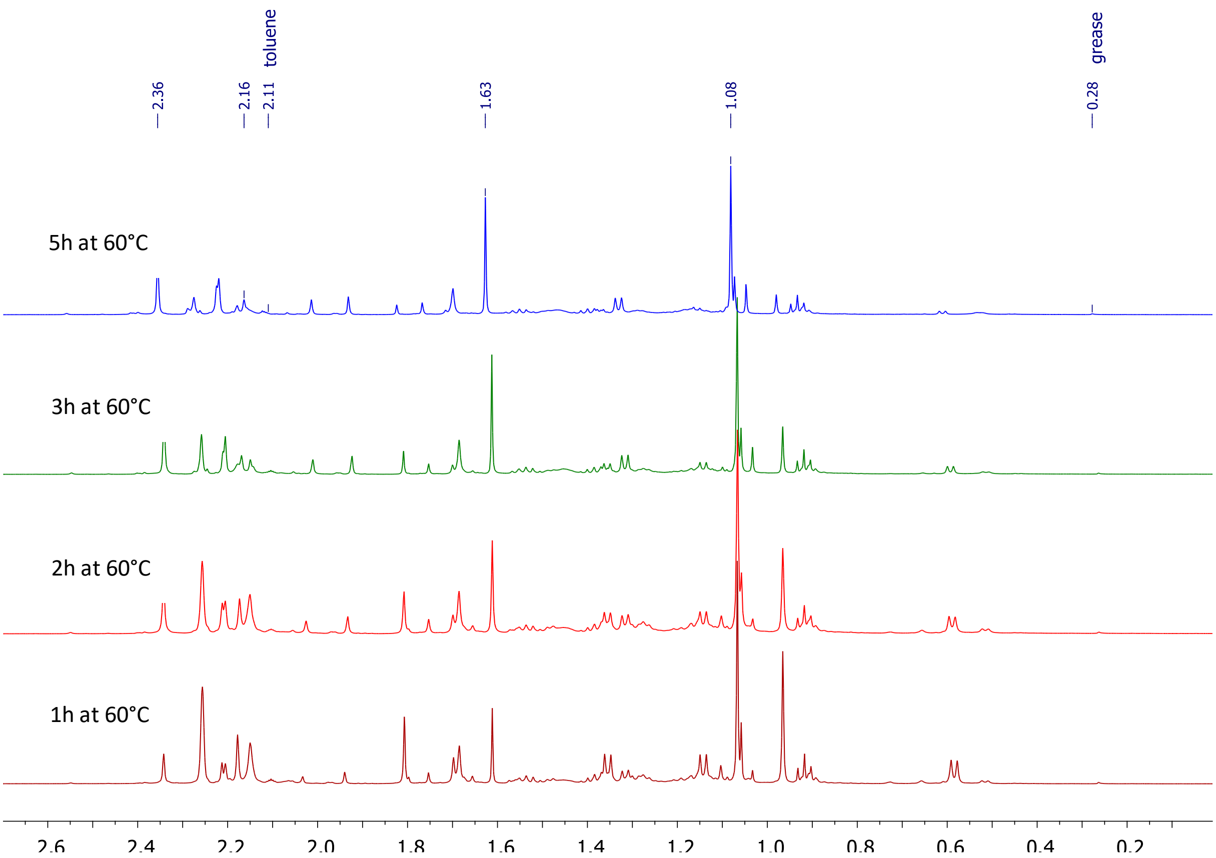

Figure S2: Stacked ^{1}H NMR spectra (toluene-d_8, 500 MHz, 298 K) of the aliphatic region of the reaction of compound **4** and Mes_2BF at 60 °C after 1 hour (burgundy) (b) 2 hours, (c) 3 hours, (d) 5 hours and highlighting the emergence of pinB-$BMes_2$ (**6**) at δ 2.36, 2.16 and 1.08 ppm and 2,3-dimethyl-2-butene at δ 1.63 ppm.

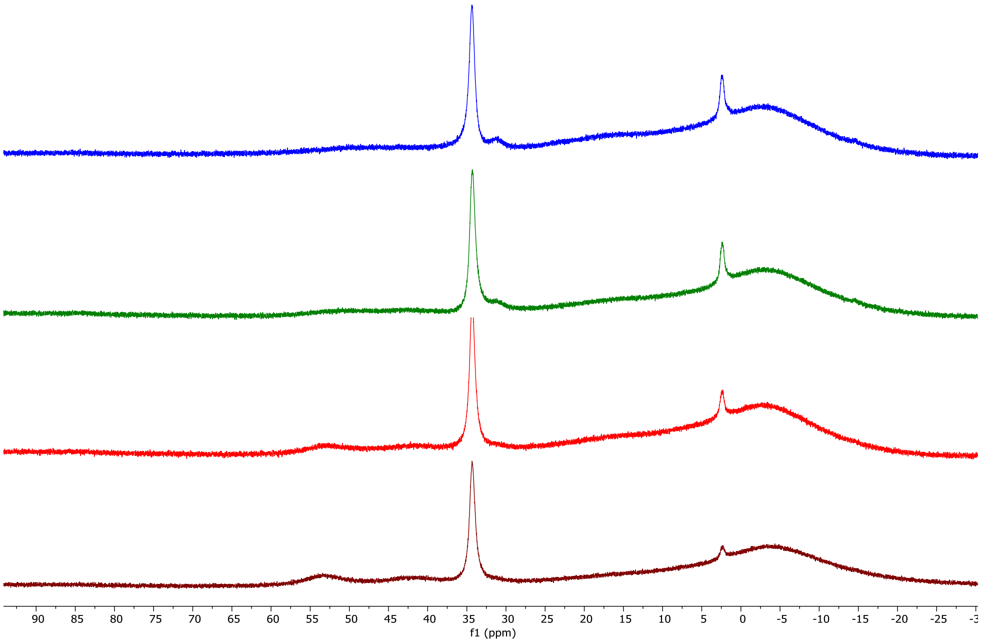

Figure S3: Stacked ^{11}B NMR spectra (toluene-d_8, 160.4 MHz, 298 K) of the reaction of compound **4** and Me_2BF at 60 °C after 1 hour (brown), 2 hours (red), 3 hours (green) and 5 hours (blue) and highlighting the disappearance of Mes_2BF (broad signal at *ca.* δ 53 ppm and the emergence of pin<u>B</u>-$BMes_2$ (**6**) at δ 34.2 and of Mes_2BF·DMAP (**7**) at δ 2.3 ppm.

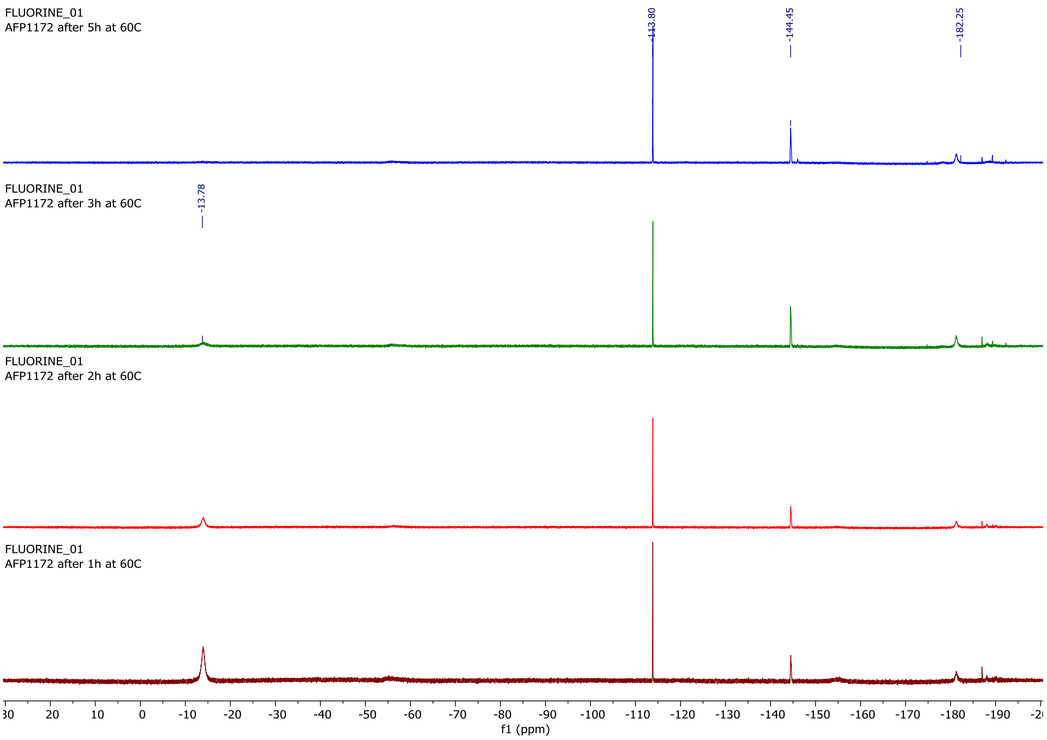

Figure S4: Stacked $^{19}F\{^1H\}$ NMR spectra (toluene-d_8, 470 MHz, 298 K) of the reaction between compound **4** and Me_2BF at 60 °C after 1 hour (brown), 2 hours (red), 3 hours (green) and 5 hours (blue) and highlighting the disappearance of Mes_2BF (broad signal at *ca.* δ –13 ppm and the emergence of **10** (δ –113 ppm), Mes_2BF·DMAP (**7**, δ –144 ppm) and compound **9** (broad signal at δ –182 ppm).

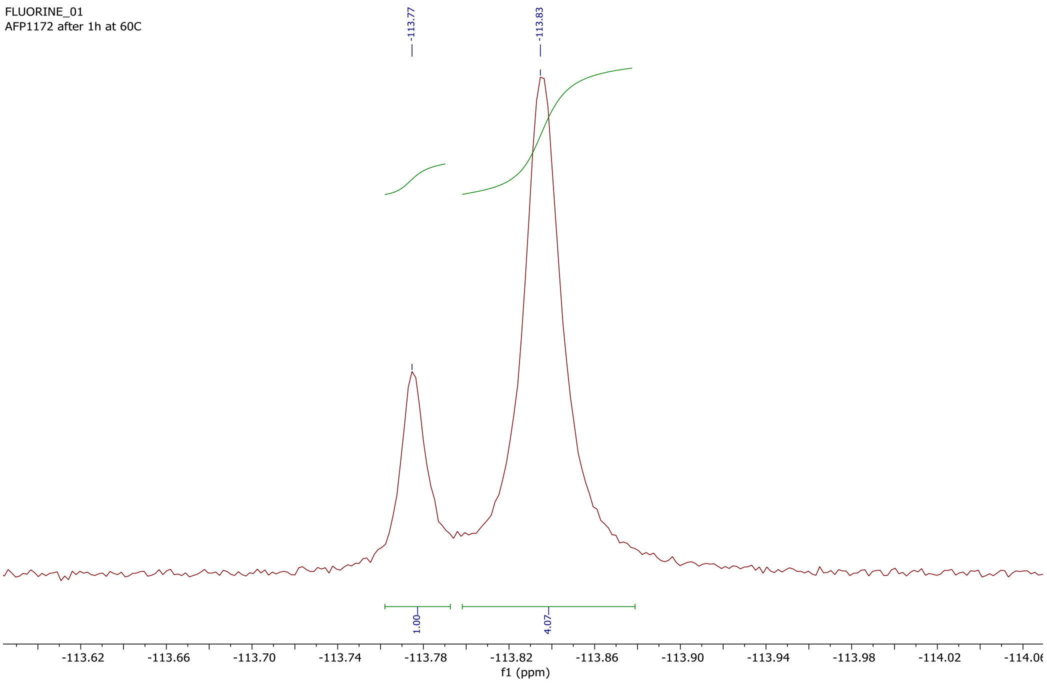

Figure S5: Expansion of the signal at *ca.* –113 ppm in the $^{19}F\{^1H\}$ NMR spectra (toluene-d_8, 470.4 MHz,298 °C) of the reaction of compound **4** and Me_2BF after 1 hour at 60 °C and assigned as the ^{10}B (δ –113.77 ppm) and ^{11}B (δ –113.83) isotopomers of compound **10**, [(BDI)Mg{pinB-B(F)Mes_2}].

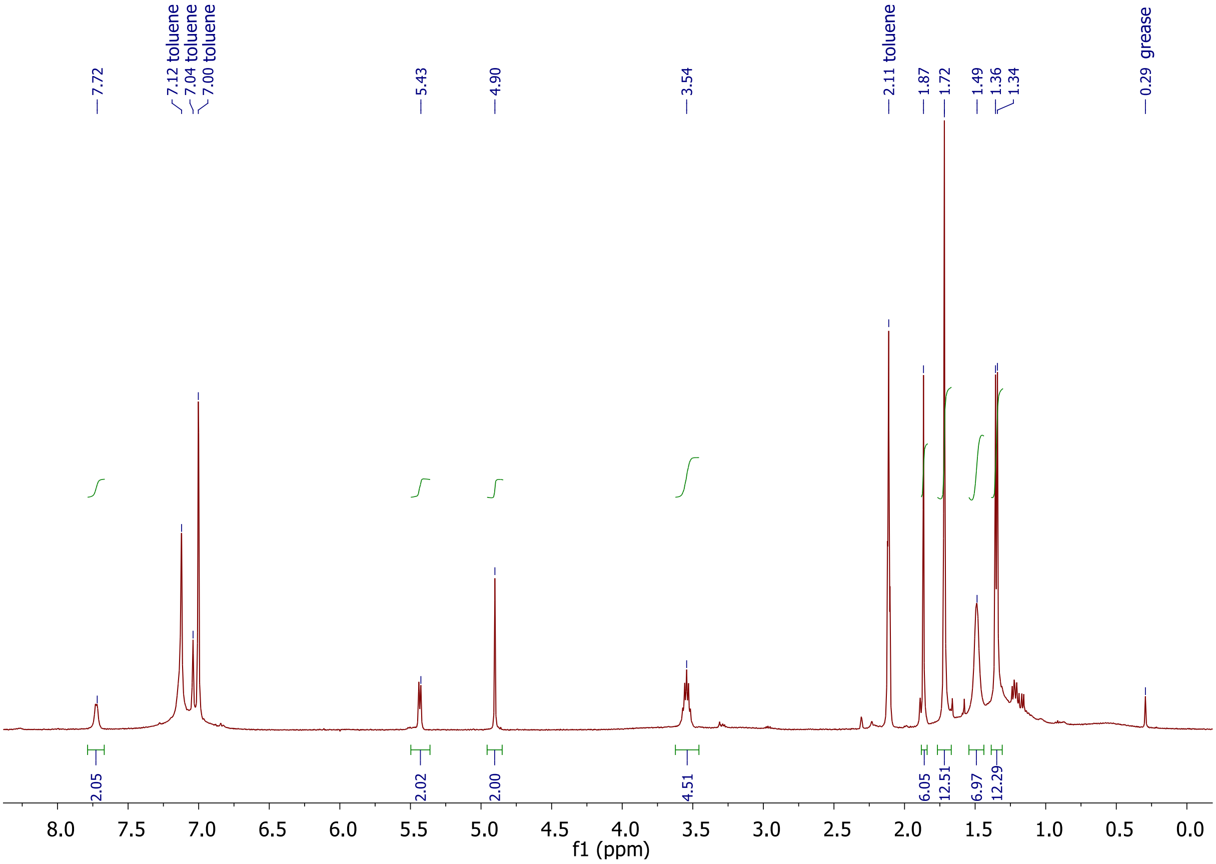

Figure S6: ^{1}H NMR spectrum (toluene-d_8, 500MHz, 298 K) of compound **9**.

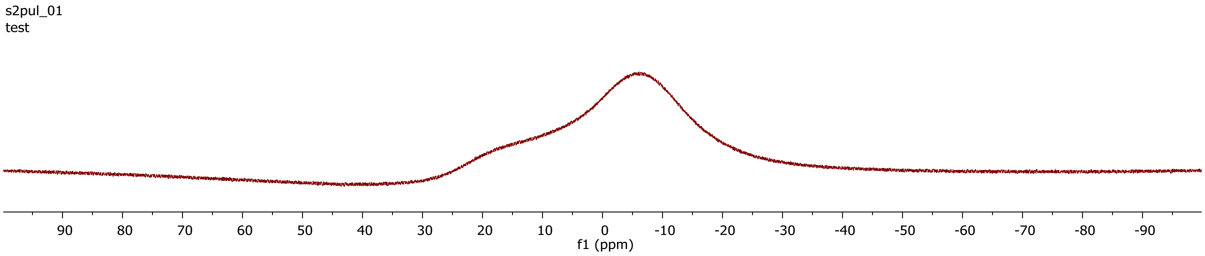

Figure S7: ^{11}B{^{1}H} NMR spectrum (toluene-d_8, 160.4 MHz, 298 K) of compound **10**.

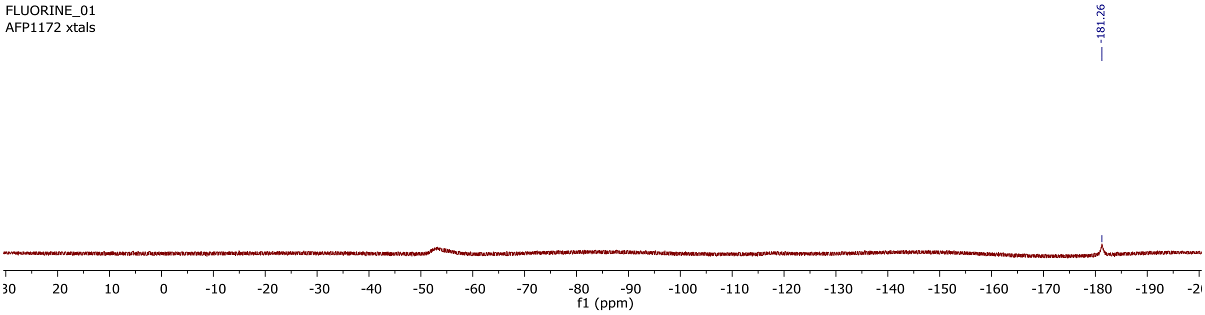

Figure S8: ^{19}F{^{1}H} NMR spectrum (toluene-d_8, 470.4 MHz, 298 K) of compound **9**.

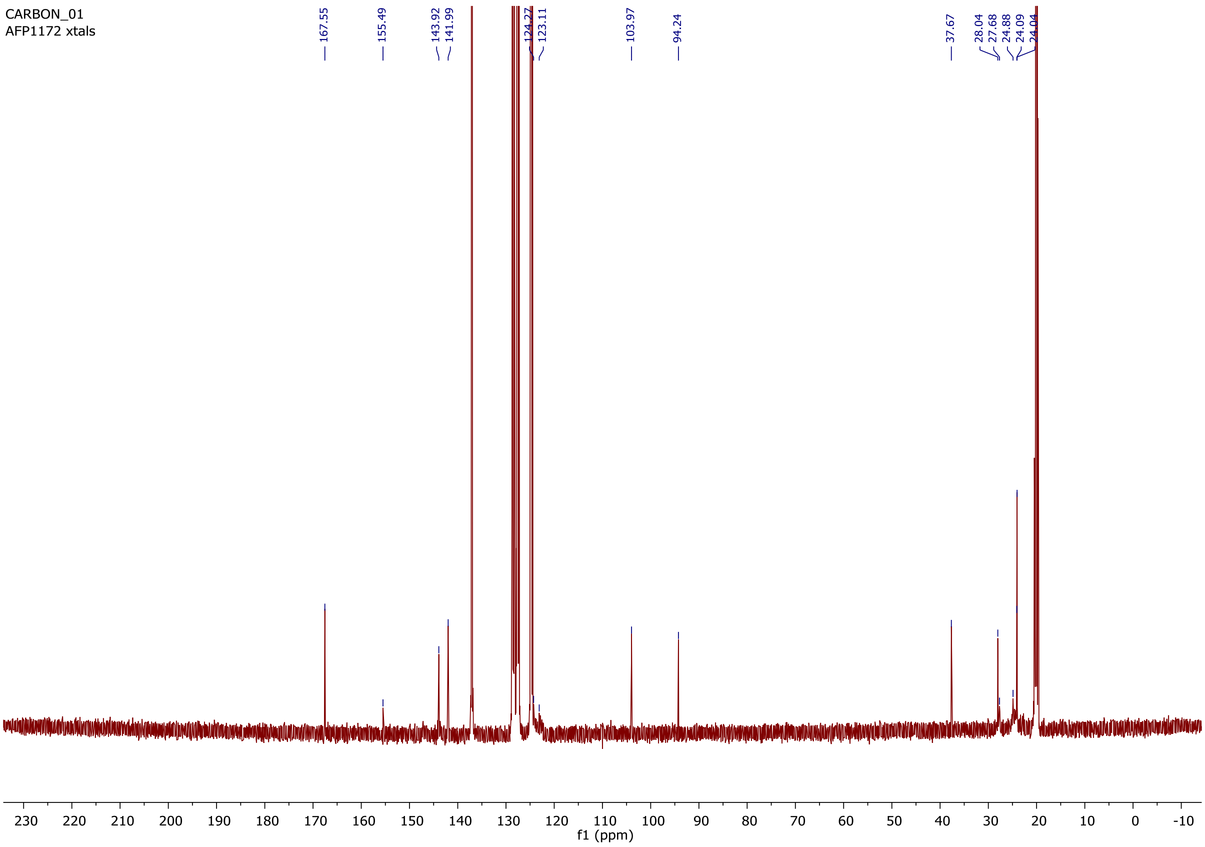

Figure S9: $^{13}C\{^{1}H\}$ NMR spectrum (toluene-d_8, 126 MHz, 298 K) of compound **9**.

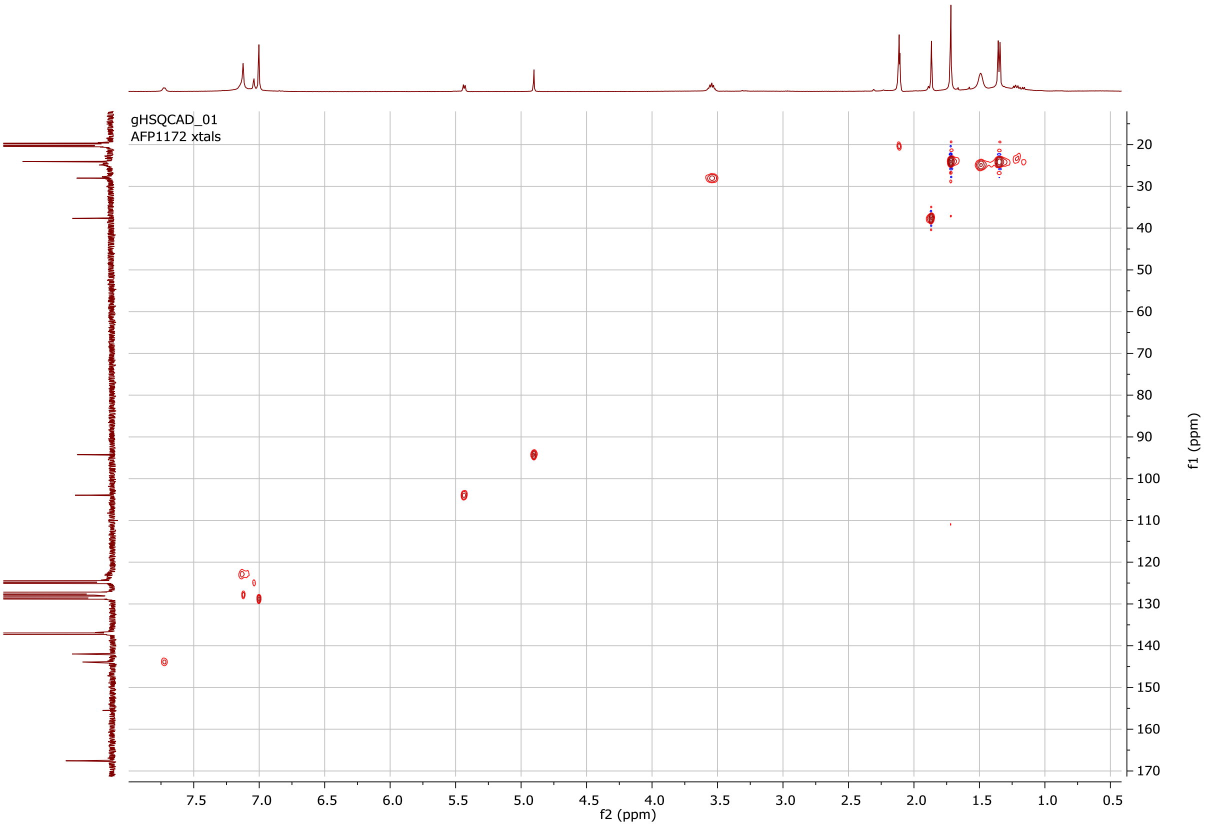

Figure S10: ^{1}H-^{13}C HSQC NMR spectrum (toluene-d_8, 298 K) of compound **9**.

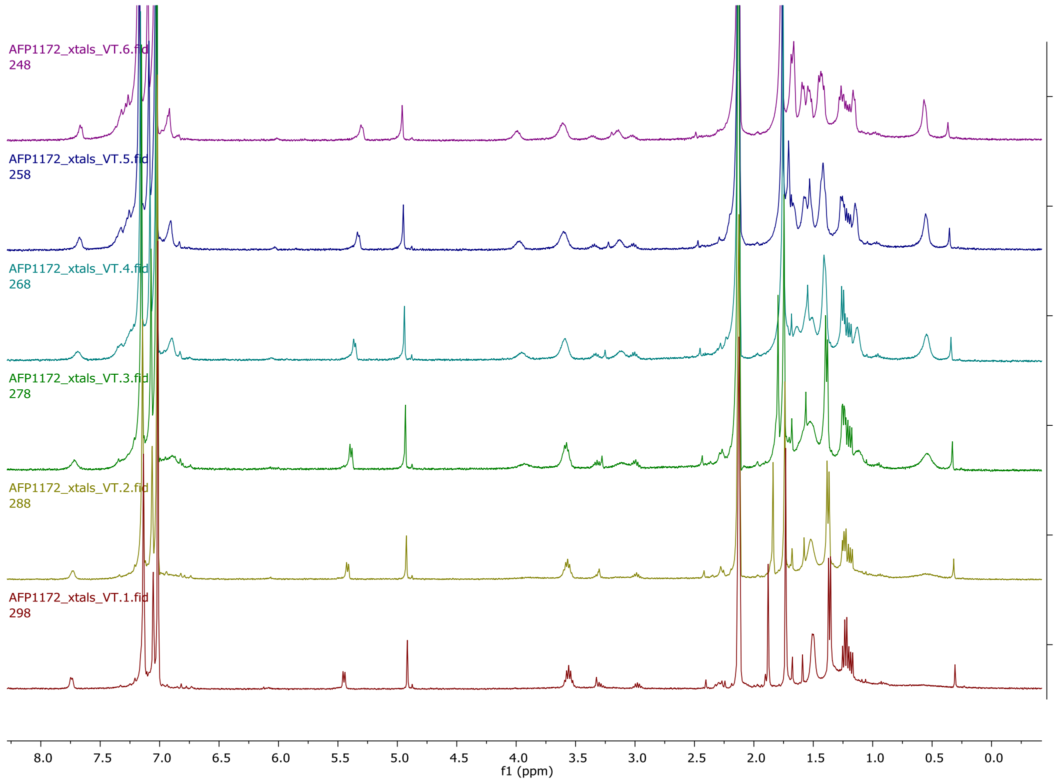

Figure S11: Variable temperature ^{1}H NMR spectra (toluene-d_8, 248 – 298 K, 400 MHz) of compound **9**.

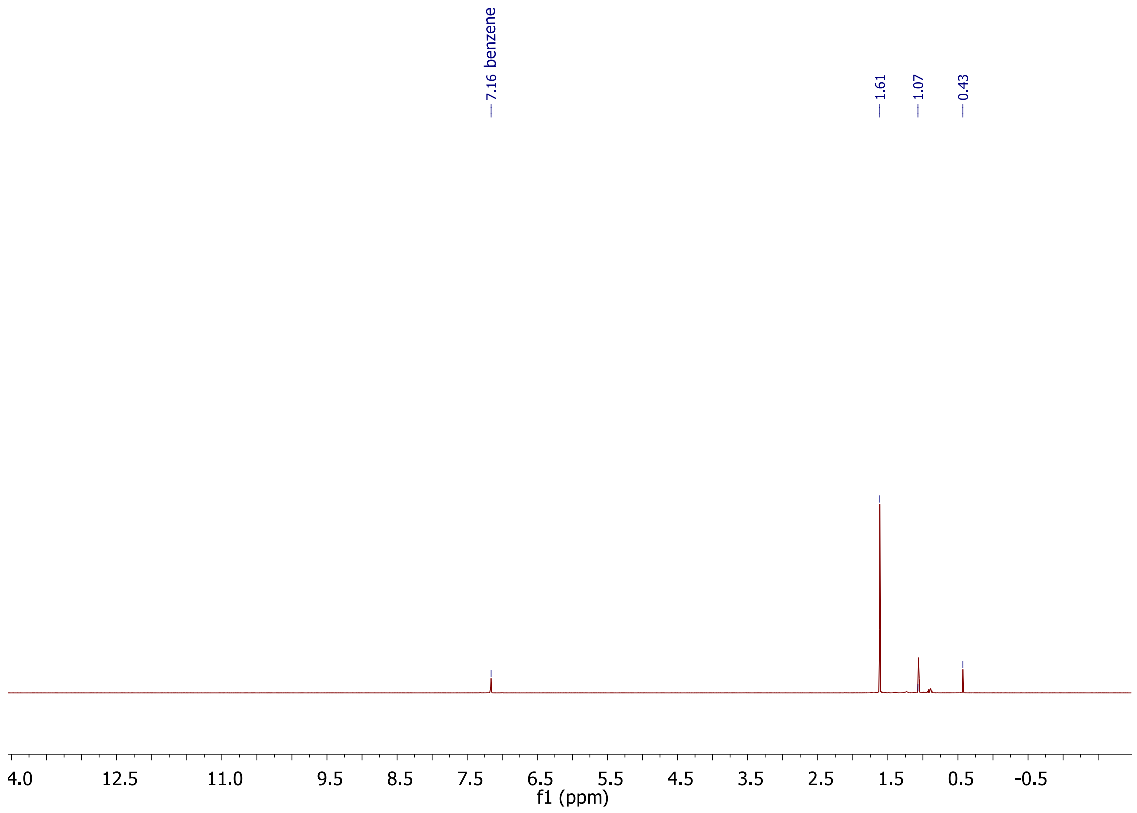

Figure S12: ^{1}H NMR spectrum (C_6D_6, 500 MHz, 298 K) of the volatile components resulting from the reaction of compound **4** and Mes_2BF showing the singlet resonance of 2,3-dimethyl-2-butene at δ 1.61 ppm.

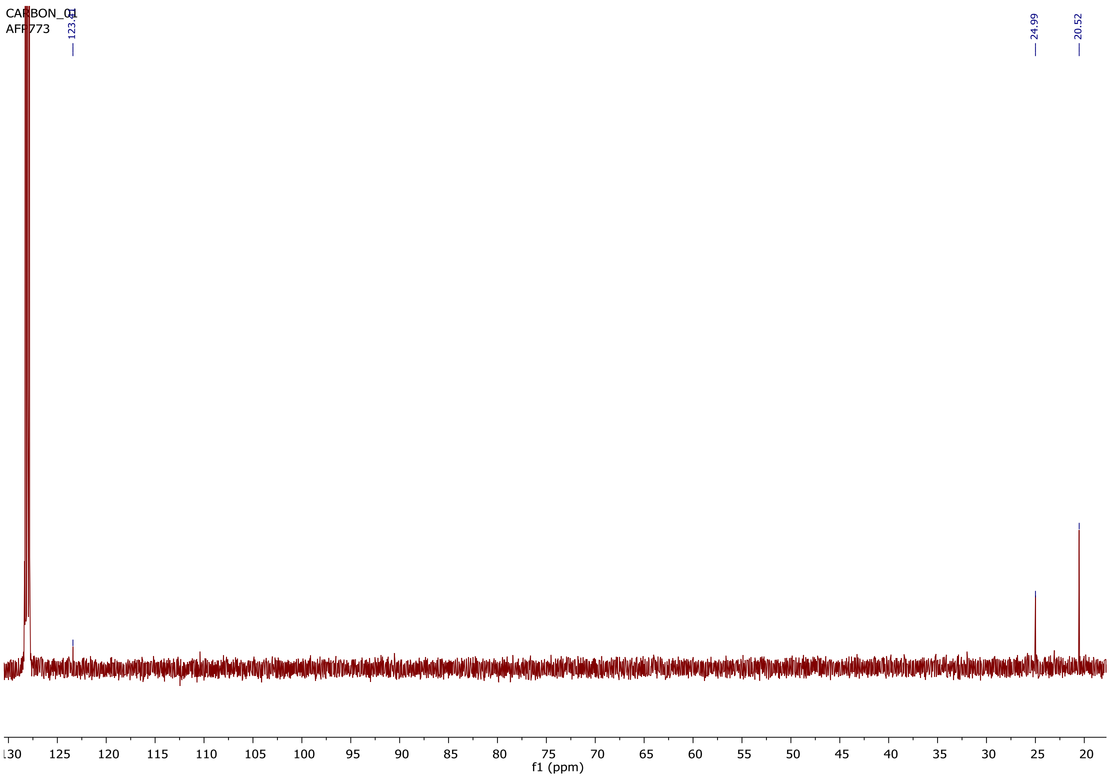

Figure S13: $^{13}C\{^1H\}$ NMR spectrum (toluene-d_8, 126 MHz, 298 K) of 2,3-dimethyl-2-butene after vacuum transfer of the volatile components resulting from the reaction of compound **4** and Mes_2BF.

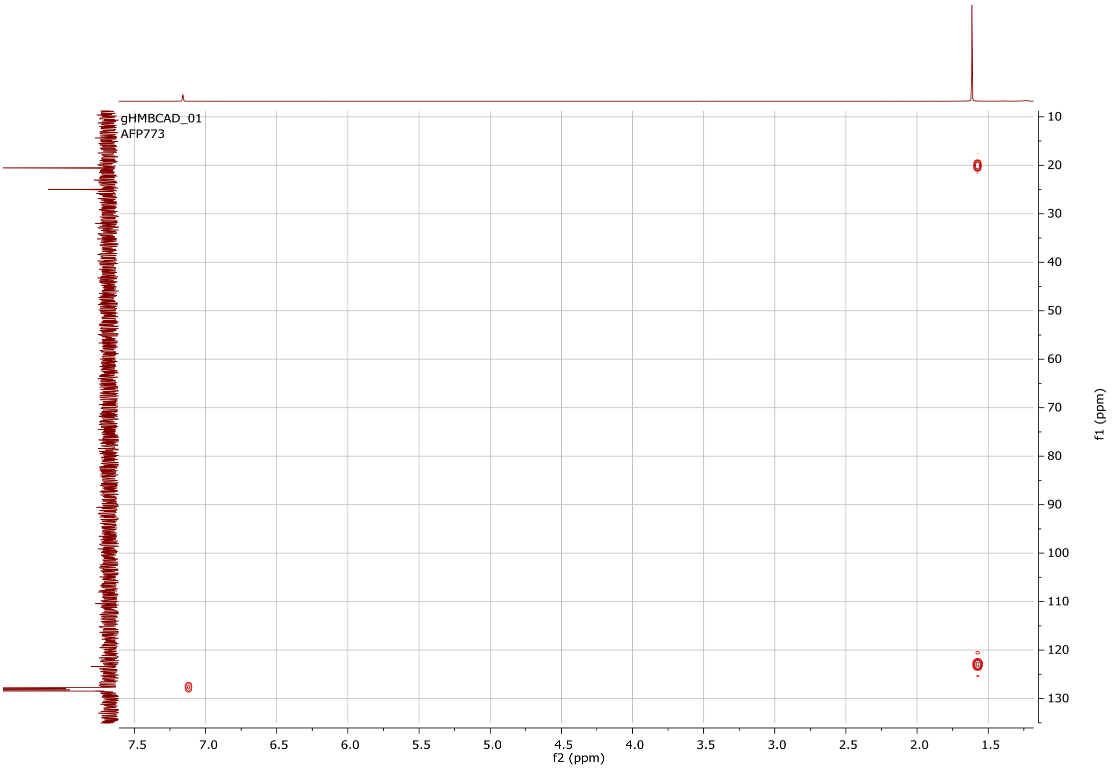

Figure S14: ^1H-^{13}C HSQC NMR spectrum (toluene-d_8, 298 K) of 2,3-dimethyl-2-butene after vacuum transfer of the volatile components resulting from the reaction of compound **4** and Mes_2BF.

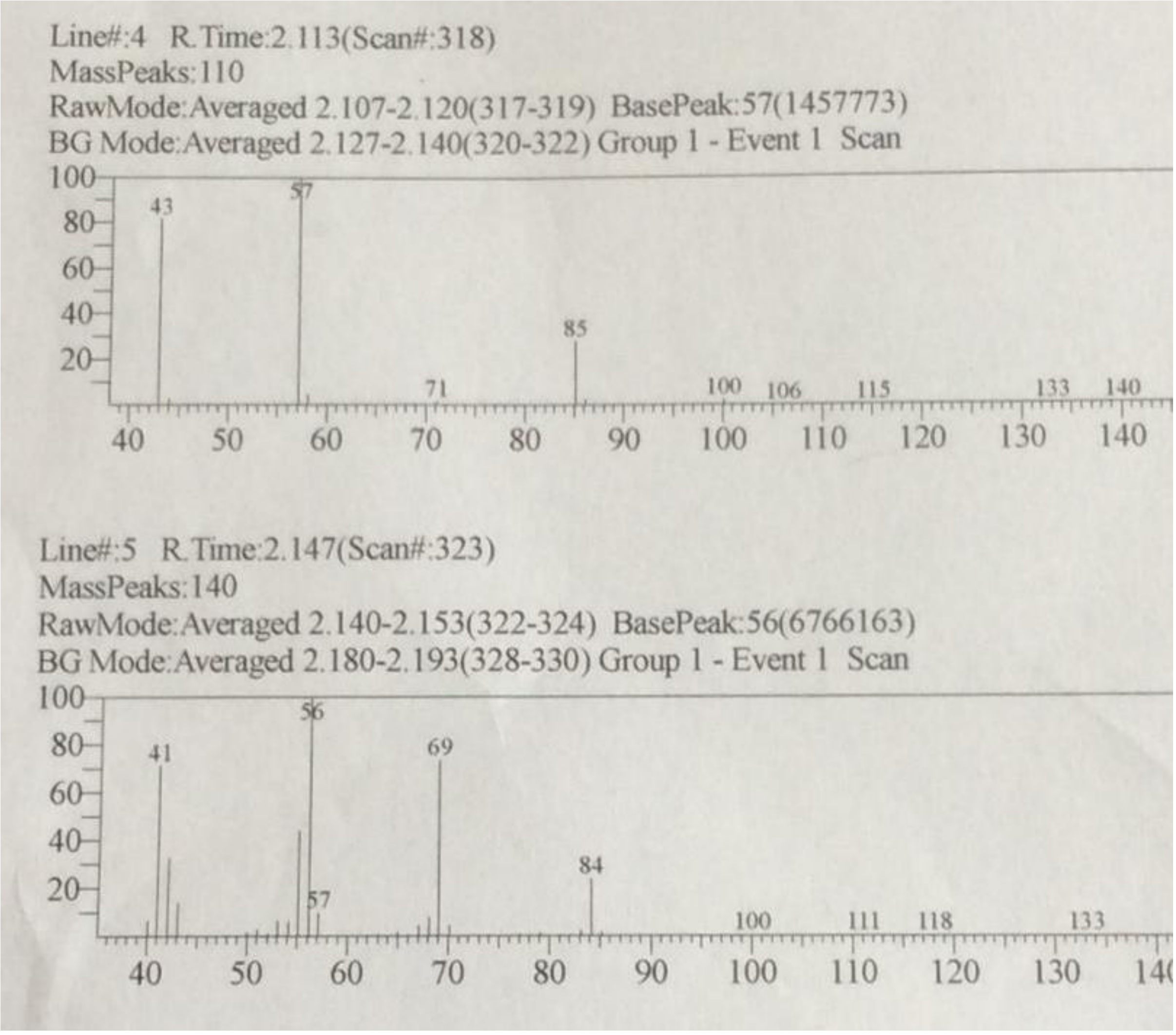

Figure S15: GCMS output trace sampled from the NMR solution of 2,3-dimethyl-2-butene (Mr = 84.16) after vacuum transfer of the volatile components resulting from the reaction of compound **4** and Mes_2BF.

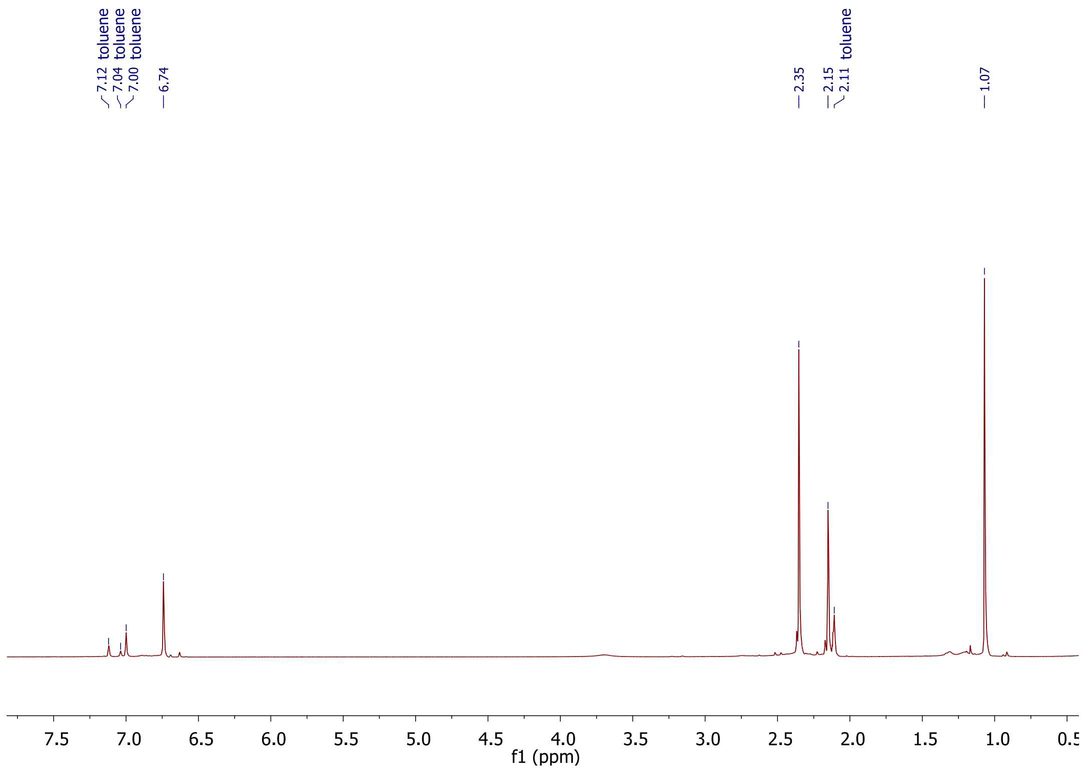

Figure S16: ^{1}H NMR spectrum (toluene-d_8, 500 MHz, 298 K) of pinB-BMes$_2$ (**6**), prepared as described by Yamashita and co-workers from 3MesMgBr and B_2pin_2 in Et_2O.[4]

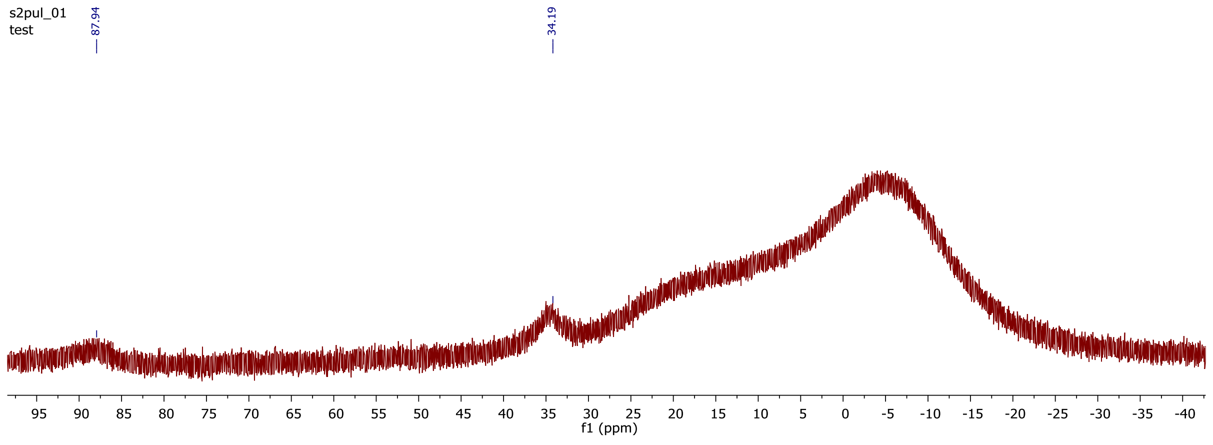

Figure S17: $^{11}B\{^{1}H\}$ NMR spectrum (toluene-d_8, 160.4 MHz, 298 K) of pinB-BMes$_2$ (**6**), prepared as described by Yamashita and co-workers from 3MesMgBr and B_2pin_2 in Et_2O.[4]

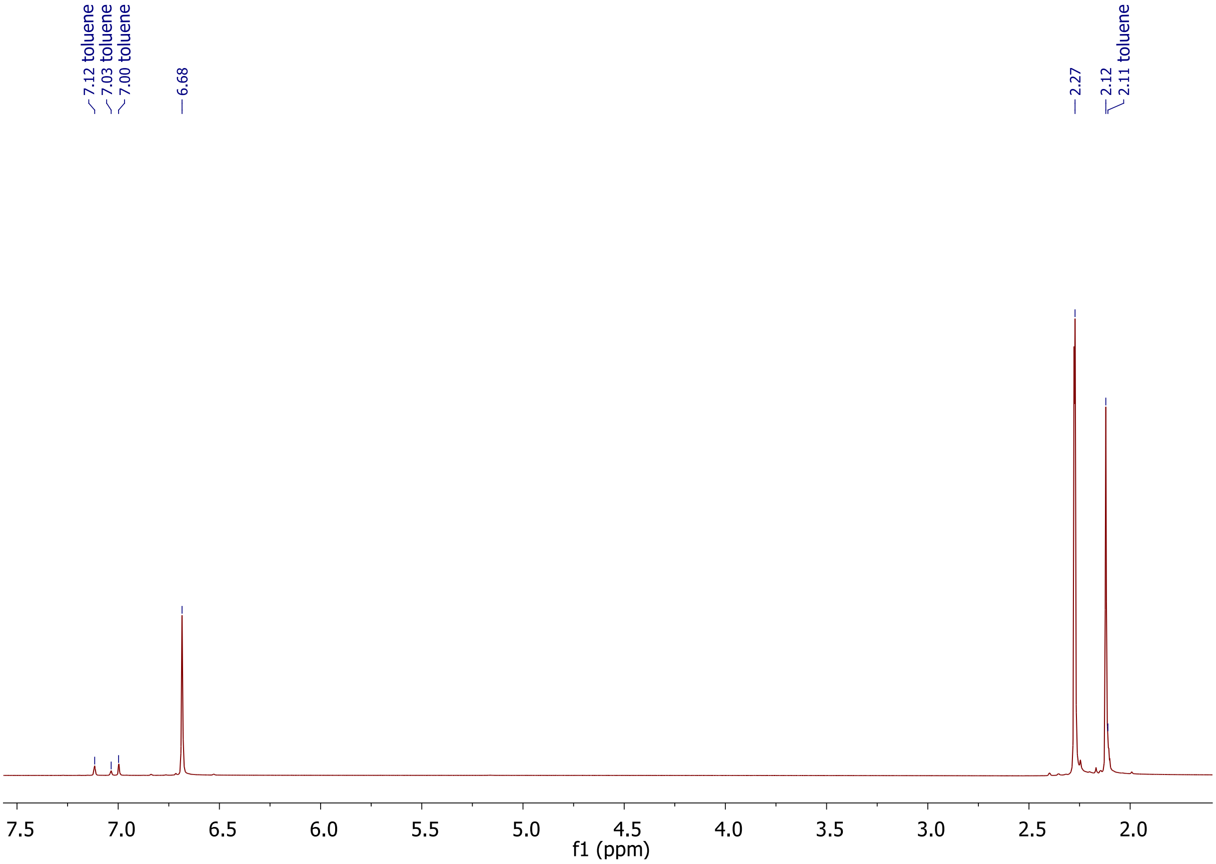

Figure S18: ^{1}H NMR spectrum (toluene-d_8, 500 MHz, 298 K) of Mes_2BF after sublimation.

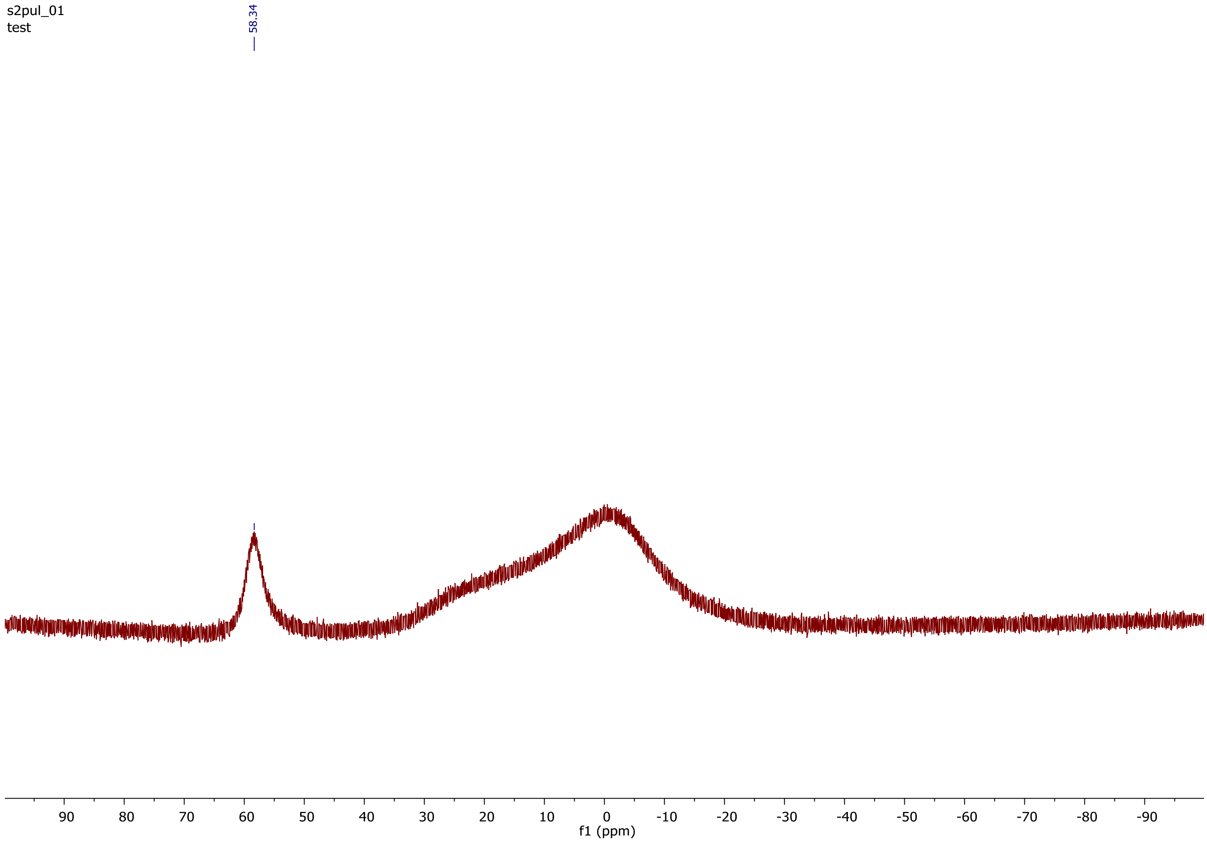

Figure S19: $^{11}B\{^{1}H\}$ NMR spectrum (toluene-d_8, 160.4 MHz, 298 K) of Mes_2BF after sublimation.

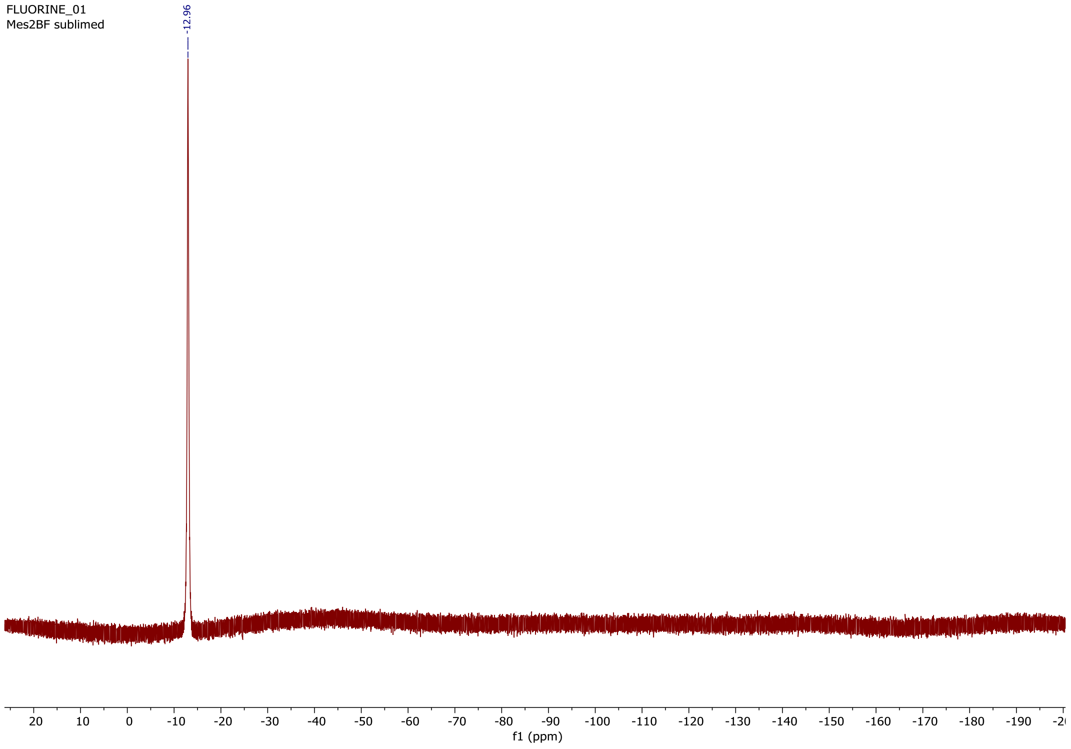

Figure S20: $^{19}F\{^{1}H\}$ NMR spectrum (toluene-d_8, 470.4 MHz, 298 K) of Mes_2BF after sublimation.

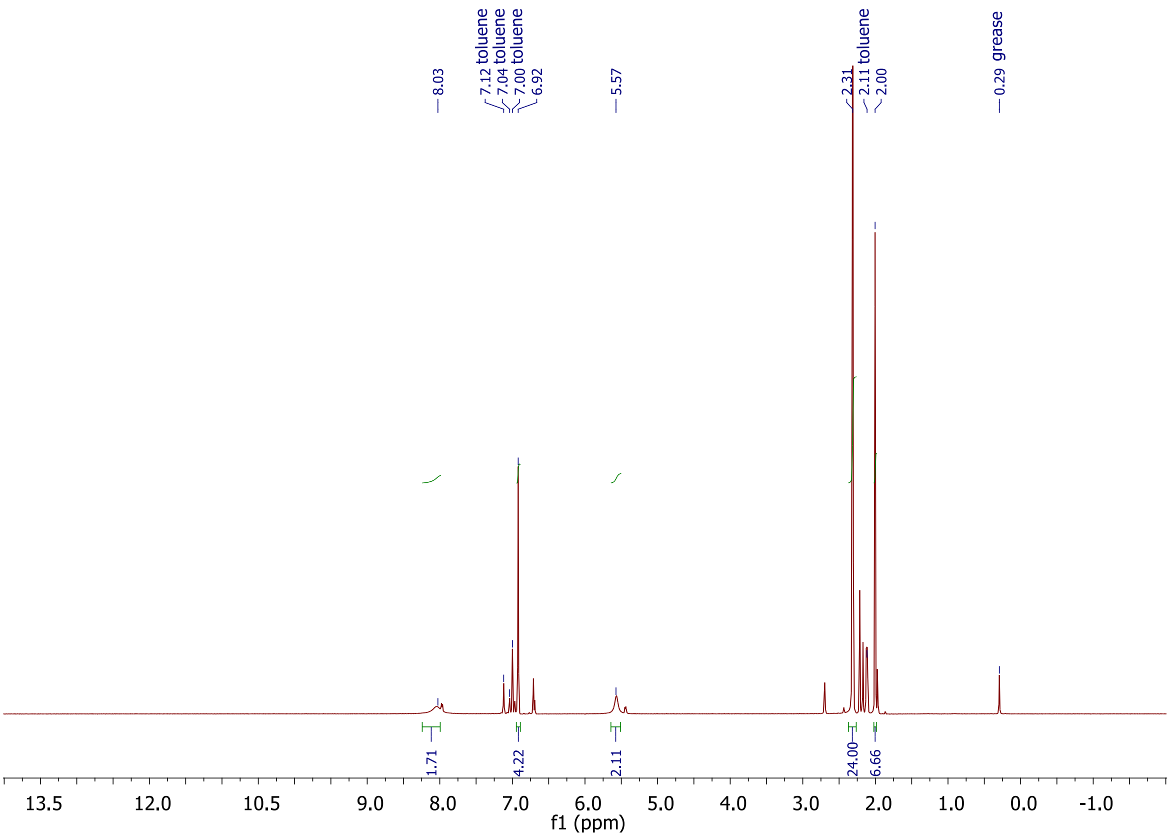

Figure S21: ^{1}H NMR spectrum (toluene-d_8, 500 MHz, 298 K) of the *in situ* reaction of Mes_2BF and 4-dimethylaminopyridine to form $Mes_2BF{\cdot}DMAP$, (**7**).

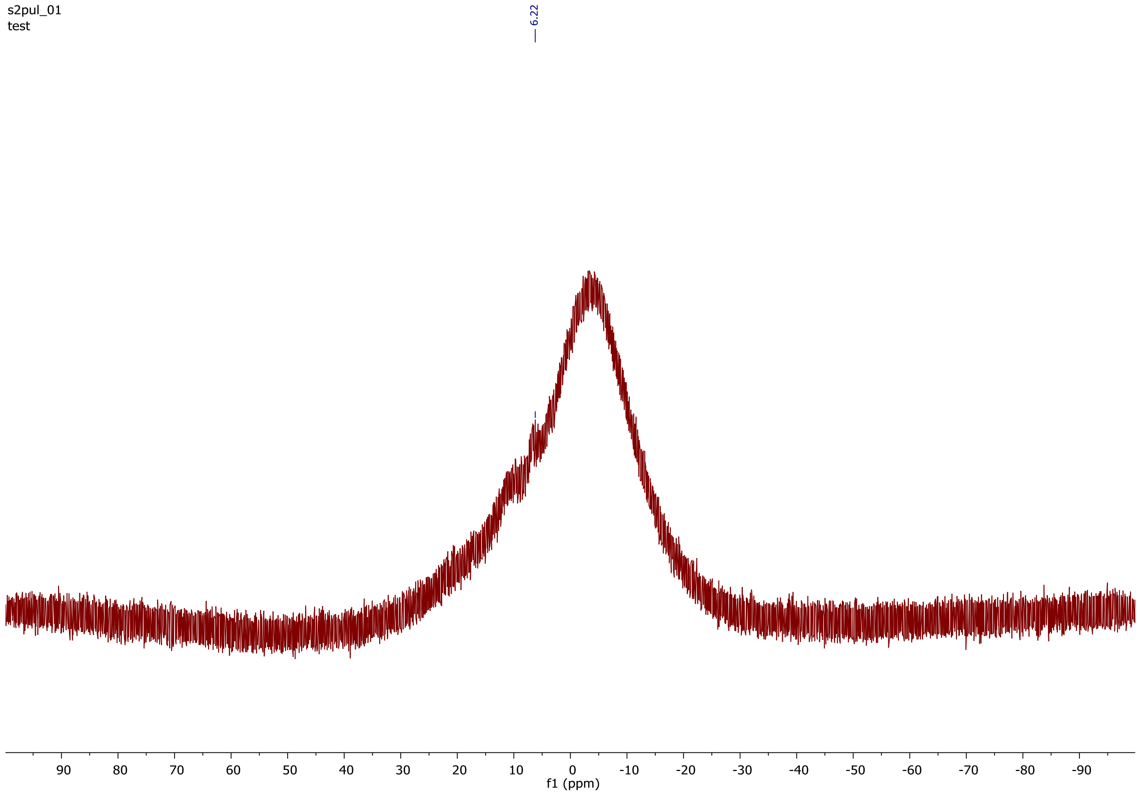

Figure S22: $^{11}B\{^{1}H\}$ NMR spectrum (toluene-d_8, 160.4 MHz, 298 K) of the *in situ* reaction of Mes_2BF and 4-dimethylaminopyridine to form $Mes_2BF{\cdot}DMAP$, (**7**).

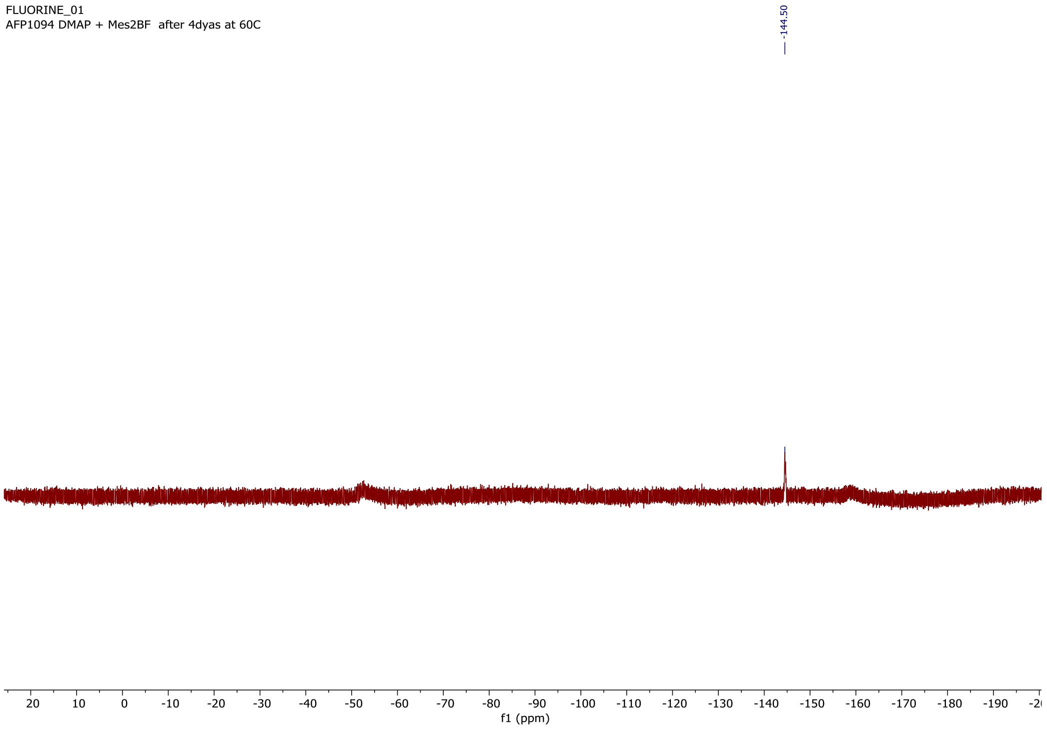

Figure S23: $^{19}F\{^{1}H\}$ NMR spectrum (toluene-d_8, 470.4 MHz, 298 K) of the *in situ* reaction of Mes_2BF and 4-dimethylaminopyridine to form $Mes_2BF{\cdot}DMAP$, (**7**).

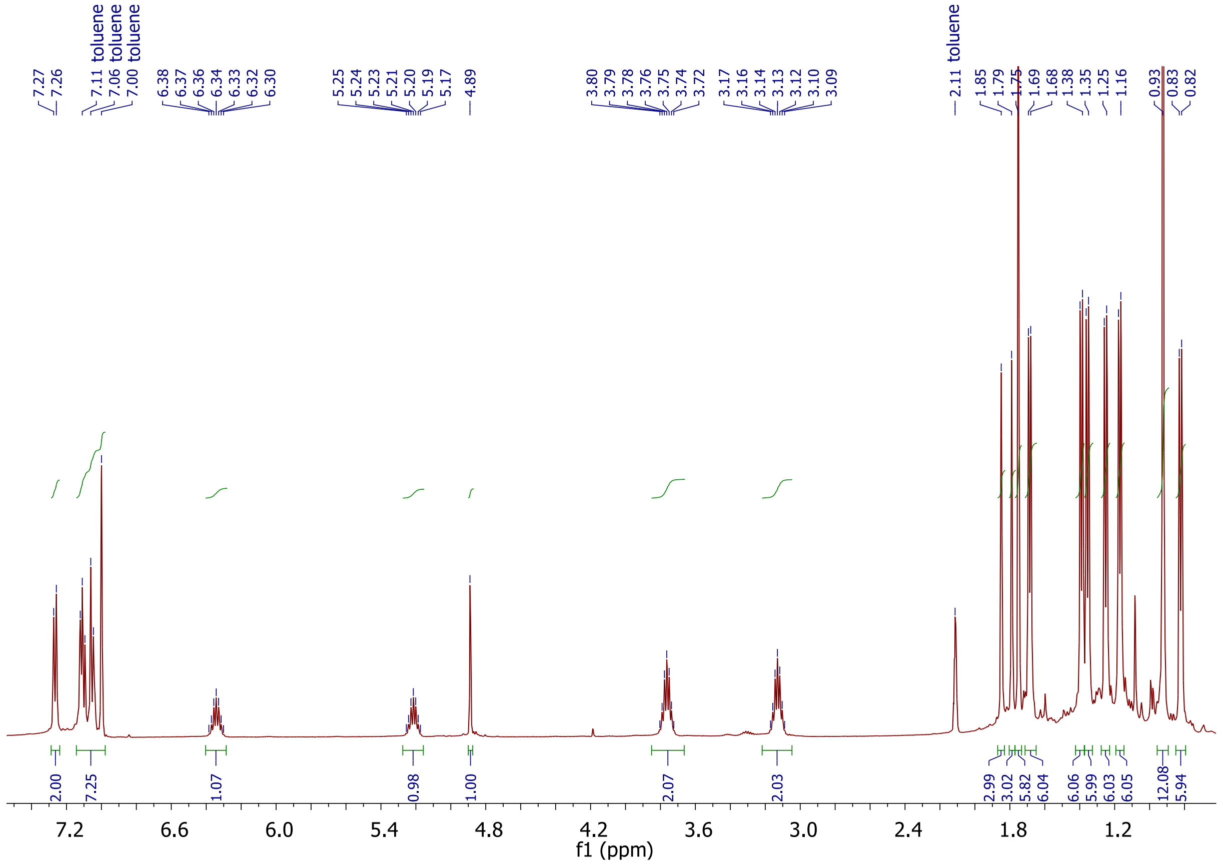

Figure S24: ^{1}H NMR spectrum (toluene-d_8, 500 MHz, 298 K) of compound **13**.

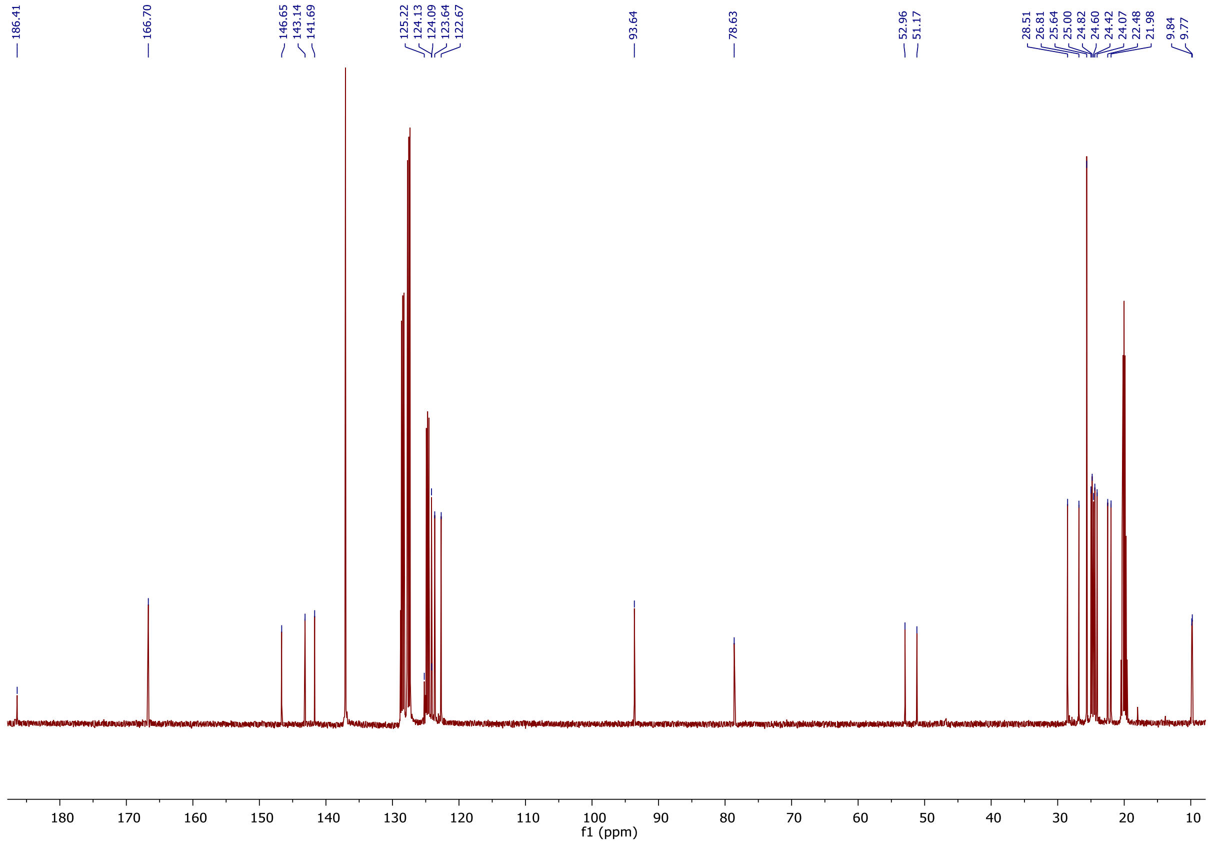

Figure S25: ^{13}C{^{1}H} NMR spectrum (toluene-d_8, 126 MHz, 298 K) of compound **13**.

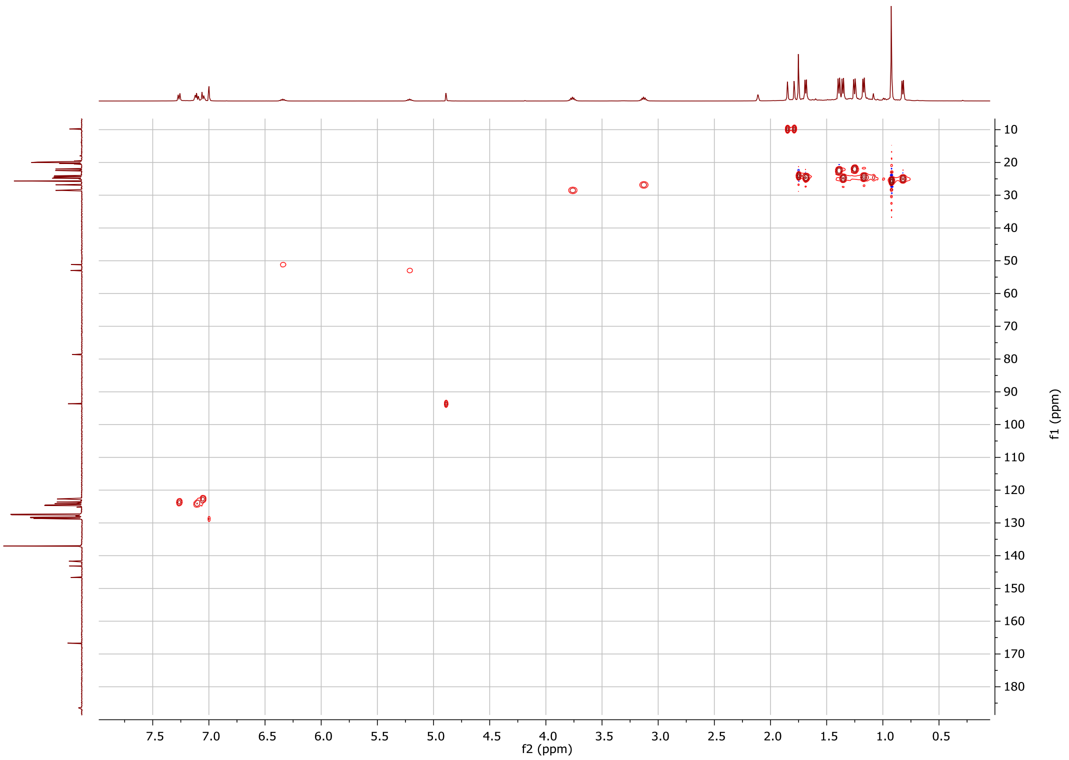

Figure S26: ^{1}H-^{13}C HSQC NMR spectrum (toluene-d_8, 298 K) of compound **13**.

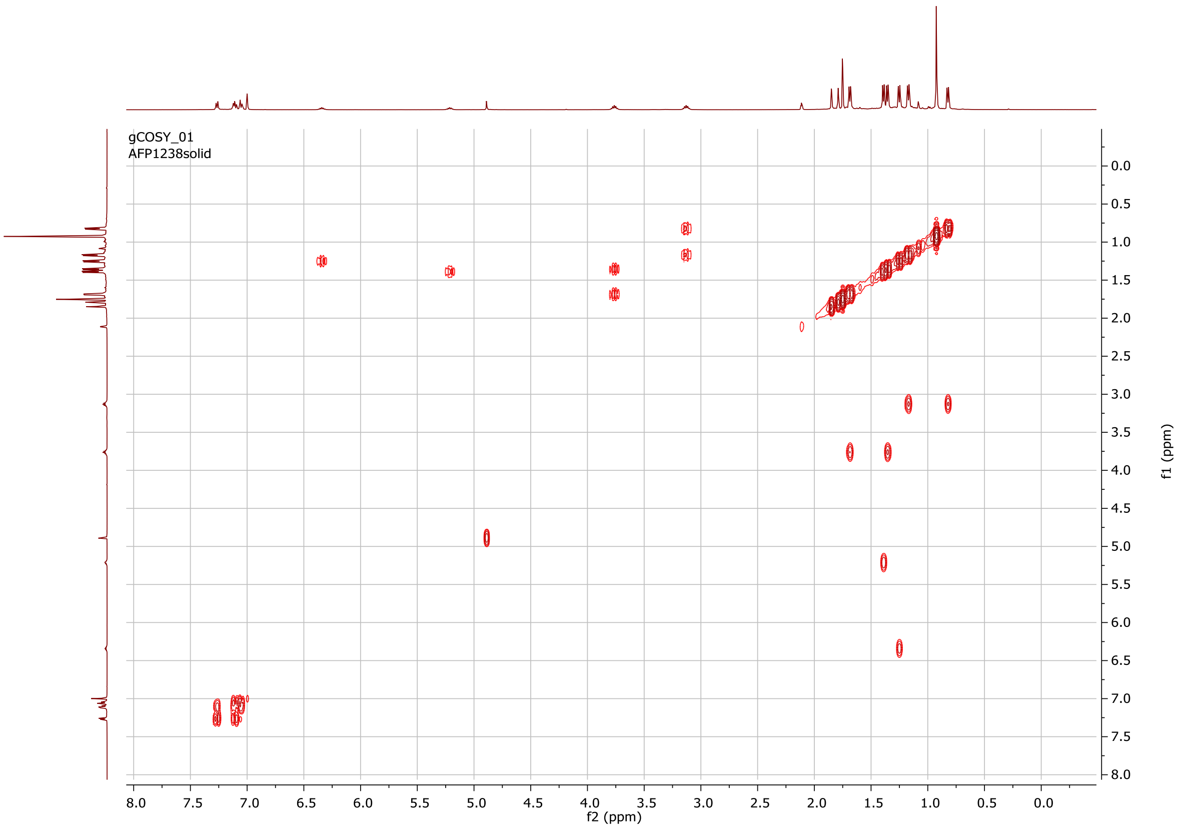

Figure S27: ^{1}H-^{1}H COSY NMR spectrum (toluene-d_8, 298 K) of compound **13**.

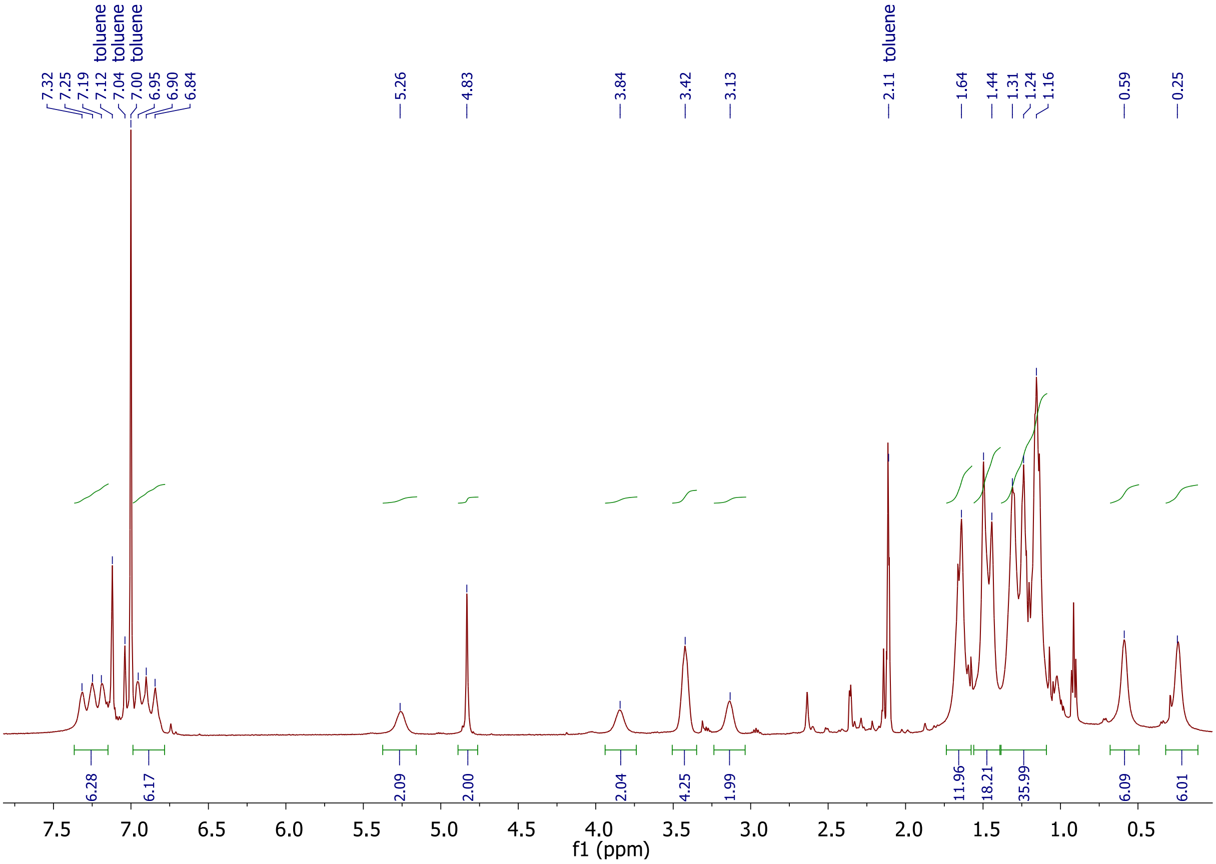

Figure S28: ^{1}H NMR spectrum (toluene-d_8, 500 MHz, 298 K) of compound **15**.

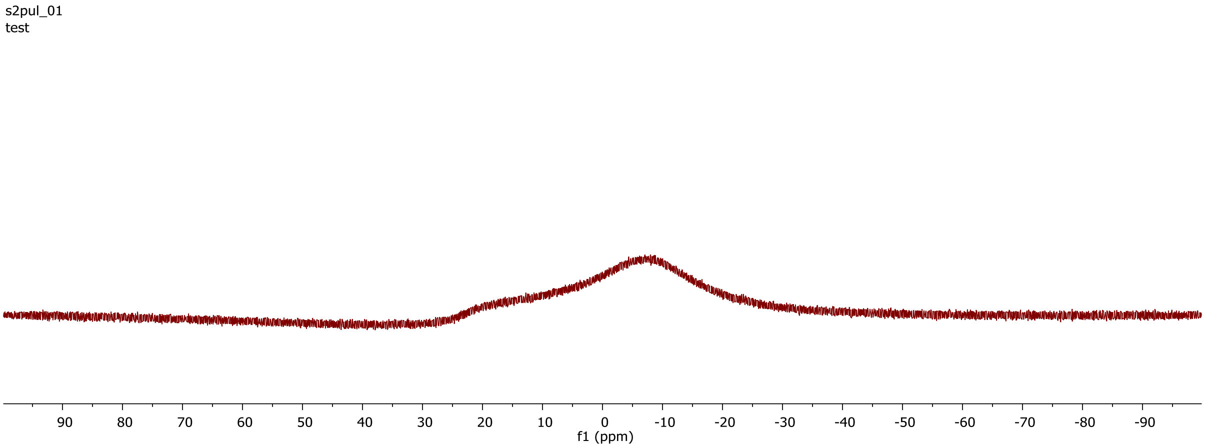

Figure S29: ^{11}B{^{1}H} NMR spectrum (toluene-d_8, 160.4 MHz, 298 K) of compound **15**.

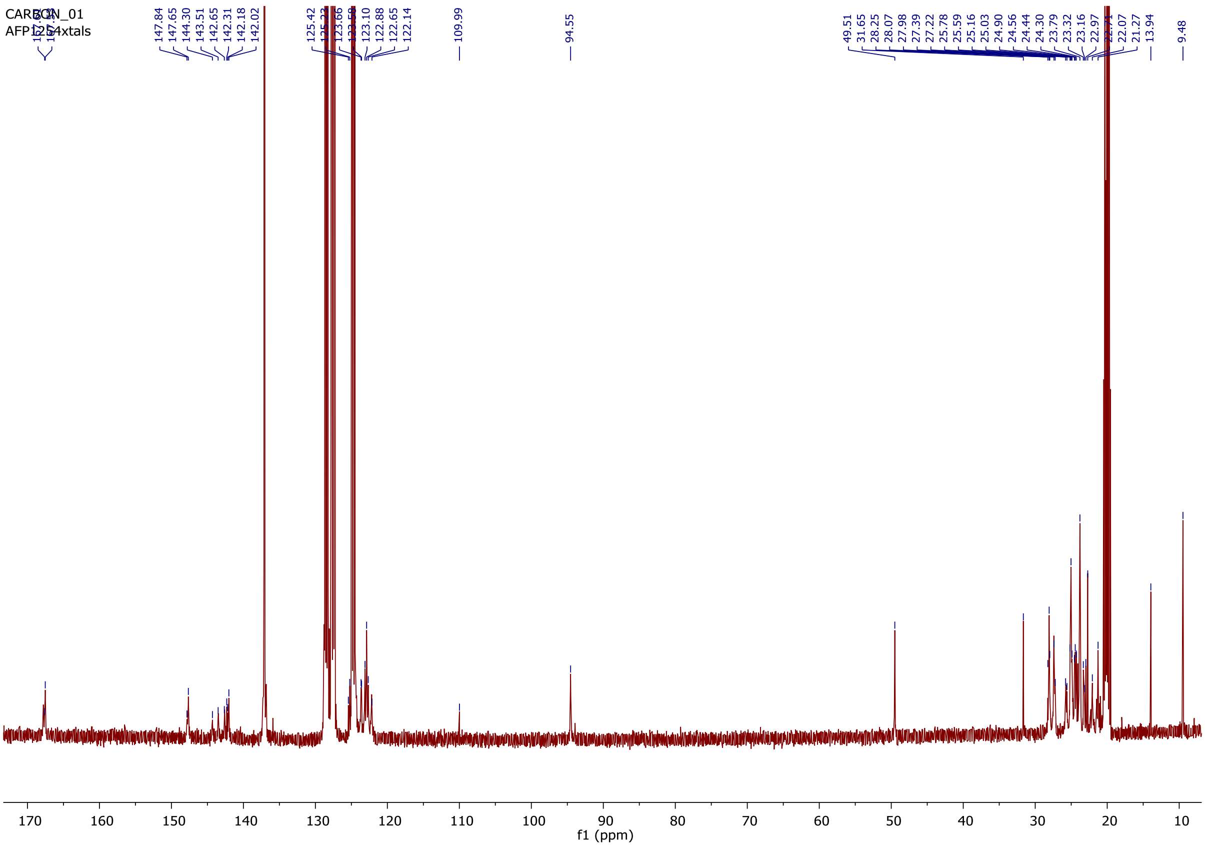

Figure S30: $^{13}C\{^{1}H\}$ NMR spectrum (toluene-d_8, 126 MHz, 298 K) of compound **15**.

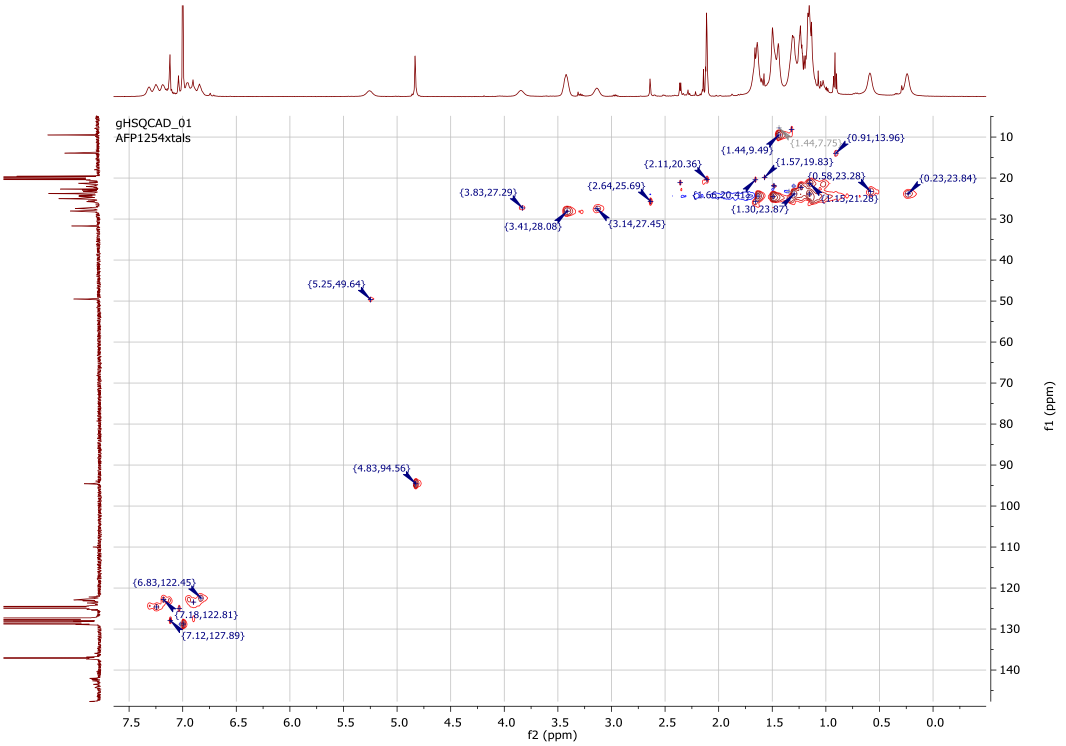

Figure S31: ^{1}H-^{13}C HSQC NMR spectrum (toluene-d_8, 298 K) of compound **15**.

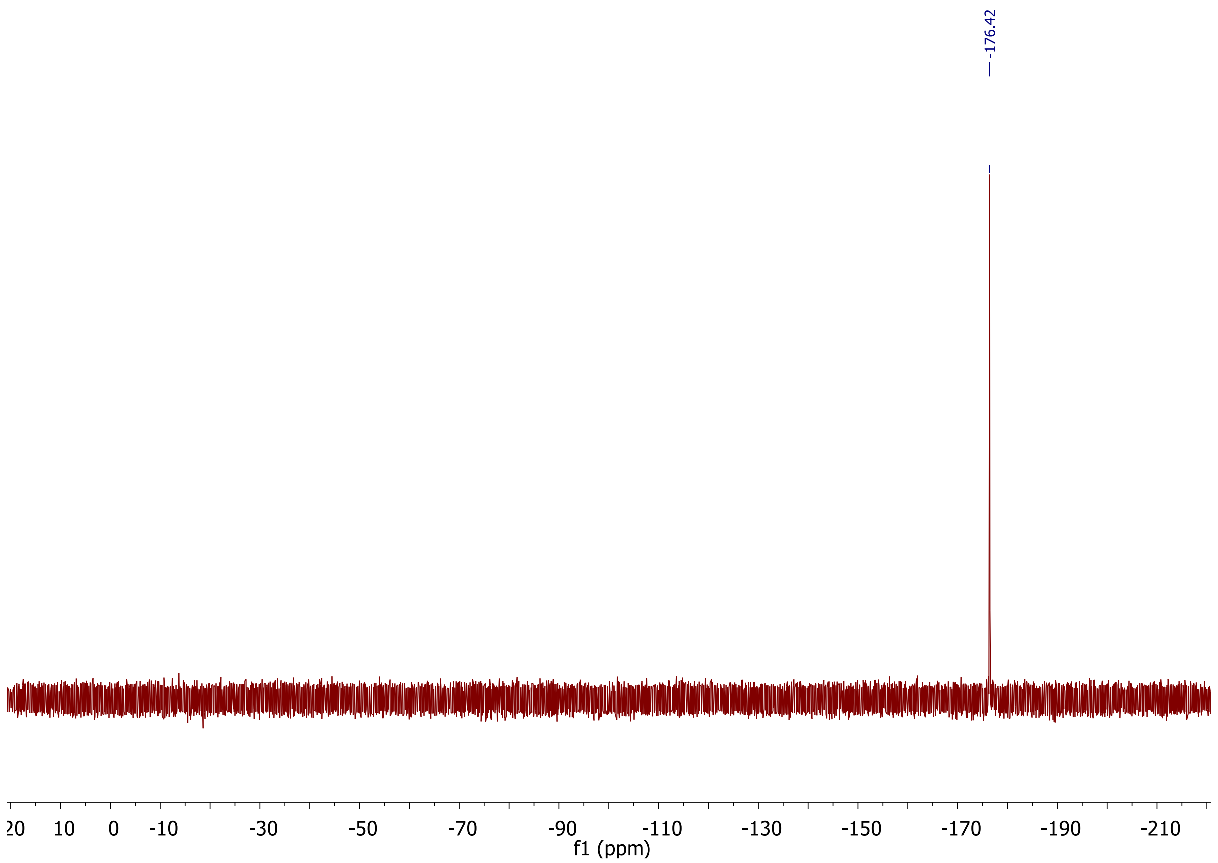

Figure S32: ^{19}F NMR (377 MHz, Toluene-d_8, 298K) NMR spectrum of compound **15**.

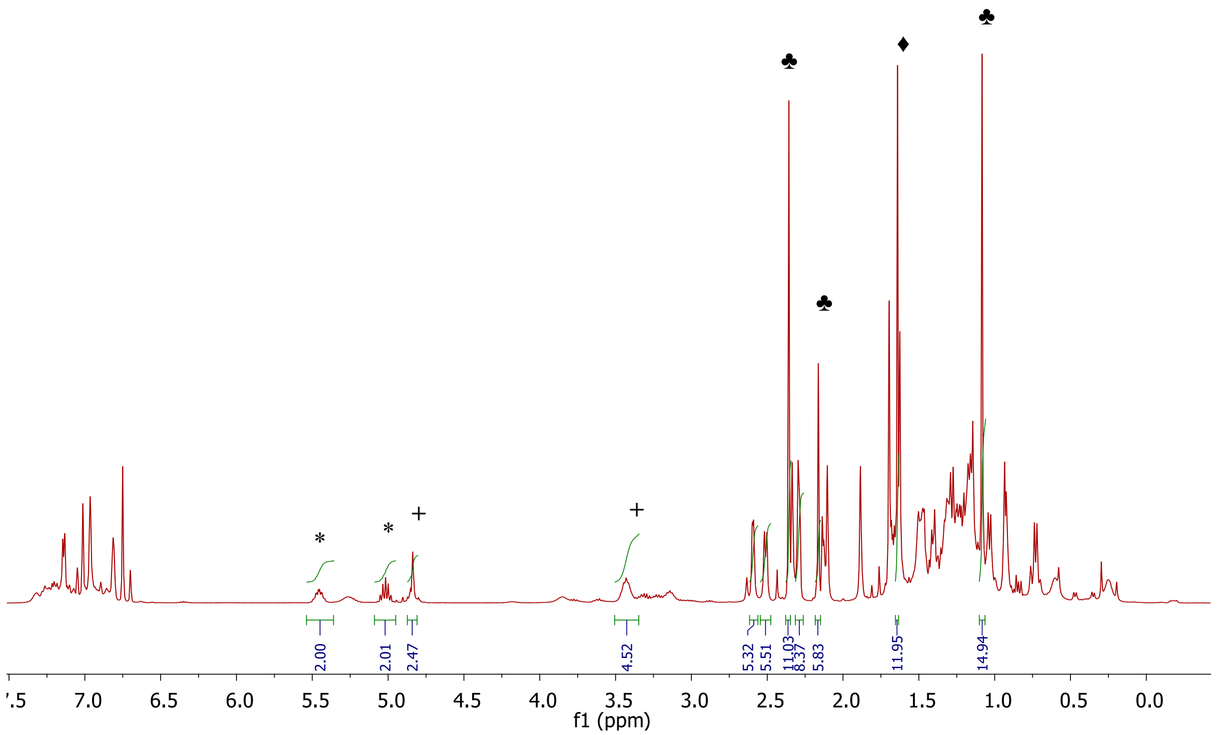

Figure S33: ^{1}H NMR spectrum (toluene-d_8, 500 MHz, 298 K) of the *in situ* reaction of compound **13** and Mes_2BF after one hour at room temerature with selected resonances assigned to pinB-$BMes_2$ (**6,** ♣), Mes_2BF·*i*Pr-NHC (**14**, ∗), as 2,3-dimethyl-2-butene (♦) and compound **15** (+) highlighted.

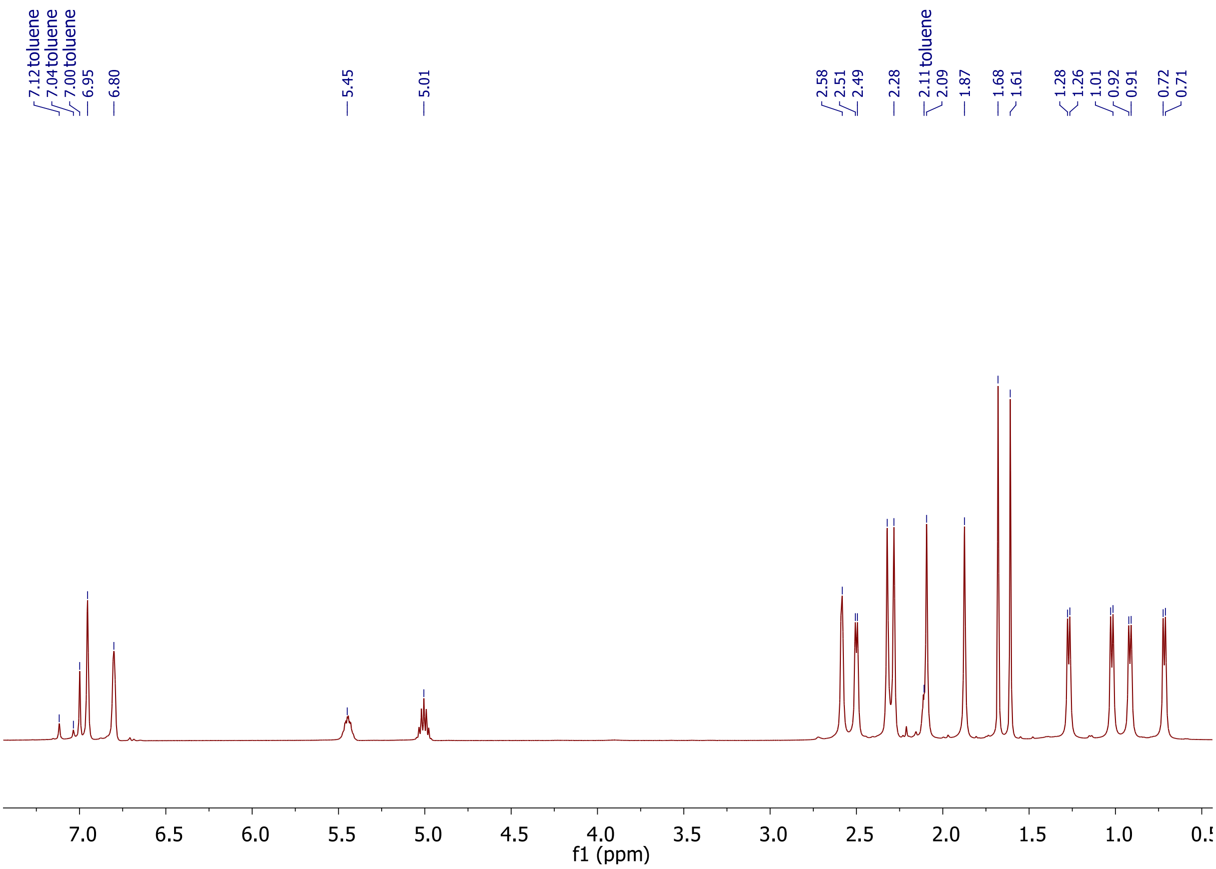

Figure S34: ^{1}H NMR spectrum (toluene-d_8, 400 MHz, 298 K) of the *in situ* reaction of Mes_2BF and 1,3-di-isopropyl-4,5-dimethylimidazol-2-ylidene) to form Mes_2BF·(*i*-Pr-NHC), (**14**).

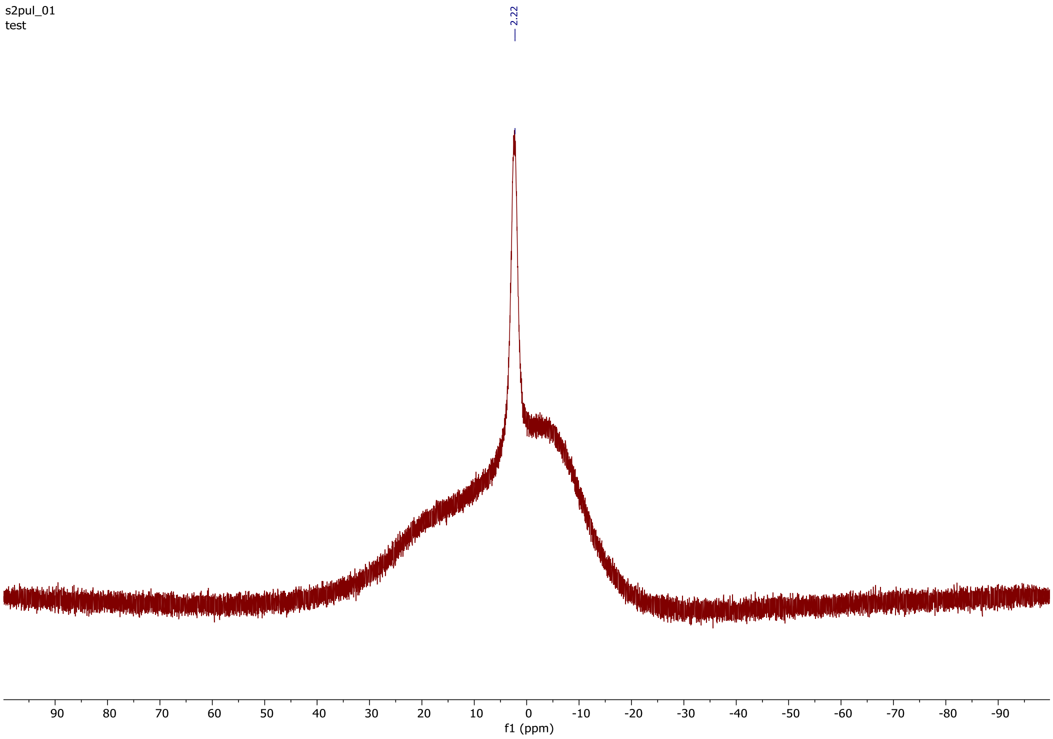

Figure S35: $^{11}B\{^{1}H\}$ NMR spectrum (toluene-d_8, 160.4 MHz, 298 K) of the *in situ* reaction of Mes_2BF and 1,3-di-isopropyl-4,5-dimethylimidazol-2-ylidene) to form Mes_2BF·(*i*-Pr-NHC), (**14**).

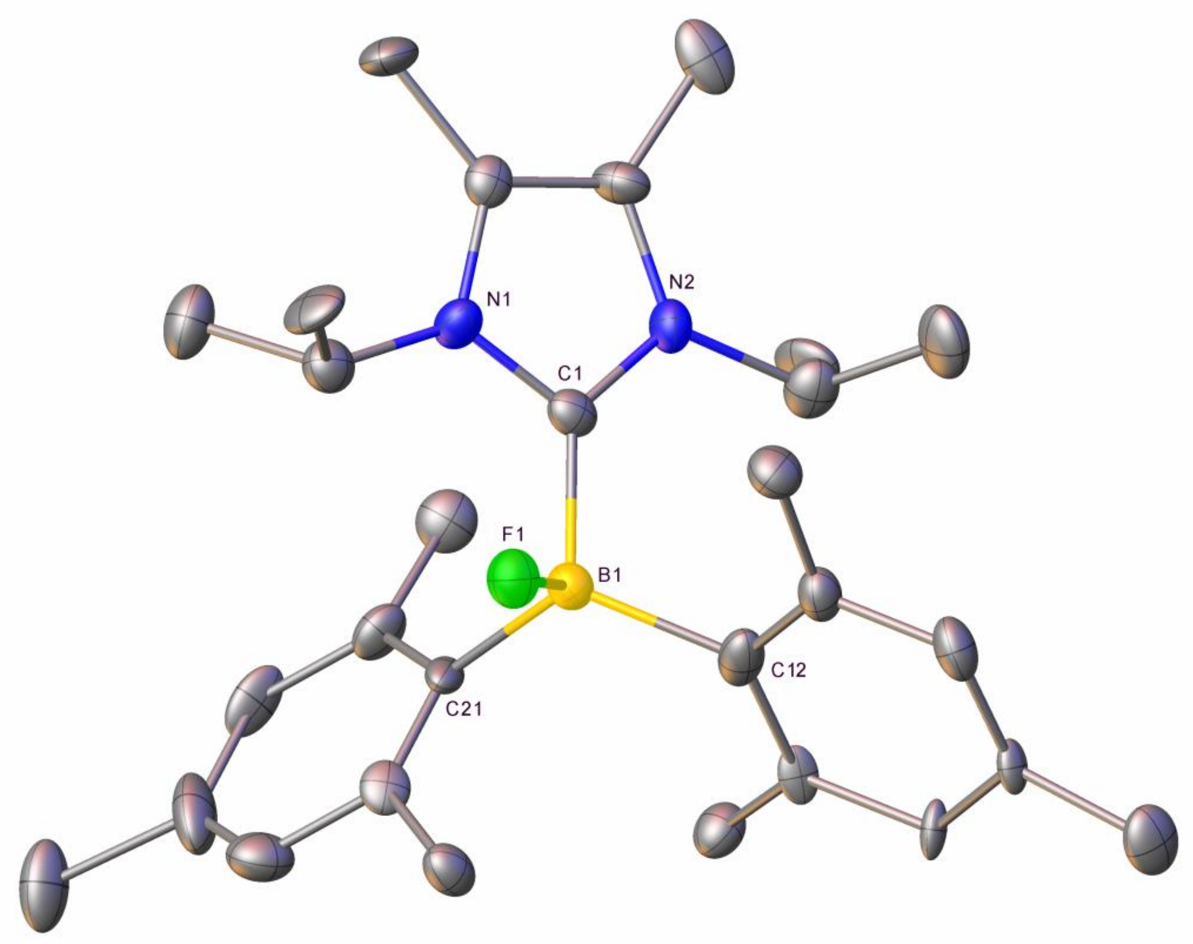

Figure S36: ORTEP representation of compound **14** (30% probability ellipsoids) from a single crystal isolated from the NMR scale reaction of Mes_2BF and 1,3−di−isopropyl −4,5−dimethylimidazol−2−ylidene).

Single Crystal X-ray Diffraction Analysis

Data were collected for compounds **9** and **13 − 15** on a SuperNova, Dual Cu at zero, EosS2 diffractometer. The crystals were all kept at 150(2) K during data collection. Using Olex2,[4] the structures were solved via the olex2.solve routine and refined with the ShelXL[5] refinement package using Least Squares minimization.

The asymmetric unit in **9** comprises two molecules of the magnesium complex, one toluene molecule with 100% site occupancy (based on C139), one toluene moiety with 50% site occupancy and a toluene entity with 90% occupancy that was disordered in a 50:40 ratio over two proximate sites. The latter, disordered region of electron−density, was optimally treated using was the excellent FragmentDB plugin for Olex2, which is a GUI−specific implementation of the DSR refinement package by Kratzert *et al*.[6] The 50% occupancy toluene was also treated with Kratzert's routine as there is evidence of additional electron−density smearing in that region of the motif which did not lend itself readily to disorder modelling.

While the data were of better than average quality for **14**, the raw frames were not quite as beautiful as the R_{int} or R_{sigma} might suggest. Integration yielded the monoclinic setting presented herein and the absence conformed to space groups *Cc* and *C*2/*c*. The crystal was a strong diffractor and, on balance, there was only one serious violation of the *c*−glide systematic absence in the 2 0 3 reflection. Upon solution, it appeared that the model exhibited gross disorder in a 50:50 ratio, which could be modelled in either of the monoclinic *C*, space group options, with the inclusion of relatively few ADP and distance restraints. Alternative space groups without a *c*−glide symmetry element (*C*2 and *Cm*) were also explored, but neither offered any improvement in residuals or, indeed, reduction in disorder. A nagging suspicion about symmetry by virtue of twinning was probed, by integration of the data in a triclinic setting [a = 8.9974(6), b = 9.0233(6), c = 19.2519(12)Å; α = 85.002(5), β = 79.785(5), γ = 60.283(7)°]. As such, the structure was solved in *P*1 with 2 molecules per unit cell, affording an *R*1 of 8.6% (modelling B and F disorder only). Investigation complete, we are presenting the *C*2/*c* solution herein, as the optimal model for this structure, in which all atoms except for C1, were disordered in a 50:50 ratio. Phenyl rings were treated as rigid hexagons.

There is one molecule of the complex in the asymmetric unit of **15**, plus some guest solvent. Isopropyl carbons, C13 and C14, (in the former) were treated to distance and ADP restraints in the process of modelling 65:35 disorder. The solvent was present as 3 distinct entities, all proximate to crystallographic inversion centres. The first of these was half of one molecule of hexane which was ordered and readily modelled. The remaining 2 areas each equated to half of a toluene molecule; however, even with modelling, the electron density in both areas was somewhat smeared. Hence, these regions were ultimately addressed using the solvent mask algorithm available in Olex−2, and allowance for same made in the formula given herein.

Table S1: Single crystal X-ray diffraction analysis of compounds **9** and **13 - 15**.

Compound	**9**	**13**	**14**	**15**
Empirical formula	$C_{146.8}H_{204.2}B_2F_2Mg_4N_{12}O_4$	$C_{46}H_{73}BMgN_4O_2$	$C_{29}H_{42}BFN_2$	$C_{79}H_{117}BFMg_2N_6O_2$
Formula weight	2357.87	749.20	448.45	1261.21
Temperature/K	150.00(10)	150.01(10)	150.00(10)	150.01(10)
Crystal system	triclinic	triclinic	monoclinic	triclinic
Space group	*P*−1	*P*−1	*C*2/*c*	*P*−1
a/Å	19.3606(12)	11.9140(5)	8.9938(6)	12.9318(2)
b/Å	19.9901(10)	12.6590(5)	15.6646(9)	13.3412(2)
c/Å	20.2540(11)	17.7768(9)	19.2553(10)	24.3968(4)
α/°	69.146(5)	87.464(4)	90	92.134(1)
β/°	89.857(5)	79.622(4)	100.205(6)	90.683(1)
γ/°	82.037(5)	62.553(4)	90	115.499(2)
U/Å^3	7245.3(7)	2338.1(2)	2669.9(3)	3794.62(12)
Z	2	2	4	2
ρ_{calc}g cm^{-3}	1.081	1.064	1.116	1.104
μ/mm^{-1}	0.666	0.610	0.527	0.663
F(000)	2554.0	820.0	976.0	1374.0
Crystal size/mm^3	0.138 × 0.081 × 0.06	0.262 × 0.208 × 0.082	0.166 × 0.093 × 0.068	0.298 × 0.169 × 0.134
2θ range for data collection/°	6.138 to 136.498	7.878 to 146.224	9.334 to 146.29	7.256 to 146.342
Index ranges	$-23 \le h \le 23$, $-24 \le k \le 16$, $-24 \le l \le 15$	$-14 \le h \le 14$, $-13 \le k \le 15$, $-21 \le l \le 20$	$-11 \le h \le 10$, $-18 \le k \le 19$, $-23 \le l \le 23$	$-10 \le h \le 16$, $-16 \le k \le 14$, $-30 \le l \le 30$
Reflections collected	45356	17426	10955	52207
Independent reflections, R_{int}	25565 [R_{int} = 0.0510, R_{sigma} = 0.0907]	9150 [R_{int} = 0.0414, R_{sigma} = 0.0665]	2657 [R_{int} = 0.0382, R_{sigma} = 0.0298]	15133 [R_{int} = 0.0291, R_{sigma} = 0.0294]
Data/restraints/parameters	25565/597/1687	9150/0/507	2657/7/286	15133/16/804
Goodness−of−fit on F^2	1.010	1.027	1.155	1.029
Final *R*1, wR_2[I>=2σ (I)]	R_1 = 0.0661, wR_2 = 0.1617	R_1 = 0.0536, wR_2 = 0.1373	R_1 = 0.0841, wR_2 = 0.2097	R_1 = 0.0502, wR_2 = 0.1391
Final *R*1, wR_2[all data]	R_1 = 0.1157, wR_2 = 0.1894	R_1 = 0.0693, wR_2 = 0.1507	R_1 = 0.0928, wR_2 = 0.2153	R_1 = 0.0536, wR_2 = 0.1439
Largest diff. peak/hole / e Å^{-3}	0.57/−0.32	0.30/−0.29	0.31/−0.27	0.83/−0.34

Computational Details / Methodology

DFT calculations were run with Gaussian 09 (Revision D.01).[8] The Mg center was described with the Stuttgart RECPs and associated basis sets,[9] and 6-31G** basis sets were used for all other atoms (BS1).[10] Initial BP86[11] optimizations were performed using the 'grid = ultrafine' option, with all stationary points being fully characterized via analytical frequency calculations as minima (all positive eigenvalues). All energies were recomputed with a larger basis set (BS2) featuring 6-311++G** on all atoms. Corrections for the effect of toluene (ε = 2.3741) solvent were run using the polarizable continuum model and BS1.[12] Single-point dispersion corrections to the BP86 results employed Grimme's D3 parameter set with Becke-Johnson damping as implemented in Gaussian.[13] The BP86-optimized geometries of **9** and **15** were used for NBO (Natural Bond Orbital) studies to generate molecular orbital pictures using GaussView. Computed intermediate geometries were predominately taken from *X*-ray crystallography structure solutions.

Breakdown of Energy Contributions

The following tables detail the evolution of the relative energies as the successive corrections to the initial SCF energy are included. Terms used are:

$\mathbf{\Delta E_{BS1}}$	SCF energy computed with the BP86 functional with BS1
$\mathbf{\Delta H_{BS1}}$	Enthalpy at 0 K with BS1
$\mathbf{\Delta G_{BS1}}$	Free energy at 298.15 K and 1 atm with BS1
$\mathbf{\Delta G_{BS1/tol}}$	Free energy corrected for toluene solvent with BS1
$\mathbf{\Delta G_{BS1/tol+D3}}$	Free energy corrected for toluene and dispersion effects with BS1
$\mathbf{\Delta G_{tol}}$	Free energy corrected for basis set (BS2), dispersion effects and toluene solvent

In each case the final data used in the main article is highlighted in bold.

Table S2: Relative energies (kcal/mol) for computed structures. Data in bold are those used in the main text. All energies are quoted relative to **4** at 0.0 kcal mol^{-1}.

	**ΔE_{BS1}**	**ΔH_{BS1}**	**ΔG_{BS1}**	**$\Delta G_{BS1/tol}$**	**$\Delta G_{BS1/tol+D3}$**	**ΔE_{BS2}**	**ΔG_{tol}**
**3**	14.4	14.8	22.1	0.6	-0.6	15.1	**0.1**
**4**	0.0	0.0	0.0	0.0	0.0	0.0	**0.0**
**8**	-28.0	-26.9	-30.2	264.4	-37.8	-31.4	**-41.3**
**9**	-92.1	-90.9	-83.3	242.8	-106.9	-94.9	**-109.8**
**10**	-7.6	-4.5	17.8	179.6	-20.8	-5.3	**-18.5**
**11**	-51.5	-52.9	-65.7	96.0	-49.9	-56.4	**-54.8**
**12**	-1.6	0.3	4.4	162.9	-1.1	-4.5	**-4.0**
**13**	-0.3	0.2	4.3	153.4	-10.1	3.4	**-6.4**
**15**	-116.1	-113.4	-99.8	197.0	-143.2	-111.7	**-138.7**

Table S3: Reaction formation energies (kcal mol^{-1}) for specific balanced equations. Data in bold are those used in the main text.

	ΔE_{BS1}	ΔH_{BS1}	ΔG_{BS1}	$\Delta G_{BS1/tol}$	$\Delta G_{BS1/tol+D3}$	ΔE_{BS2}	ΔG_{tol}
**Bpin$^-$ → BO_2^- + C_2Me_4**	-67.2	-68.8	-78.4	-82.5	-76.3	-70.4	**-79.5**
(triplet)	*-17.1*	*-18.9*	*-32.6*	*-39.7*	*-33.5*	*-13.9*	***-30.4***
**4 + 2(Mes_2BF) → 7 + 10**	-7.6	-4.5	17.8	179.6	-20.8	-5.3	**-18.5**
**4 → 11 + C_2Me_4**	-51.5	-52.9	-65.7	96.0	-49.9	-56.4	**-54.8**
**10 → 12 + 6**	6.0	4.8	-13.4	-16.7	19.7	0.8	**14.5**
**10 → 0.5(8) + 6**	-15.5	-15.9	-24.8	-24.2	-4.3	-17.6	**-6.4**
**11 + 12 → 9**	-38.9	-38.3	-22.0	-16.2	-56.0	-34.0	**-51.0**
**3 + NHC → 13 + BuBpin**	-14.6	-14.6	-17.8	152.8	-9.5	-11.7	**-6.5**
**2(13) + 2(Mes_2BF) → 15 + 14 + 6 +C_2Me_4**	-115.6	-113.8	-108.4	-109.8	-123.1	-118.4	**-125.9**

	HOMO	LUMO
**9**	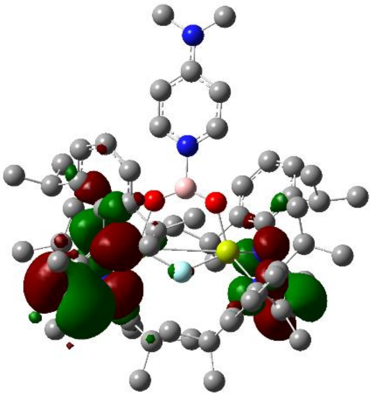	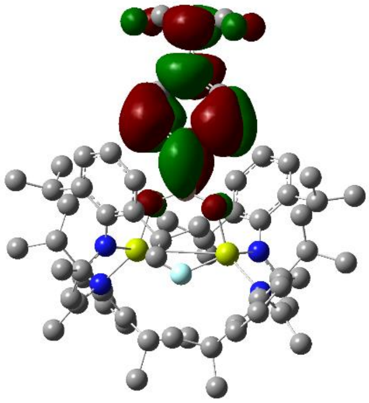
	-0.15112 eV	-0.07554 eV
**15**	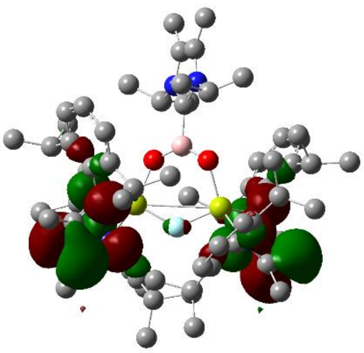	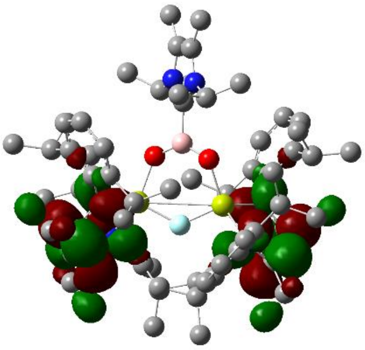
	-0.15324 eV	-0.04523 eV

Figure S37: BP86-optimized geometries showing HOMO and LUMO of complexes **9** and **15** from NBO analysis.

Selected calculated NBO charges:

9: B = +1.127, O(s) = -1.110, Mg(s) = +1.595
15: B = +0.989, O(s) = -1.102, Mg(s) = +1.587

Figure S38: DFT-computed geometries of **9**, **10**, **13** and **15**, relative to **4** and the free substrates. Geometries of singlet and triplet anions [Bpin]$^-$ and [BO_2]$^-$. Bond lengths given in Ångstroms.

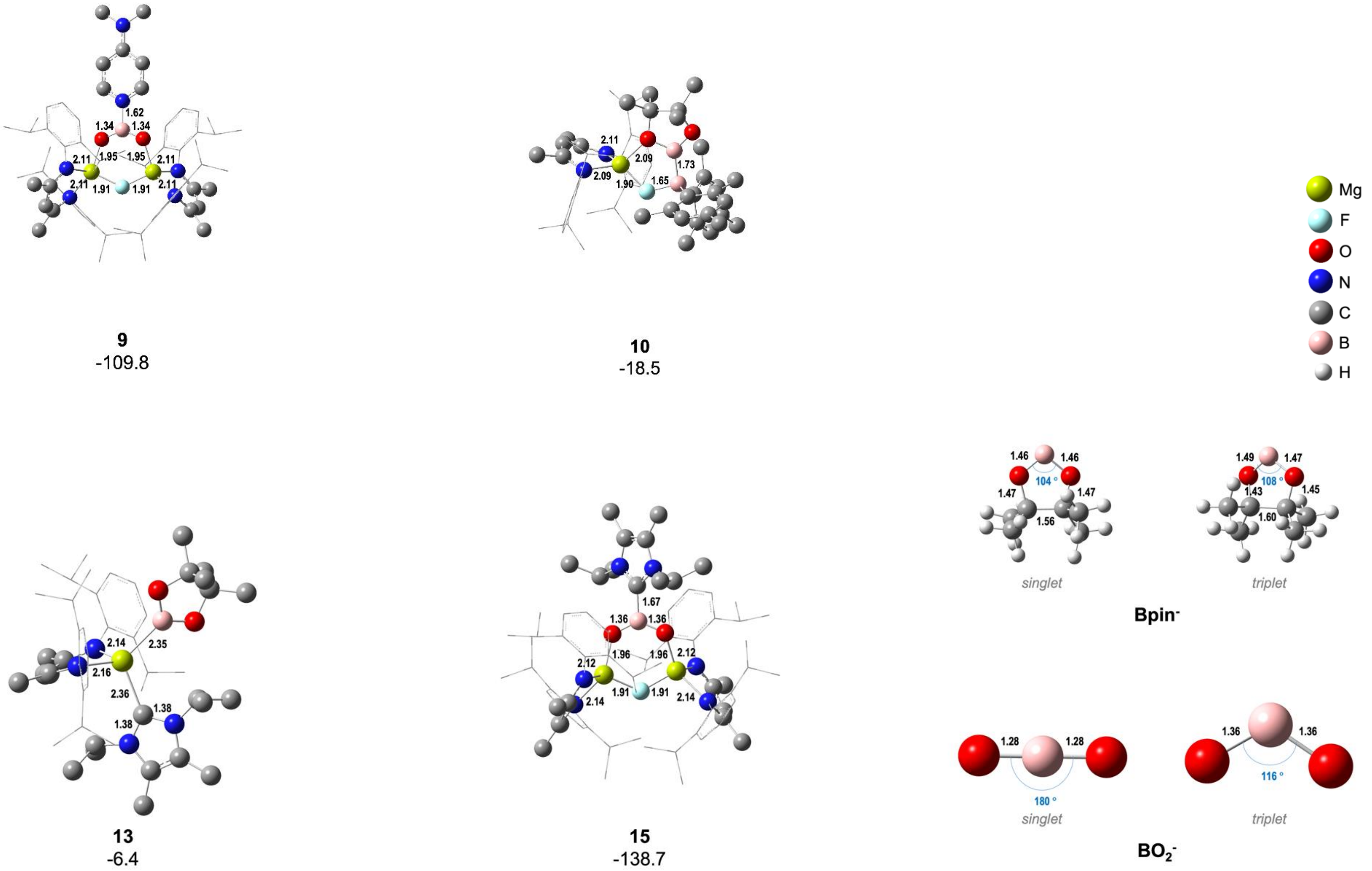

Table S4: Cartesian Coordinates and Calculation Energies

^{1}Bpin^{-}; $BO_2C_2Me_4$
SCF (BP86) Energy = -411.165481229
Enthalpy 0K = -410.995417
Enthalpy 298K = -410.984390
Free Energy 298K = -411.028952
Lowest Frequency = 88.9193 cm^{-1}
Second Frequency = 201.5634 cm^{-1}
SCF (BP86-D3BJ) Energy = -411.198538165
SCF (Toluene) Energy = -411.219801559
SCF (BS2) Energy = -411.309793867

```
B  -0.00023 -2.14165 -0.00061
O   1.06976 -1.24093  0.41543
O  -1.07002 -1.24042 -0.41636
C  -0.77802  0.15019 -0.05380
C  -1.36619  1.08150 -1.12203
H  -2.46490  0.96385 -1.15637
H  -1.14970  2.15172 -0.91220
H  -0.97625  0.84048 -2.12375
C  -1.47527  0.42070  1.29768
H  -1.40644  1.47991  1.62481
H  -2.54310  0.15726  1.20106
H  -1.04079 -0.22632  2.07712
C   1.36635  1.08041  1.12283
H   1.14971  2.15077  0.91397
H   0.97654  0.83841  2.12436
H   2.46509  0.96288  1.15697
C   1.47542  0.42157 -1.29740
H   1.40683  1.48111 -1.62349
H   2.54319  0.15778 -1.20105
H   1.04079 -0.22454 -2.07753
C   0.77809  0.14992  0.05382
```

$^{1}BO_2^{-}$
SCF (BP86) Energy = -175.422351659
Enthalpy 0K = -175.414211
Enthalpy 298K = -175.410212
Free Energy 298K = -175.430504
Lowest Frequency = 545.5699 cm^{-1}
Second Frequency = 1065.6829 cm^{-1}
SCF (BP86-D3BJ) Energy = -175.423943697
SCF (Toluene) Energy = -175.482730452
SCF (BS2) Energy = -175.515933186

```
B   0.00000  0.00000 -0.00035
O   0.00000  1.27705  0.00011
O   0.00000 -1.27705  0.00011
```

C_2Me_4
SCF (BP86) Energy = -235.850228797
Enthalpy 0K = -235.690819
Enthalpy 298K = -235.681181
Free Energy 298K = -235.723319
Lowest Frequency = 87.2996 cm^{-1}
Second Frequency = 102.6605 cm^{-1}
SCF (BP86-D3BJ) Energy = -235.871913166
SCF (Toluene) Energy = -235.850692909
SCF (BS2) Energy = -235.906072466

```
C  -0.68066  0.00001 -0.00001
C   0.68067  0.00001 -0.00001
C  -1.53220 -1.25460 -0.00001
H  -2.19845 -1.27163  0.88413
H  -2.19822 -1.27178 -0.88432
H  -0.95734 -2.19129  0.00015
C  -1.53222  1.25459  0.00001
H  -2.19845  1.27164 -0.88415
H  -2.19828  1.27172  0.88430
H  -0.95740  2.19131 -0.00008
C   1.53222  1.25459 -0.00001
H   0.95739  2.19131 -0.00005
H   2.19835  1.27171  0.88423
H   2.19838  1.27166 -0.88422
C   1.53220 -1.25460  0.00001
H   2.19820 -1.27178  0.88434
H   0.95734 -2.19129 -0.00017
H   2.19849 -1.27162 -0.88411
```

3
SCF (BP86) Energy = -2220.57991976
Enthalpy 0K = -2219.487760
Enthalpy 298K = -2219.422702
Free Energy 298K = -2219.583100
Lowest Frequency = 18.9735 cm^{-1}
Second Frequency = 26.5885 cm^{-1}
SCF (BP86-D3BJ) Energy = -2220.90416120
SCF (Toluene) Energy = -2220.58694528
SCF (BS2) Energy = -2420.36376068

```
Mg -0.34476  0.01378 -0.18714
O   1.54140 -2.22807  2.66404
O   0.23112 -0.49946  1.62808
O   2.35893 -3.05123 -0.58702
O   0.47307 -1.78737 -0.98025
N   0.51453  1.72437 -1.09139
N  -2.31856  0.35138 -0.85786
C   0.52128  3.21091 -3.10961
H   1.23137  3.83727 -2.55179
H  -0.27347  3.84981 -3.52516
H   1.06303  2.76611 -3.96307
C  -0.07685  2.11136 -2.23709
C  -1.29747  1.57224 -2.73842
H  -1.55178  1.93263 -3.74029
C  -2.37621  0.92362 -2.08493
C  -3.68846  0.97689 -2.86537
H  -4.44482  0.28346 -2.47490
H  -3.49885  0.74795 -3.92734
H  -4.11693  1.99339 -2.83081
C  -3.58012  0.07622 -0.19956
C  -4.27991  1.13606  0.45445
C  -5.50795  0.85688  1.08681
H  -6.04588  1.67243  1.58322
C  -6.05569 -0.42852  1.09327
H  -7.01385 -0.62228  1.58673
C  -5.36109 -1.46468  0.46179
H  -5.78584 -2.47393  0.46235
C  -4.12896 -1.24233 -0.18402
C  -3.45154 -2.41490 -0.89197
H  -2.39781 -2.12895 -1.05143
C  -4.08901 -2.67981 -2.27744
H  -5.16444 -2.90677 -2.16873
H  -3.61217 -3.54568 -2.76980
H  -3.99583 -1.81357 -2.95154
C  -3.47321 -3.71728 -0.06284
H  -4.49158 -4.13664  0.01548
H  -3.09036 -3.56385  0.95815
H  -2.84912 -4.48910 -0.54735
C  -3.75936  2.57485  0.51098
H  -2.77726  2.60376  0.00859
C  -4.69983  3.55854 -0.22462
H  -5.68564  3.61324  0.27025
H  -4.87252  3.26009 -1.27138
```

H	-4.27065	4.57572	-0.22645
C	-3.56264	3.03956	1.97213
H	-4.51365	3.02218	2.53234
H	-3.18275	4.07550	2.00169
H	-2.84709	2.39704	2.51180
C	1.66127	2.46199	-0.59421
C	1.46942	3.35241	0.50686
C	2.58182	4.02219	1.04927
H	2.43604	4.70200	1.89370
C	3.86843	3.84311	0.53151
H	4.72197	4.36877	0.97182
C	4.04690	2.99050	-0.55883
H	5.05064	2.85487	-0.97631
C	2.96910	2.29261	-1.14167
C	0.07563	3.66519	1.05162
H	-0.55049	2.76348	0.91397
C	-0.57072	4.80491	0.22652
H	0.04535	5.71953	0.28797
H	-1.57765	5.04808	0.60918
H	-0.66802	4.53353	-0.83720
C	0.06676	4.02383	2.55137
H	0.61356	3.27809	3.15151
H	-0.96954	4.07474	2.92381
H	0.52274	5.01173	2.74049
C	3.28221	1.39866	-2.34317
H	2.32990	0.97938	-2.70761
C	4.20878	0.22372	-1.95175
H	4.44417	-0.39762	-2.83533
H	3.76039	-0.42235	-1.18001
H	5.16769	0.59505	-1.55095
C	3.92666	2.19470	-3.50432
H	3.32408	3.07013	-3.79544
H	4.04744	1.54914	-4.39225
H	4.93023	2.56185	-3.22754
C	0.71923	-2.53986	-2.25600
C	1.30645	-1.54898	-3.26490
H	1.45104	-2.03087	-4.24660
H	2.27400	-1.15597	-2.92583
H	0.60746	-0.70550	-3.40091
C	-0.60019	-3.08965	-2.79294
H	-1.26580	-2.26321	-3.08956
H	-1.12198	-3.71574	-2.05601
H	-0.40765	-3.70185	-3.69148
C	1.73639	-3.64723	-1.76936
C	2.84369	-3.97658	-2.77714
H	3.44517	-3.08997	-3.02619
H	2.42159	-4.39293	-3.70824
H	3.51793	-4.73147	-2.34178
C	1.05731	-4.94472	-1.29309
H	0.62269	-5.51401	-2.13197
H	0.26546	-4.73848	-0.55488
H	1.81513	-5.57543	-0.80189
C	-0.37602	-0.86951	2.90607
C	-1.90023	-0.81125	2.79138
H	-2.24597	0.22419	2.62952
H	-2.37059	-1.17054	3.72328
H	-2.28208	-1.42729	1.96252
C	0.10443	0.12876	3.97487
H	1.19142	0.04849	4.12432
H	-0.40125	-0.04188	4.94089
H	-0.12774	1.15469	3.64460
C	0.20857	-2.32100	3.14062
C	0.25527	-2.73479	4.62236
H	0.66183	-3.75681	4.70278
H	-0.75003	-2.73021	5.08018
H	0.91376	-2.06703	5.19770
C	-0.55993	-3.40135	2.34292
H	-0.67724	-3.10688	1.28621
H	-1.56111	-3.60756	2.76108
H	0.02589	-4.33463	2.37468
B	1.63895	-1.29504	1.51821
B	1.53973	-2.08466	-0.04812
C	2.92915	-0.32134	1.71530
H	3.02134	0.42936	0.90451
H	2.79079	0.27020	2.64468
C	4.25417	-1.10467	1.82956
H	4.15187	-1.86253	2.63028
H	4.43212	-1.68159	0.89849
C	5.48588	-0.22655	2.11659
H	5.31879	0.33207	3.05884
H	5.57591	0.54480	1.32605
C	6.79762	-1.02175	2.21680
H	7.66256	-0.36830	2.42856
H	6.74423	-1.77660	3.02190
H	7.00958	-1.56239	1.27647

4

SCF (BP86) Energy = -2033.73527839
Enthalpy 0K = -2032.782167
Enthalpy 298K = -2032.722299
Free Energy 298K = -2032.879997
Lowest Frequency = 6.8807 cm^{-1}
Second Frequency = 11.4325 cm^{-1}
SCF (BP86-D3BJ) Energy = -2034.00405054
SCF (Toluene) Energy = -2033.74238983
SCF (BS2) Energy = -2233.47329677

Mg	0.15023	-0.01532	-0.11615
O	2.27725	-0.08384	2.48866
O	0.09814	0.14180	3.18101
N	0.35464	-1.59923	-1.51504
N	0.53864	1.46365	-1.58736
N	-2.05316	0.11106	0.07780
N	-6.25270	0.37862	0.54207
C	0.18742	-2.55506	-3.80102
H	1.16551	-3.06663	-3.79749
H	-0.02833	-2.22806	-4.82885
H	-0.55552	-3.31294	-3.50315
C	0.20025	-1.37754	-2.83345
C	0.08411	-0.08965	-3.42586
H	-0.08001	-0.10520	-4.50636
C	0.35080	1.20196	-2.89385
C	0.47103	2.32722	-3.91465
H	-0.12086	3.20742	-3.61512
H	0.14699	1.99872	-4.91333
H	1.51785	2.67031	-3.98793
C	0.63809	-2.93939	-1.06496
C	1.99478	-3.35091	-0.90016
C	3.17449	-2.42902	-1.21542
H	2.76031	-1.45159	-1.51871
C	4.05893	-2.20043	0.03128
H	3.48995	-1.73169	0.85092
H	4.90652	-1.53785	-0.21714
H	4.48230	-3.15099	0.40255
C	4.02069	-2.96716	-2.39257
H	4.84102	-2.26759	-2.63161
H	3.41572	-3.10336	-3.30533
H	4.47545	-3.94323	-2.14594
C	2.26012	-4.65125	-0.42848
H	3.30218	-4.96941	-0.30867
C	1.22863	-5.54085	-0.10968
H	1.45731	-6.54862	0.25351
C	-0.09879	-5.12330	-0.25245
H	-0.91144	-5.81143	0.00831
C	-0.41931	-3.83503	-0.72321
C	-1.89220	-3.43524	-0.81830
H	-1.93252	-2.42171	-1.25154
C	-2.70914	-4.37505	-1.73384
H	-2.73711	-5.40468	-1.33544
H	-2.28958	-4.42653	-2.75297
H	-3.75312	-4.02329	-1.81473
C	-2.53212	-3.37151	0.58750

```
H  -3.57126 -3.00184  0.52896
H  -1.96584 -2.70029  1.25331
H  -2.55411 -4.37057  1.05848
C   1.00207  2.77292 -1.20164
C   2.40435  3.02430 -1.11729
C   3.44793  1.95442 -1.44398
H   2.90596  1.04416 -1.75405
C   4.28750  1.60027 -0.19522
H   5.03982  0.83078 -0.44313
H   3.65593  1.21105  0.62069
H   4.83124  2.48522  0.18186
C   4.36438  2.37798 -2.61479
H   4.96047  3.27179 -2.35790
H   3.78643  2.61490 -3.52458
H   5.07173  1.56757 -2.86418
C   2.84491  4.29552 -0.70113
H   3.92200  4.48942 -0.63864
C   1.94262  5.31042 -0.36425
H   2.30665  6.29318 -0.04568
C   0.56962  5.05104 -0.43139
H  -0.14231  5.83887 -0.15832
C   0.07642  3.79714 -0.84206
C  -1.43567  3.57299 -0.86375
H  -1.61150  2.55202 -1.24197
C  -2.16811  4.55792 -1.80294
H  -3.25023  4.33723 -1.82668
H  -1.78791  4.49973 -2.83710
H  -2.05015  5.60271 -1.46498
C  -2.02299  3.65381  0.56378
H  -1.51785  2.94826  1.24360
H  -3.10109  3.41399  0.55806
H  -1.90457  4.66814  0.98521
C  -2.89073  0.13850 -0.99165
H  -2.40773  0.08837 -1.97568
C  -4.27632  0.22506 -0.89436
H  -4.86995  0.24180 -1.81029
C  -4.88936  0.29133  0.39161
C  -4.00662  0.26197  1.50929
H  -4.38330  0.30798  2.53278
C  -2.63154  0.17318  1.30637
H  -1.93700  0.14961  2.15653
C  -7.11906  0.40614 -0.63372
H  -6.90517  1.27850 -1.28015
H  -8.16614  0.47391 -0.30766
H  -7.00997 -0.51068 -1.24358
C  -6.83675  0.44965  1.87940
H  -6.59538 -0.44846  2.47886
H  -7.92997  0.51612  1.79155
H  -6.48277  1.33979  2.43314
B   0.94453  0.01560  2.05953
C   2.34833  0.19273  3.93070
C   0.87574 -0.09912  4.40598
C   3.42260 -0.71237  4.54386
H   4.40693 -0.45918  4.11648
H   3.48141 -0.57254  5.63799
H   3.22690 -1.77485  4.33566
C   2.75772  1.67011  4.07676
H   3.70722  1.83187  3.54130
H   2.00215  2.33720  3.63149
H   2.90400  1.95422  5.13344
C   0.34881  0.82952  5.50556
H   0.93881  0.72015  6.43285
H   0.37762  1.88464  5.19488
H  -0.69787  0.57198  5.74022
C   0.63819 -1.56950  4.79771
H  -0.44480 -1.73890  4.91682
H   1.00420 -2.25156  4.01356
H   1.13342 -1.82689  5.75005
```

6

SCF (BP86) Energy = -1135.28877881
Enthalpy 0K = -1134.771176
Enthalpy 298K = -1134.738368
Free Energy 298K = -1134.834897
Lowest Frequency = 19.5971 cm^{-1}
Second Frequency = 24.2984 cm^{-1}
SCF (BP86-D3BJ) Energy = -1135.42051700
SCF (Toluene) Energy = -1135.29146914
SCF (BS2) Energy = -1135.55687772

```
B  -0.50978  0.04964  0.00927
C   0.11630  1.50704  0.02581
C   0.45873  2.13664  1.25538
C   0.46765  2.15345 -1.19256
C   1.09293  3.39195  1.24706
C   1.10279  3.40835 -1.16231
C   1.41866  4.05251  0.04794
H   1.34495  3.86424  2.20596
H   1.36225  3.89359 -2.11273
C  -2.03341 -0.29759 -0.00600
C  -3.07292  0.70304 -0.01094
C  -2.45758 -1.67579 -0.02112
C  -4.42583  0.31981 -0.03255
C  -3.82088 -2.00857 -0.04201
C  -4.82632 -1.02619 -0.04564
H  -5.19578  1.10154 -0.04018
H  -4.11073 -3.06675 -0.05718
C  -1.47613 -2.83253 -0.01387
H  -0.83662 -2.81610  0.88493
H  -2.01340 -3.79540 -0.02536
H  -0.80602 -2.80924 -0.88968
C  -2.80186  2.19511  0.00478
H  -2.21011  2.52167 -0.86540
H  -3.75263  2.75338  0.00034
H  -2.22806  2.50503  0.89288
C  -6.28906 -1.40752 -0.03568
H  -6.46822 -2.34040 -0.59595
H  -6.64435 -1.57696  0.99801
H  -6.91827 -0.61394 -0.47105
C   0.15226  1.48701 -2.51867
H   0.61171  0.48304 -2.57604
H  -0.93666  1.34855 -2.65658
H   0.52240  2.08235 -3.36998
C   0.14337  1.44686  2.56926
H   0.46166  2.05390  3.43314
H  -0.94009  1.25201  2.67776
H   0.65290  0.46769  2.63240
C   2.06997  5.41979  0.06016
H   2.70986  5.55606  0.94850
H   2.69276  5.57987 -0.83622
H   1.31437  6.22779  0.07850
B   0.82123 -1.03081  0.00038
O   1.45837 -1.46179 -1.16148
O   1.51633 -1.39488  1.14946
C   2.80726 -1.91446 -0.78526
C   2.64110 -2.25534  0.75034
C   3.18001 -3.10122 -1.67921
H   3.22574 -2.77210 -2.73027
H   4.17116 -3.50269 -1.40566
H   2.44129 -3.91362 -1.60947
C   3.75277 -0.72964 -1.04916
H   4.80334 -0.99133 -0.83807
H   3.67595 -0.43968 -2.10956
H   3.47612  0.14543 -0.43922
C   2.20782 -3.70723  1.01559
H   3.02572 -4.41880  0.81194
H   1.91974 -3.80696  2.07485
H   1.33966 -3.98796  0.39791
C   3.84463 -1.89661  1.62854
H   3.61820 -2.13329  2.68121
H   4.73449 -2.47924  1.33318
H   4.08829 -0.82580  1.56568
```

7

SCF (BP86) Energy = -1206.20729573
Enthalpy 0K = -1205.702924
Enthalpy 298K = -1205.670377
Free Energy 298K = -1205.766944
Lowest Frequency = 18.1547 cm^{-1}
Second Frequency = 26.2615 cm^{-1}
SCF (BP86-D3BJ) Energy = -1206.34180149
SCF (Toluene) Energy = -1206.21463164
SCF (BS2) Energy = -1206.50049124

B	0.54805	0.02786	0.72603
C	0.89496	1.55001	0.19602
C	0.46960	2.67977	0.97242
C	1.57326	1.83636	-1.03108
C	0.74365	3.99382	0.54498
C	1.82366	3.16884	-1.42474
C	1.43014	4.26740	-0.64819
H	0.40312	4.83208	1.16793
H	2.34592	3.34764	-2.37419
C	1.47587	-1.23937	0.22858
C	2.78145	-1.30421	0.81994
C	1.12952	-2.29736	-0.66451
C	3.65456	-2.37134	0.53758
C	2.03016	-3.35623	-0.91452
C	3.29691	-3.42381	-0.32011
H	4.64882	-2.37578	1.00449
H	1.72828	-4.15067	-1.61057
C	-0.18082	-2.36674	-1.43904
H	-0.39485	-1.43772	-1.99559
H	-0.13900	-3.18265	-2.17999
H	-1.05597	-2.56195	-0.79458
C	3.29858	-0.23009	1.76427
H	4.38059	-0.35484	1.93836
H	3.12859	0.78324	1.36285
H	2.77975	-0.26889	2.73507
C	4.23617	-4.58198	-0.58257
H	3.96063	-5.12606	-1.50174
H	5.28078	-4.24116	-0.68907
H	4.22059	-5.31271	0.24802
C	2.06824	0.75920	-1.98439
H	2.90127	0.17871	-1.55503
H	1.28801	0.02422	-2.24109
H	2.42074	1.21568	-2.92510
C	-0.29788	2.54854	2.28007
H	0.31249	2.07553	3.06408
H	-0.62025	3.54264	2.63395
H	-1.19771	1.91887	2.17616
C	1.74621	5.68717	-1.06851
H	2.69387	6.03831	-0.61852
H	1.85670	5.77101	-2.16297
H	0.95821	6.39055	-0.74871
C	-3.84830	-0.61859	-0.05655
C	-3.15435	-1.23771	1.02627
C	-1.80026	-1.00042	1.20792
C	-1.71960	0.41745	-0.63455
C	-3.07111	0.23821	-0.89112
H	-3.66756	-1.89332	1.73138
H	-1.23609	-1.43498	2.03551
H	-1.10144	1.08690	-1.23909
H	-3.51648	0.77428	-1.73040
N	-1.07261	-0.19817	0.38667
N	-5.18499	-0.82749	-0.27509
C	-5.94645	-1.69978	0.61835
H	-5.94893	-1.31921	1.65705
H	-6.98741	-1.74892	0.27107
H	-5.53925	-2.72785	0.62479
C	-5.85854	-0.15932	-1.38811
H	-5.40514	-0.43342	-2.35870
H	-6.91291	-0.46684	-1.40676
H	-5.82382	0.94163	-1.28569
F	0.51640	-0.04477	2.16284

8

SCF (BP86) Energy = -2680.29874431
Enthalpy 0K = -2679.053198
Enthalpy 298K = -2678.975319
Free Energy 298K = -2679.166320
Lowest Frequency = 14.6761 cm^{-1}
Second Frequency = 14.9174 cm^{-1}
SCF (BP86-D3BJ) Energy = -2680.69166038
SCF (Toluene) Energy = -2680.30644638
SCF (BS2) Energy = -3079.44741577

C	2.31582	5.26189	-0.80933
H	2.62544	5.11978	-1.85810
H	3.22017	5.10316	-0.19779
H	1.98279	6.30166	-0.67991
C	1.21352	4.28332	-0.42501
C	0.00060	4.87060	-0.00006
H	0.00069	5.96252	-0.00013
C	-1.21242	4.28357	0.42501
C	-2.31459	5.26235	0.80915
H	-1.98107	6.30206	0.68056
H	-2.62509	5.11970	1.85757
H	-3.21855	5.10434	0.19680
C	-2.75214	2.56347	0.97181
C	-3.80166	2.38757	0.02272
C	-5.06379	1.97057	0.48576
H	-5.87686	1.82419	-0.23218
C	-5.30586	1.76539	1.85070
H	-6.29824	1.45186	2.19247
C	-4.27863	1.98365	2.77616
H	-4.47648	1.84346	3.84491
C	-2.99273	2.38747	2.36406
C	-1.91302	2.65183	3.41712
H	-1.04723	3.09822	2.89720
C	-2.37996	3.65750	4.49432
H	-2.72916	4.60668	4.05280
H	-1.55005	3.88971	5.18436
H	-3.20755	3.24936	5.10086
C	-1.43299	1.33950	4.07560
H	-2.26421	0.83140	4.59636
H	-0.64474	1.54564	4.82143
H	-1.02433	0.64952	3.32154
C	-3.57084	2.68403	-1.46164
H	-2.74965	3.42053	-1.52196
C	-4.79792	3.31244	-2.15511
H	-5.63045	2.59383	-2.25366
H	-4.52878	3.63532	-3.17561
H	-5.17502	4.19241	-1.60619
C	-3.11366	1.42356	-2.22554
H	-2.19650	0.98252	-1.80051
H	-2.90628	1.66290	-3.28379
H	-3.89131	0.64271	-2.19658
C	2.75305	2.56294	-0.97154
C	2.99384	2.38719	-2.36379
C	4.27966	1.98297	-2.77573
H	4.47767	1.84291	-3.84446
C	5.30664	1.76410	-1.85013
H	6.29895	1.45024	-2.19178
C	5.06441	1.96912	-0.48519
H	5.87728	1.82231	0.23287
C	3.80234	2.38649	-0.02230
C	3.57141	2.68285	1.46206
H	2.75028	3.41941	1.52235
C	4.79849	3.31110	2.15568
H	5.63097	2.59243	2.25420
H	4.52931	3.63389	3.17620
H	5.17568	4.19110	1.60688
C	3.11410	1.42234	2.22583
H	2.19693	0.98140	1.80073

```
H   2.90667  1.66161  3.28410
H   3.89169  0.64144  2.19686
C   1.91438  2.65210 -3.41696
H   1.04893  3.09933 -2.89721
C   2.38212  3.65705 -4.49449
H   2.73233  4.60600 -4.05326
H   1.55231  3.88987 -5.18444
H   3.20920  3.24797 -5.10108
C   1.43330  1.33995 -4.07503
H   2.26414  0.83102 -4.59559
H   0.64524  1.54648 -4.82095
H   1.02409  0.65050 -3.32078
C   2.31432 -5.26246  0.80912
H   2.62424 -5.12034  1.85780
H   3.21856 -5.10402  0.19733
H   1.98096 -6.30214  0.67985
C   1.21220 -4.28361  0.42498
C  -0.00088 -4.87056  0.00003
H  -0.00105 -5.96249 -0.00000
C  -1.21374 -4.28322 -0.42502
C  -2.31604 -5.26173 -0.80952
H  -2.62538 -5.11965 -1.85837
H  -3.22053 -5.10289 -0.19822
H  -1.98311 -6.30151 -0.67995
C  -2.75310 -2.56278 -0.97170
C  -3.80256 -2.38645 -0.02261
C  -5.06459 -1.96919 -0.48569
H  -5.87762 -1.82252  0.23222
C  -5.30661 -1.76412 -1.85067
H  -6.29890 -1.45037 -2.19247
C  -4.27945 -1.98280 -2.77610
H  -4.47729 -1.84271 -3.84487
C  -2.99367 -2.38694 -2.36396
C  -1.91404 -2.65173 -3.41700
H  -1.04856 -3.09877 -2.89711
C  -2.38147 -3.65685 -4.49450
H  -2.73151 -4.60586 -4.05326
H  -1.55156 -3.88954 -5.18438
H  -3.20859 -3.24798 -5.10117
C  -1.43313 -1.33955 -4.07512
H  -2.26400 -0.83081 -4.59579
H  -0.64495 -1.54599 -4.82095
H  -1.02408 -0.64999 -3.32087
C  -3.57178 -2.68292  1.46176
H  -2.75128 -3.42020  1.52201
C  -4.79932 -3.31008  2.15555
H  -5.63115 -2.59066  2.25416
H  -4.53028 -3.63307  3.17603
H  -5.17736 -4.18977  1.60682
C  -3.11333 -1.42283  2.22551
H  -2.19595 -0.98247  1.80026
H  -2.90582 -1.66235  3.28370
H  -3.89034 -0.64134  2.19680
C   2.75219 -2.56366  0.97148
C   2.99320 -2.38808  2.36371
C   4.27919 -1.98428  2.77553
H   4.47738 -1.84437  3.84424
C   5.30612 -1.76566  1.84981
H   6.29857 -1.45214  2.19138
C   5.06366 -1.97049  0.48489
H   5.87650 -1.82389 -0.23326
C   3.80141 -2.38746  0.02212
C   3.57022 -2.68372 -1.46222
H   2.74906 -3.42026 -1.52240
C   4.79718 -3.31202 -2.15603
H   5.62966 -2.59337 -2.25467
H   4.52785 -3.63481 -3.17650
H   5.17442 -4.19202 -1.60725
C   3.11283 -1.42320 -2.22589
H   2.19573 -0.98224 -1.80066
H   2.90526 -1.66245 -3.28413
H   3.89043 -0.64230 -2.19701
C   1.91379 -2.65275  3.41699
H   1.04820 -3.09984  2.89733
C   2.38142 -3.65774  4.49453
H   2.73146 -4.60677  4.05332
H   1.55161 -3.89041  5.18453
H   3.20859 -3.24877  5.10107
C   1.43303 -1.34048  4.07504
H   2.26402 -0.83172  4.59553
H   0.64497 -1.54681  4.82103
H   1.02391 -0.65096  3.32081
F   0.00001  0.00003  1.22828
Mg  0.00032  1.51512  0.00011
Mg -0.00040 -1.51505  0.00014
N   1.44156  2.95233 -0.50495
N  -1.44062  2.95262  0.50511
N   1.44053 -2.95267  0.50500
N  -1.44170 -2.95221 -0.50491
F  -0.00022  0.00004 -1.22803
```

9

SCF (BP86) Energy = -3138.14432691
Enthalpy 0K = -3136.730376
Enthalpy 298K = -3136.640129
Free Energy 298K = -3136.861424
Lowest Frequency = 8.6375 cm^{-1}
Second Frequency = 13.6873 cm^{-1}
SCF (BP86-D3BJ) Energy = -3138.59580646
SCF (Toluene) Energy = -3138.15566091
SCF (BS2) Energy = -3537.40568351

```
B  -0.01955  1.72697  0.01651
C  -4.27592 -1.49165  3.74470
H  -5.09884 -0.75600  3.78459
H  -3.55412 -1.19698  4.52267
H  -4.69402 -2.47861  3.99401
C  -3.63303 -1.50716  2.36334
C  -4.02918 -2.56981  1.51450
H  -4.67999 -3.30949  1.98770
C  -3.87659 -2.73433  0.11376
C  -4.76244 -3.80907 -0.50659
H  -5.10859 -4.52207  0.25660
H  -4.25354 -4.35896 -1.31162
H  -5.65591 -3.34456 -0.96155
C  -3.09819 -2.23634 -2.08783
C  -3.99069 -1.50337 -2.92539
C  -3.99771 -1.76590 -4.30950
H  -4.68705 -1.20821 -4.95408
C  -3.14624 -2.72134 -4.87587
H  -3.17157 -2.91708 -5.95327
C  -2.25988 -3.42262 -4.04957
H  -1.59065 -4.17044 -4.49032
C  -2.21200 -3.20073 -2.65862
C  -1.23171 -4.02074 -1.81407
H  -1.28459 -3.64165 -0.78045
C  -1.59844 -5.52385 -1.80042
H  -2.61209 -5.70330 -1.40592
H  -0.88799 -6.08615 -1.16881
H  -1.55364 -5.95508 -2.81658
C   0.22498 -3.84445 -2.29406
H   0.36654 -4.21980 -3.32320
H   0.91672 -4.40270 -1.63969
H   0.52475 -2.78582 -2.27170
C  -4.93558 -0.43385 -2.37249
H  -4.77610 -0.38326 -1.28107
C  -6.41991 -0.78791 -2.61981
H  -6.65091 -0.82176 -3.69945
H  -7.08083 -0.03092 -2.16183
H  -6.68333 -1.77078 -2.19404
C  -4.60922  0.95698 -2.96307
H  -3.57497  1.25738 -2.72894
```

H -5.29163 1.72294 -2.55465
H -4.72206 0.96156 -4.06174
C -2.74043 0.65361 2.83945
C -3.75784 1.64851 2.71552
C -3.66934 2.81581 3.49678
H -4.44536 3.58377 3.39794
C -2.61691 3.01292 4.39931
H -2.56959 3.92473 5.00497
C -1.63185 2.02831 4.52384
H -0.81141 2.17159 5.23643
C -1.67042 0.84495 3.75883
C -0.57594 -0.20074 3.96073
H -0.75403 -1.01067 3.23167
C -0.63316 -0.82921 5.37263
H -0.46231 -0.06750 6.15451
H   0.14986 -1.60024 5.47624
H -1.60927 -1.30203 5.57587
C   0.82802 0.38251 3.69457
H   0.90540 0.80669 2.67842
H   1.59412 -0.40261 3.80513
H   1.06978 1.18313 4.41750
C -4.91923 1.50050 1.72963
H -4.92846 0.45676 1.37242
C -6.29470 1.78513 2.37156
H -6.46491 1.16282 3.26681
H -7.10454 1.57412 1.65130
H -6.39650 2.84145 2.67779
C -4.69254 2.40222 0.49561
H -4.63992 3.46564 0.79282
H -5.51813 2.29378 -0.23001
H -3.74666 2.13426 -0.00474
C   4.69041 -3.85181 0.39135
H   4.04523 -4.65633 0.78170
H   5.29815 -3.50335 1.24278
H   5.35925 -4.28225 -0.36797
C   3.85877 -2.71387 -0.18881
C   3.99761 -2.51326 -1.58591
H   4.63437 -3.24677 -2.08656
C   3.59661 -1.42859 -2.40495
C   4.23282 -1.37735 -3.78840
H   5.06270 -0.64879 -3.80947
H   3.51069 -1.05277 -4.55389
H   4.64102 -2.36006 -4.06902
C   2.70702 0.74709 -2.81505
C   1.63664 0.96756 -3.72790
C   1.59868 2.17332 -4.45705
H   0.77764 2.33838 -5.16418
C   2.58501 3.15268 -4.30486
H   2.53818 4.08212 -4.88314
C   3.63813 2.92682 -3.41028
H   4.41594 3.68992 -3.28965
C   3.72572 1.73678 -2.66380
C   4.89083 1.56072 -1.68716
H   4.88868 0.51290 -1.34204
C   6.26529 1.83471 -2.33647
H   6.42121 1.21933 -3.23901
H   7.07785 1.60653 -1.62459
H   6.37719 2.89264 -2.63345
C   4.68623 2.45239 -0.44212
H   4.63888 3.51926 -0.72727
H   5.51975 2.32988 0.27209
H   3.74472 2.18697 0.06774
C   0.54182 -0.07019 -3.96451
H   0.71632 -0.90071 -3.25822
C   0.60445 -0.65734 -5.39384
H   0.43340 0.12662 -6.15336
H -0.17591 -1.42736 -5.52106
H   1.58231 -1.12131 -5.60859
C -0.86320 0.50394 -3.68619
H -0.94431 0.89440 -2.65703
H -1.62763 -0.27857 -3.82428
H -1.10332 1.32695 -4.38395
C   3.15061 -2.23843 2.04114
C   2.35519 -3.26751 2.63085
C   2.44414 -3.47953 4.02139
H   1.83372 -4.26755 4.47712
C   3.29980 -2.72092 4.82829
H   3.36258 -2.91400 5.90460
C   4.07245 -1.71153 4.24312
H   4.74157 -1.11256 4.87130
C   4.00731 -1.44374 2.86173
C   4.87144 -0.32003 2.28467
H   4.57264 -0.18371 1.23035
C   6.37205 -0.69329 2.30121
H   6.72903 -0.85966 3.33339
H   6.98055 0.11710 1.86164
H   6.56926 -1.61344 1.72613
C   4.63844 1.01798 3.02078
H   3.57800 1.31595 2.98119
H   5.23952 1.82118 2.56030
H   4.93289 0.95558 4.08330
C   1.40289 -4.14909 1.81794
H   1.53794 -3.89509 0.75266
C   1.69427 -5.65733 1.99940
H   2.74402 -5.91424 1.77709
H   1.04999 -6.25377 1.32996
H   1.48741 -5.98679 3.03304
C -0.06985 -3.85446 2.17984
H -0.26765 -4.05432 3.24784
H -0.75210 -4.48745 1.58573
H -0.31509 -2.80381 1.96713
C -1.15011 4.03598 -0.27895
H -2.00847 3.40465 -0.51798
C -1.19457 5.41997 -0.28229
H -2.13338 5.91065 -0.54251
C -0.02865 6.17228 0.05519
C   1.14038 5.41861 0.37857
H   2.07737 5.90820 0.64726
C   1.10133 4.03475 0.35026
H   1.96202 3.40226 0.57764
C -1.25082 8.27656 -0.26903
H -1.58700 8.05543 -1.29907
H -1.05118 9.35447 -0.19992
H -2.07576 8.03525 0.42673
C   1.18483 8.27513 0.41894
H   1.52194 8.03584 1.44461
H   0.98066 9.35331 0.37032
H   2.01076 8.05055 -0.28122
F -0.00682 -1.44582 -0.00799
Mg -1.71501 -0.61375 0.19401
Mg 1.69497 -0.60074 -0.19888
N -2.79348 -0.51678 1.99967
N -3.07563 -1.98121 -0.66867
N   3.08274 -1.96676 0.62586
N   2.76026 -0.44803 -2.00981
N -0.02311 3.34160 0.02970
N -0.03146 7.54174 0.06797
O -1.23348 1.21931 -0.24819
O   1.19595 1.22039 0.27313

10

SCF (BP86) Energy = -2475.41345189
Enthalpy 0K = -2474.272494
Enthalpy 298K = -2474.201030
Free Energy 298K = -2474.378556
Lowest Frequency = 14.6049 cm^{-1}
Second Frequency = 18.2232 cm^{-1}
SCF (BP86-D3BJ) Energy = -2475.77331419
SCF (Toluene) Energy = -2475.42094070
SCF (BS2) Energy = -2675.25254290

Mg 0.13605 -0.94724 0.34036

O	0.10969	2.60506	2.53930
O	0.40765	0.41291	1.90452
N	1.59684	-2.40983	0.06567
N	-1.54441	-2.22516	0.43711
C	2.38313	-4.70789	0.61993
H	2.78539	-4.93794	-0.38077
H	2.00110	-5.63806	1.06471
H	3.23564	-4.35263	1.22087
C	1.29321	-3.64611	0.54114
C	0.00291	-4.07425	0.92765
H	-0.03123	-5.11307	1.26811
C	-1.29082	-3.52268	0.69225
C	-2.39360	-4.57715	0.68386
H	-3.37856	-4.16275	0.43544
H	-2.45207	-5.07391	1.66739
H	-2.14572	-5.36064	-0.05188
C	-2.90356	-1.81871	0.11721
C	-3.21700	-1.50439	-1.24093
C	-4.51680	-1.07177	-1.56212
H	-4.75266	-0.82433	-2.60081
C	-5.51182	-0.95811	-0.58725
H	-6.51823	-0.62272	-0.85825
C	-5.20527	-1.28050	0.73579
H	-5.98434	-1.20003	1.50180
C	-3.91690	-1.70577	1.12100
C	-3.70810	-2.02383	2.60629
H	-2.63859	-2.25390	2.75896
C	-4.53541	-3.24676	3.07555
H	-5.61622	-3.04657	2.97121
H	-4.33883	-3.45518	4.14219
H	-4.31133	-4.15922	2.50412
C	-4.08629	-0.81753	3.49961
H	-5.17981	-0.66695	3.51338
H	-3.63353	0.11918	3.14192
H	-3.76765	-0.98930	4.54268
C	-2.21455	-1.72175	-2.37483
H	-1.19681	-1.60708	-1.95898
C	-2.33417	-3.16957	-2.90938
H	-3.34986	-3.34937	-3.30410
H	-2.14220	-3.91554	-2.12087
H	-1.61615	-3.34655	-3.72920
C	-2.35338	-0.70699	-3.52669
H	-3.27485	-0.87428	-4.11229
H	-1.50728	-0.81656	-4.22531
H	-2.36328	0.33028	-3.15549
C	2.83808	-2.30208	-0.68258
C	2.81607	-2.58543	-2.08301
C	4.01593	-2.50877	-2.81627
H	3.99882	-2.73567	-3.88801
C	5.22498	-2.15515	-2.20932
H	6.14770	-2.10431	-2.79677
C	5.23690	-1.86299	-0.84305
H	6.17961	-1.57838	-0.36218
C	4.06610	-1.92944	-0.06152
C	1.54037	-2.98946	-2.82579
H	0.69333	-2.87815	-2.12670
C	1.58550	-4.47144	-3.26688
H	2.42495	-4.65428	-3.96093
H	0.65217	-4.75034	-3.78672
H	1.70729	-5.15039	-2.40647
C	1.27785	-2.07365	-4.04189
H	1.20762	-1.01518	-3.74160
H	0.33370	-2.35429	-4.53984
H	2.08020	-2.15559	-4.79560
C	4.17556	-1.57866	1.42132
H	3.17506	-1.72203	1.86386
C	4.58906	-0.09925	1.60286
H	4.65984	0.16464	2.67348
H	3.87861	0.58968	1.11736
H	5.58144	0.08864	1.15751
C	5.16934	-2.48918	2.18018
H	4.92447	-3.55909	2.07250
H	5.16723	-2.24809	3.25783
H	6.20102	-2.34956	1.81277
C	0.52001	0.43112	3.40113
C	1.64174	-0.50338	3.84227
H	1.75084	-0.45862	4.93981
H	2.60414	-0.23462	3.38888
H	1.40183	-1.54713	3.57675
C	-0.82203	-0.05320	3.94893
H	-1.04082	-1.05949	3.55706
H	-1.63829	0.62030	3.65182
H	-0.80163	-0.11447	5.04944
C	0.78080	1.96118	3.66780
C	2.26330	2.36306	3.58976
H	2.73336	1.99865	2.66332
H	2.83475	1.98410	4.45393
H	2.32886	3.46233	3.58454
C	0.15845	2.49414	4.96346
H	0.59311	1.99255	5.84530
H	-0.93305	2.35936	4.98150
H	0.36860	3.57225	5.04927
B	-0.12567	2.07399	-0.23301
B	0.05180	1.74740	1.45987
F	0.10355	0.55676	-0.82469
C	-1.52868	2.53114	-0.93063
C	-1.56917	2.84769	-2.32772
C	-2.76545	2.62572	-0.22369
C	-2.76727	3.26489	-2.94110
C	-3.94555	3.05016	-0.86795
C	-3.97056	3.39004	-2.22802
H	-2.75576	3.49907	-4.01417
H	-4.87449	3.11092	-0.28571
C	1.18596	3.02063	-0.54294
C	1.10182	4.40323	-0.16193
C	2.43603	2.59046	-1.08992
C	2.20236	5.26737	-0.31665
C	3.51522	3.49080	-1.22469
C	3.43104	4.83534	-0.83930
H	2.08740	6.31869	-0.01971
H	4.45313	3.11962	-1.65957
C	4.61217	5.77426	-0.96483
H	5.34748	5.40234	-1.69826
H	4.29652	6.78341	-1.28208
H	5.14328	5.89265	-0.00118
C	-5.23525	3.88563	-2.89675
H	-6.13762	3.52907	-2.37172
H	-5.27977	4.99108	-2.90619
H	-5.29655	3.55296	-3.94724
C	-2.88714	2.28792	1.25066
H	-2.48976	1.27970	1.46747
H	-2.33965	2.99838	1.89307
H	-3.94449	2.29348	1.56505
C	-0.34610	2.74274	-3.22075
H	0.40779	3.50945	-2.97686
H	0.14910	1.76474	-3.10973
H	-0.62690	2.86316	-4.28078
C	2.68697	1.18527	-1.60797
H	2.57013	0.41104	-0.83327
H	1.99736	0.91523	-2.42464
H	3.71495	1.09313	-1.99539
C	-0.16866	5.01778	0.40020
H	-0.99481	4.98905	-0.32923
H	-0.51446	4.48772	1.30283
H	0.00441	6.07137	0.67868

11

SCF (BP86) Energy = -1797.96717001
Enthalpy 0K = -1797.175579
Enthalpy 298K = -1797.124941
Free Energy 298K = -1797.261380
Lowest Frequency = 10.2836 cm^{-1}

Second Frequency = 12.2461 cm^{-1}
SCF (BP86-D3BJ) Energy = -1798.18500959
SCF (Toluene) Energy = -1797.97780611
SCF (BS2) Energy = -1997.65717121

Mg	0.68787	0.00017	-0.00066
O	-0.95403	-0.00040	1.19653
O	-0.95444	-0.00037	-1.19734
N	2.09924	1.51053	-0.00075
N	2.10033	-1.50922	-0.00064
N	-3.14777	-0.00089	-0.00001
N	-7.33708	-0.00173	0.00085
C	4.37879	2.47755	-0.00011
H	4.20477	3.11937	-0.88084
H	5.42934	2.15214	-0.00317
H	4.20879	3.11484	0.88474
C	3.42788	1.28954	-0.00077
C	4.02155	0.00139	-0.00106
H	5.11421	0.00179	-0.00125
C	3.42882	-1.28720	-0.00084
C	4.38066	-2.47448	-0.00095
H	4.20924	-3.11397	-0.88391
H	4.20903	-3.11436	0.88168
H	5.43095	-2.14825	-0.00077
C	1.59461	2.85901	-0.00020
C	1.29725	3.50244	-1.23733
C	1.57680	2.83341	-2.58509
H	2.13852	1.90457	-2.38284
C	0.25858	2.43495	-3.28987
H	-0.32684	1.73246	-2.67118
H	0.47279	1.94836	-4.25881
H	-0.36173	3.32706	-3.49320
C	2.45072	3.71540	-3.50497
H	2.69583	3.17408	-4.43569
H	3.39897	4.00328	-3.01871
H	1.92937	4.64569	-3.79272
C	0.70647	4.78058	-1.20845
H	0.47125	5.27971	-2.15562
C	0.41149	5.42153	0.00075
H	-0.04797	6.41612	0.00113
C	0.70734	4.78006	1.20946
H	0.47275	5.27879	2.15700
C	1.29817	3.50193	1.23740
C	1.57855	2.83234	2.58469
H	2.14036	1.90370	2.38176
C	2.45268	3.71410	3.50459
H	1.93119	4.64407	3.79313
H	3.40056	4.00259	3.01796
H	2.69847	3.17235	4.43487
C	0.26073	2.43327	3.28993
H	0.47552	1.94621	4.25851
H	-0.32489	1.73104	2.67114
H	-0.35960	3.32518	3.49403
C	1.59683	-2.85811	-0.00015
C	1.30005	-3.50180	-1.23728
C	1.57904	-2.83255	-2.58504
H	2.14019	-1.90336	-2.38282
C	0.26048	-2.43491	-3.28967
H	0.47429	-1.94815	-4.25862
H	-0.32535	-1.73283	-2.67092
H	-0.35927	-3.32741	-3.49296
C	2.45336	-3.71398	-3.50506
H	1.93250	-4.64455	-3.79284
H	3.40180	-4.00140	-3.01889
H	2.69809	-3.17246	-4.43575
C	0.71036	-4.78044	-1.20842
H	0.47560	-5.27977	-2.15560
C	0.41591	-5.42166	0.00077
H	-0.04269	-6.41664	0.00114
C	0.71118	-4.77994	1.20949
H	0.47702	-5.27888	2.15703
C	1.30088	-3.50129	1.23744
C	1.58073	-2.83148	2.58474
H	2.14165	-1.90230	2.38181
C	2.45579	-3.71248	3.50450
H	2.70109	-3.17060	4.43483
H	3.40394	-3.99997	3.01781
H	1.93522	-4.64299	3.79296
C	0.26262	-2.43370	3.29013
H	-0.32370	-1.73192	2.67149
H	0.47705	-1.94656	4.25874
H	-0.35690	-3.32618	3.49418
C	-3.83338	-0.00107	1.17431
H	-3.18956	-0.00097	2.05869
C	-5.21699	-0.00140	1.21697
H	-5.70998	-0.00157	2.18993
C	-5.96842	-0.00151	0.00057
C	-5.21749	-0.00139	-1.21613
H	-5.71089	-0.00160	-2.18889
C	-3.83386	-0.00107	-1.17404
H	-3.19043	-0.00096	-2.05870
C	-8.07207	-0.00244	1.26661
H	-7.84069	-0.89979	1.86974
H	-9.14990	-0.00240	1.05563
H	-7.84083	0.89436	1.87058
C	-8.07258	-0.00216	-1.26463
H	-7.84120	0.89456	-1.86866
H	-9.15032	-0.00166	-1.05321
H	-7.84180	-0.89959	-1.86787
B	-1.56936	-0.00052	-0.00031

12

SCF (BP86) Energy = -1340.11511159
Enthalpy 0K = -1339.493749
Enthalpy 298K = -1339.454560
Free Energy 298K = -1339.564987
Lowest Frequency = 17.9868 cm^{-1}
Second Frequency = 19.4182 cm^{-1}
SCF (BP86-D3BJ) Energy = -1340.28529149
SCF (Toluene) Energy = -1340.12512879
SCF (BS2) Energy = -1539.69436983

Mg	-0.00002	-0.29588	-0.73849
N	1.50871	0.07114	0.55599
N	-1.50872	0.07115	0.55602
C	2.48004	0.40525	2.80787
H	3.17361	-0.44405	2.68628
H	2.16165	0.45943	3.85889
H	3.05906	1.31454	2.57045
C	1.28965	0.26336	1.87400
C	0.00002	0.33749	2.46079
H	0.00003	0.48463	3.54329
C	-1.28963	0.26335	1.87403
C	-2.48000	0.40521	2.80795
H	-2.16157	0.45935	3.85895
H	-3.17356	-0.44410	2.68635
H	-3.05903	1.31449	2.57058
C	2.85434	0.02710	0.03880
C	3.46417	-1.23670	-0.20359
C	2.75219	-2.54998	0.12913
H	1.87719	-2.30059	0.75757
C	2.22986	-3.23323	-1.15943
H	1.52412	-2.59021	-1.71624
H	1.71490	-4.17871	-0.91139
H	3.06812	-3.47571	-1.83655
C	3.63872	-3.51476	0.94651
H	3.06166	-4.40958	1.23746
H	4.01987	-3.03901	1.86636
H	4.50946	-3.86278	0.36374
C	4.73879	-1.26216	-0.80217
H	5.21723	-2.22803	-0.99782
C	5.40091	-0.08099	-1.15655

H 6.39177 -0.12304 -1.62096
C 4.78475 1.15471 -0.92324
H 5.29936 2.07668 -1.21564
C 3.51057 1.23626 -0.33008
C 2.83249 2.59777 -0.16165
H 1.96315 2.45972 0.50480
C 3.75091 3.65671 0.48492
H 4.60860 3.90813 -0.16311
H 4.15461 3.31014 1.45157
H 3.19174 4.59144 0.66397
C 2.29466 3.10026 -1.52275
H 1.75649 4.05754 -1.40719
H 1.60299 2.37064 -1.97989
H 3.12025 3.25666 -2.23891
C -2.85437 0.02709 0.03886
C -3.46416 -1.23672 -0.20350
C -2.75213 -2.54997 0.12923
H -1.87712 -2.30053 0.75765
C -2.22980 -3.23322 -1.15933
H -1.71476 -4.17866 -0.91127
H -1.52413 -2.59017 -1.71618
H -3.06807 -3.47579 -1.83641
C -3.63860 -3.51477 0.94666
H -4.50933 -3.86287 0.36390
H -4.01976 -3.03900 1.86649
H -3.06148 -4.40955 1.23763
C -4.73877 -1.26223 -0.80210
H -5.21719 -2.22812 -0.99774
C -5.40091 -0.08109 -1.15653
H -6.39176 -0.12317 -1.62095
C -4.78478 1.15464 -0.92324
H -5.29940 2.07659 -1.21569
C -3.51061 1.23622 -0.33008
C -2.83255 2.59776 -0.16174
H -1.96325 2.45981 0.50476
C -3.75105 3.65676 0.48465
H -3.19192 4.59152 0.66360
H -4.15478 3.31029 1.45132
H -4.60872 3.90806 -0.16346
C -2.29466 3.10012 -1.52288
H -1.60294 2.37048 -1.97989
H -1.75653 4.05743 -1.40738
H -3.12021 3.25642 -2.23910
F -0.00004 -1.00237 -2.35991

13

SCF (BP86) Energy = -2192.16490856
Enthalpy 0K = -2191.080967
Enthalpy 298K = -2191.014802
Free Energy 298K = -2191.180514
Lowest Frequency = 16.8569 cm^{-1}
Second Frequency = 17.2582 cm^{-1}
SCF (BP86-D3BJ) Energy = -2192.48302057
SCF (Toluene) Energy = -2192.17006205
SCF (BS2) Energy = -2391.93281071

Mg -0.09594 -0.02915 0.05145
O 2.39743 -1.06662 -1.98148
O 1.85422 1.12113 -2.41660
N -0.95960 -1.79883 0.93107
N 0.82216 0.51443 1.90546
N -2.99417 1.78633 0.42041
N -2.26430 1.88271 -1.61834
C -1.97354 -3.00545 2.87095
H -3.01425 -2.68978 3.06665
H -1.53799 -3.30599 3.83716
H -2.00995 -3.88019 2.20555
C -1.18247 -1.85421 2.25319
C -0.73234 -0.87503 3.18811
H -1.08605 -1.04042 4.21066
C 0.26855 0.12606 3.07002
C 0.73842 0.74853 4.38136
H 1.67648 0.28304 4.72888
H -0.01424 0.61065 5.17331
H 0.94537 1.82417 4.25943
C 2.06800 1.23248 1.94386
C 3.28056 0.54121 2.26055
C 4.49491 1.25412 2.25691
H 5.42204 0.72224 2.50020
C 4.54833 2.61660 1.94172
H 5.50384 3.15197 1.94928
C 3.36449 3.28060 1.60668
H 3.39776 4.34551 1.34848
C 2.12329 2.61463 1.59687
C 0.87479 3.41023 1.22257
H 0.02138 2.70862 1.24779
C 0.59821 4.54252 2.23838
H 0.47835 4.15210 3.26377
H -0.31970 5.09730 1.97189
H 1.42871 5.27016 2.25741
C 0.97179 3.97692 -0.21135
H 1.78341 4.72247 -0.29179
H 0.03005 4.48550 -0.48740
H 1.17423 3.17792 -0.94420
C 3.32752 -0.96409 2.53632
H 2.29100 -1.33922 2.57292
C 4.04793 -1.69501 1.37929
H 3.55508 -1.50314 0.41174
H 4.05650 -2.78561 1.55595
H 5.09912 -1.36326 1.29974
C 4.00362 -1.30658 3.88367
H 5.07134 -1.02395 3.88552
H 3.95101 -2.39292 4.07377
H 3.52522 -0.79109 4.73399
C -1.33765 -2.89115 0.06986
C -0.33046 -3.81328 -0.35648
C -0.67451 -4.81264 -1.28607
H 0.09333 -5.51968 -1.61474
C -1.97165 -4.92416 -1.80079
H -2.21348 -5.70639 -2.52840
C -2.95543 -4.03237 -1.36611
H -3.97740 -4.12528 -1.75220
C -2.66841 -3.01697 -0.43162
C -3.81350 -2.11518 0.02976
H -3.39463 -1.40962 0.76694
C -4.40574 -1.28418 -1.12890
H -3.64177 -0.63614 -1.58542
H -5.22761 -0.63877 -0.76933
H -4.81865 -1.93608 -1.91890
C -4.93544 -2.92892 0.71807
H -5.43188 -3.60606 0.00081
H -5.71225 -2.25762 1.12725
H -4.54781 -3.54824 1.54301
C 1.08171 -3.77405 0.23008
H 1.28817 -2.72650 0.51520
C 2.17493 -4.20826 -0.76487
H 2.08782 -5.27435 -1.04184
H 3.16860 -4.07621 -0.30473
H 2.14367 -3.59023 -1.67500
C 1.15643 -4.63400 1.51545
H 0.44048 -4.29406 2.28108
H 2.16906 -4.58833 1.95442
H 0.93241 -5.69227 1.28947
C -1.96303 1.34882 -0.38449
C -3.03310 1.48649 1.87657
H -2.22959 0.74425 2.01909
C -4.35306 0.83844 2.33151
H -5.16555 1.57317 2.45071
H -4.18994 0.36658 3.31473
H -4.68608 0.05667 1.63189
C -2.68875 2.71920 2.72877
H -2.57587 2.40957 3.78124

```
H  -3.47947  3.48767  2.69187
H  -1.74281  3.17573  2.40454
C  -5.15214  3.20067  0.27401
H  -5.92265  2.45475  0.53560
H  -5.59408  3.88234 -0.46909
H  -4.94801  3.79812  1.17812
C  -3.91325  2.57230 -0.29082
C  -3.45187  2.62688 -1.59063
C  -4.06270  3.33783 -2.76093
H  -3.48482  4.22931 -3.06040
H  -5.07971  3.67350 -2.50530
H  -4.14897  2.68714 -3.64666
C  -1.39775  1.63999 -2.80484
H  -0.49557  1.17063 -2.37559
C  -0.94510  2.93681 -3.49334
H  -1.71793  3.37211 -4.14802
H  -0.06540  2.70509 -4.11462
H  -0.63517  3.69072 -2.75271
C  -2.02214  0.63169 -3.78247
H  -2.23545 -0.32654 -3.28253
H  -1.30629  0.43346 -4.59787
H  -2.95475  1.00192 -4.24177
C   3.22380 -0.67467 -3.13266
C   3.14387  0.89508 -3.08660
C   4.22305  1.53823 -2.19680
H   3.98242  2.60417 -2.05451
H   5.22560  1.46832 -2.65381
H   4.25198  1.06790 -1.20119
C   3.11209  1.58689 -4.45405
H   2.26867  1.23821 -5.06967
H   4.04791  1.40456 -5.01177
H   3.00952  2.67645 -4.31684
C   2.55928 -1.28554 -4.38074
H   2.46820 -2.37518 -4.24226
H   3.15080 -1.10168 -5.29458
H   1.54561 -0.87861 -4.52785
C   4.62742 -1.26080 -2.94686
H   5.07341 -0.94888 -1.99092
H   5.29817 -0.94798 -3.76693
H   4.57479 -2.36214 -2.95401
B   1.51799 -0.02010 -1.65376
```

14

SCF (BP86) Energy = -1364.65635576
Enthalpy 0K = -1364.021080
Enthalpy 298K = -1363.981923
Free Energy 298K = -1364.089382
Lowest Frequency = 26.4167 cm^{-1}
Second Frequency = 32.8225 cm^{-1}
SCF (BP86-D3BJ) Energy = -1364.83144431
SCF (Toluene) Energy = -1364.66089079
SCF (BS2) Energy = -1364.97993536

```
B   0.05273  0.39497 -0.55073
C   1.66500  0.75906 -0.37495
C   2.59949  0.23703 -1.33219
C   2.20791  1.61598  0.63698
C   3.96638  0.57683 -1.27291
C   3.58331  1.93158  0.65951
C   4.48530  1.43533 -0.29230
H   4.64759  0.15200 -2.02292
H   3.95771  2.59139  1.45374
C  -1.04241  1.48425  0.06285
C  -1.20756  2.71573 -0.65523
C  -1.87536  1.30444  1.20976
C  -2.17663  3.65892 -0.25967
C  -2.83932  2.27022  1.57066
C  -3.02319  3.45291  0.84108
H  -2.26351  4.59297 -0.83133
H  -3.45489  2.09491  2.46361
C  -1.75582  0.11507  2.15357
H  -0.70780 -0.08432  2.43317
H  -2.30913  0.31137  3.08768
H  -2.15731 -0.82411  1.73271
C  -0.34045  3.09465 -1.84621
H  -0.45480  4.16729 -2.07796
H   0.72577  2.89404 -1.65167
H  -0.60337  2.51105 -2.74204
C  -4.08427  4.46351  1.22195
H  -4.37258  4.36802  2.28249
H  -3.73739  5.49787  1.05498
H  -5.00385  4.33229  0.62066
C   1.37278  2.23101  1.74924
H   0.68791  3.00774  1.37195
H   0.74032  1.49046  2.26534
H   2.02827  2.69431  2.50640
C   2.20278 -0.70274 -2.46342
H   1.67769 -0.16364 -3.26706
H   3.09989 -1.18194 -2.89307
H   1.51436 -1.49841 -2.13339
C   5.94917  1.82105 -0.27282
H   6.13733  2.71780 -0.89276
H   6.29342  2.05656  0.74863
H   6.58735  1.01400 -0.67179
F  -0.20985  0.35304 -1.99664
C  -1.50755 -3.15266 -0.13570
C  -0.39957 -3.33634  0.66505
C  -0.34016 -1.19096 -0.11479
N   0.30192 -2.12161  0.66508
N  -1.45760 -1.83068 -0.59557
C  -0.01125 -4.57297  1.41804
H  -0.58377 -5.43204  1.03475
H   1.05675 -4.81797  1.30916
H  -0.22853 -4.49434  2.49770
C  -2.57573 -4.15314 -0.46147
H  -2.85686 -4.13280 -1.52571
H  -2.21206 -5.16854 -0.23851
H  -3.49473 -3.99801  0.13036
C  -2.46768 -1.17163 -1.48460
H  -2.18042 -0.11322 -1.46789
C   1.56765 -1.84891  1.40958
H   1.73269 -0.77319  1.25146
C   1.43244 -2.10212  2.92130
H   2.29229 -1.63572  3.43000
H   0.51446 -1.65021  3.32983
H   1.44129 -3.17298  3.17978
C   2.76789 -2.58390  0.79101
H   2.85548 -2.35770 -0.28155
H   3.68979 -2.23329  1.28337
H   2.71369 -3.67739  0.92397
C  -3.89743 -1.27370 -0.93135
H  -3.94122 -0.94625  0.11971
H  -4.53786 -0.59143 -1.51457
H  -4.33359 -2.28138 -1.01371
C  -2.31995 -1.65710 -2.93484
H  -3.01496 -1.09041 -3.57704
H  -1.29522 -1.46038 -3.28134
H  -2.54998 -2.72887 -3.05894
```

15

SCF (BP86) Energy = -3296.59193176
Enthalpy 0K = -3295.046265
Enthalpy 298K = -3294.949765
Free Energy 298K = -3295.180007
Lowest Frequency = 8.7611 cm^{-1}
Second Frequency = 11.3484 cm^{-1}
SCF (BP86-D3BJ) Energy = -3297.08873251
SCF (Toluene) Energy = -3296.60061927
SCF (BS2) Energy = -3695.88274151

```
Mg -1.68087 -0.64945  0.34319
Mg  1.68103 -0.64935 -0.34327
```

F	0.00008	-1.49190	-0.00011
O	-1.22162	1.18769	-0.15709
O	1.22178	1.18779	0.15706
N	-3.10062	-2.05136	-0.42063
N	-2.59304	-0.64250	2.25519
N	3.10067	-2.05136	0.42051
N	2.59323	-0.64234	-2.25527
N	0.77367	4.25260	0.76963
N	-0.77387	4.25256	-0.76941
C	-4.62381	-4.00435	-0.13020
H	-5.24625	-3.67363	-0.97712
H	-5.27753	-4.42244	0.64911
H	-3.98433	-4.81774	-0.51229
C	-3.78036	-2.85918	0.42115
C	-3.80331	-2.72748	1.83212
H	-4.37528	-3.50267	2.34741
C	-3.35608	-1.67740	2.66783
C	-3.85151	-1.73007	4.10811
H	-3.03047	-1.56005	4.82306
H	-4.32277	-2.69933	4.32810
H	-4.59489	-0.93561	4.29575
C	-2.50063	0.48034	3.15687
C	-1.41728	0.60040	4.07179
C	-1.36847	1.72063	4.92639
H	-0.53960	1.80833	5.63837
C	-2.35665	2.71119	4.89254
H	-2.30280	3.57146	5.56878
C	-3.42062	2.58434	3.99102
H	-4.19962	3.35504	3.96421
C	-3.52004	1.47870	3.12566
C	-4.70249	1.38948	2.15989
H	-4.68864	0.38294	1.70834
C	-6.06662	1.56898	2.86049
H	-6.20181	0.84590	3.68296
H	-6.88976	1.42048	2.13969
H	-6.18154	2.58209	3.28515
C	-4.52794	2.41067	1.01477
H	-4.52515	3.44283	1.41155
H	-3.57259	2.24032	0.48994
C	-0.31085	-0.44784	4.15634
H	-0.57900	-1.26695	3.46746
C	-0.17114	-1.05253	5.57161
H	0.13011	-0.28856	6.31057
H	0.60520	-1.83682	5.57022
H	-1.11460	-1.50260	5.92563
C	1.04068	0.13478	3.69131
H	0.97613	0.53612	2.66497
H	1.82423	-0.63936	3.71457
H	1.36169	0.95572	4.35849
C	-3.27702	-2.28993	-1.83185
C	-2.53723	-3.30897	-2.50557
C	-2.74609	-3.49332	-3.88739
H	-2.17969	-4.27288	-4.40950
C	-3.66902	-2.72090	-4.60235
H	-3.82514	-2.89438	-5.67245
C	-4.38990	-1.72465	-3.93380
H	-5.11387	-1.11886	-4.49010
C	-4.20359	-1.48283	-2.55881
C	-5.02278	-0.38727	-1.87367
H	-4.57053	-0.21264	-0.88128
C	-6.48546	-0.83439	-1.64413
H	-6.98475	-1.05857	-2.60384
H	-7.06280	-0.03888	-1.14016
H	-6.53940	-1.73934	-1.01685
C	-4.98326	0.94440	-2.65303
H	-3.94447	1.26706	-2.83464
H	-5.49942	1.73623	-2.08246
H	-5.48846	0.86503	-3.63191
C	-1.52783	-4.21243	-1.79057
H	-1.60389	-4.00988	-0.70860
C	-1.80901	-5.71530	-2.02599
H	-2.84715	-5.99524	-1.77849
H	-1.13342	-6.33061	-1.40660
H	-1.63517	-5.99900	-3.07910
C	-0.08117	-3.88016	-2.21755
H	0.05563	-4.01655	-3.30504
H	0.63911	-4.53943	-1.70196
H	0.16740	-2.84162	-1.95591
C	4.62412	-4.00411	0.13010
H	5.24718	-3.67302	0.97643
H	5.27727	-4.42273	-0.64940
H	3.98477	-4.81717	0.51311
C	3.78054	-2.85906	-0.42128
C	3.80358	-2.72728	-1.83224
H	4.37565	-3.50239	-2.34753
C	3.35635	-1.67718	-2.66791
C	3.85189	-1.72974	-4.10816
H	3.03097	-1.55942	-4.82317
H	4.32296	-2.69908	-4.32824
H	4.59549	-0.93544	-4.29560
C	2.50075	0.48050	-3.15695
C	1.41736	0.60050	-4.07183
C	1.36847	1.72072	-4.92644
H	0.53956	1.80836	-5.63840
C	2.35660	2.71132	-4.89264
H	2.30268	3.57158	-5.56889
C	3.42061	2.58454	-3.99115
H	4.19956	3.35528	-3.96438
C	3.52011	1.47891	-3.12578
C	4.70259	1.38978	-2.16003
H	4.68884	0.38321	-1.70853
C	4.52794	2.41090	-1.01487
H	4.52498	3.44308	-1.41162
H	5.35511	2.33258	-0.28647
H	3.57263	2.24041	-0.49000
C	6.06669	1.56945	-2.86063
H	6.20193	0.84645	-3.68316
H	6.88984	1.42097	-2.13985
H	6.18151	2.58260	-3.28522
C	0.31099	-0.44781	-4.15635
H	0.57915	-1.26686	-3.46740
C	-1.04059	0.13478	-3.69142
H	-0.97611	0.53619	-2.66510
H	-1.82410	-0.63940	-3.71467
H	-1.36160	0.95566	-4.35867
C	0.17138	-1.05261	-5.57157
H	-0.12986	-0.28870	-6.31061
H	-0.60493	-1.83693	-5.57018
H	1.11488	-1.50267	-5.92551
C	3.27701	-2.29007	1.83173
C	2.53702	-3.30905	2.50533
C	2.74574	-3.49354	3.88715
H	2.17918	-4.27305	4.40915
C	3.66873	-2.72132	4.60224
H	3.82474	-2.89490	5.67234
C	4.38985	-1.72517	3.93381
H	5.11392	-1.11956	4.49018
C	4.20370	-1.48324	2.55881
C	5.02319	-0.38773	1.87394
H	4.57215	-0.21402	0.88085
C	4.98182	0.94441	2.65243
H	3.94258	1.26678	2.83196
H	5.49871	1.73610	2.08233
H	5.48525	0.86572	3.63227
C	6.48645	-0.83416	1.64671
H	6.98466	-1.05716	2.60726
H	7.06395	-0.03876	1.14276
H	6.54178	-1.73967	1.02035
C	1.52756	-4.21234	1.79018
H	1.60360	-4.00957	0.70825
C	0.08092	-3.88014	2.21727
H	-0.05587	-4.01681	3.30473

```
H  -0.63939 -4.53924  1.70153
H  -0.16762 -2.84152  1.95592
C   1.80870 -5.71527  2.02530
H   2.84680 -5.99522  1.77762
H   1.13302 -6.33043  1.40586
H   1.63498 -5.99914  3.07838
C  -0.00004  3.42464  0.00007
C  -1.70821  3.73775 -1.81424
H  -1.79035  2.66670 -1.55891
C  -3.10837  4.36382 -1.70928
H  -3.46075  4.39073 -0.66662
H  -3.81177  3.73760 -2.28153
H  -3.15783  5.37993 -2.13210
C  -1.09567  3.89057 -3.21450
H  -0.96855  4.94909 -3.50131
H  -1.76880  3.42679 -3.95476
H  -0.11841  3.38851 -3.28606
C  -0.48265  5.59315 -0.49377
C  -1.11644  6.77822 -1.15787
H  -1.10343  6.69752 -2.25688
H  -0.56010  7.69052 -0.89397
H  -2.16467  6.92570 -0.84628
C   0.48225  5.59318  0.49411
C   1.11588  6.77828  1.15831
H   1.10305  6.69740  2.25730
H   0.55929  7.69050  0.89464
H   2.16403  6.92605  0.84659
C   1.70816  3.73784  1.81434
H   1.79041  2.66682  1.55893
C   1.09574  3.89048  3.21467
H   0.96857  4.94896  3.50160
H   1.76897  3.42667  3.95482
H   0.11852  3.38834  3.28627
C   3.10823  4.36410  1.70928
H   3.46055  4.39100  0.66659
H   3.81175  3.73800  2.28152
H   3.15758  5.38023  2.13204
B   0.00005  1.76013  0.00001
H  -5.35508  2.33226  0.28634
```

pinBBu

SCF (BP86) Energy = -569.123611693
Enthalpy 0K = -568.827988
Enthalpy 298K = -568.811084
Free Energy 298K = -568.871244
Lowest Frequency = 17.1407 cm^{-1}
Second Frequency = 53.5275 cm^{-1}
SCF (BP86-D3BJ) Energy = -569.174139930
SCF (Toluene) Energy = -569.125385795
SCF (BS2) Energy = -569.267887458

```
O  -0.86955  1.32355 -0.42098
O  -0.18300 -0.73258  0.38809
C  -2.09600  0.61210 -0.04689
C  -2.54730  1.19499  1.30436
H  -2.67048  2.28517  1.20025
H  -3.50903  0.76697  1.63350
H  -1.79541  1.01090  2.08938
C  -3.15587  0.87903 -1.12021
H  -4.08214  0.31845 -0.90581
H  -3.40478  1.95284 -1.13647
H  -2.79923  0.59839 -2.12243
C  -1.60237 -0.88916  0.05313
C  -2.26924 -1.72283  1.15171
H  -3.35284 -1.82123  0.96660
H  -1.83363 -2.73543  1.16496
H  -2.12193 -1.27719  2.14676
C  -1.65455 -1.63812 -1.29038
H  -1.10344 -2.58741 -1.19226
H  -2.69080 -1.86796 -1.58998
H  -1.17991 -1.05036 -2.09324
B   0.19901  0.53546 -0.02082
C   1.69302  1.04254 -0.01374
H   1.83297  1.66855  0.89301
H   1.83638  1.74484 -0.85814
C   2.76818 -0.06372 -0.04099
H   2.59361 -0.76676  0.79568
H   2.65516 -0.66819 -0.96226
C   4.20767  0.47436  0.03493
H   4.32239  1.07303  0.96015
H   4.37762  1.17930 -0.80264
C   5.27346 -0.63192  0.00174
H   5.20596 -1.22365 -0.92865
H   6.29435 -0.21688  0.05981
H   5.14849 -1.33203  0.84698
```

DMAP

SCF (BP86) Energy = -382.256098610
Enthalpy 0K = -382.098887
Enthalpy 298K = -382.089042
Free Energy 298K = -382.132978
Lowest Frequency = 63.0068 cm^{-1}
Second Frequency = 82.3186 cm^{-1}
SCF (BP86-D3BJ) Energy = -382.285474805
SCF (Toluene) Energy = -382.259778610
SCF (BS2) Energy = -382.353392958

```
N   2.69408  0.00000  0.02022
N  -1.56586 -0.00001 -0.08409
C   1.96745  1.13861  0.00953
H   2.54406  2.07381  0.02487
C   0.57117  1.20572 -0.01449
H   0.08321  2.18308 -0.01244
C  -0.18332  0.00002 -0.03751
C   0.57116 -1.20569 -0.01482
H   0.08318 -2.18303 -0.01314
C   1.96744 -1.13861  0.00919
H   2.54405 -2.07381  0.02429
C  -2.29344  1.25866  0.03158
H  -2.12529  1.76120  1.00588
H  -3.37040  1.06258 -0.07244
H  -2.00183  1.96192 -0.76982
C  -2.29335 -1.25870  0.03216
H  -2.00277 -1.96186 -0.76973
H  -3.37042 -1.06255 -0.07043
H  -2.12398 -1.76139  1.00615
```

Mes_2BF

SCF (BP86) Energy = -823.936666619
Enthalpy 0K = -823.593060
Enthalpy 298K = -823.569587
Free Energy 298K = -823.646947
Lowest Frequency = 19.4346 cm^{-1}
Second Frequency = 31.0036 cm^{-1}
SCF (BP86-D3BJ) Energy = -824.020616453
SCF (Toluene) Energy = -823.938586023
SCF (BS2) Energy = -824.135635883

```
B   0.00002  1.09879  0.00000
F   0.00000  2.46170  0.00008
C   1.41220  0.41202 -0.09012
C   1.64934 -0.68777 -0.96971
C   2.50338  0.90695  0.68798
C   2.93258 -1.25809 -1.04776
C   3.76458  0.29227  0.59783
C   4.00321 -0.79267 -0.26394
H   3.10208 -2.08970 -1.74363
H   4.58782  0.67641  1.21380
C  -1.41217  0.41202  0.09011
C  -1.64945 -0.68739  0.97007
C  -2.50325  0.90669 -0.68837
C  -2.93276 -1.25765  1.04814
```

C -3.76446 0.29213 -0.59816
C -4.00324 -0.79251 0.26403
H -3.10240 -2.08900 1.74427
H -4.58758 0.67610 -1.21439
C -2.34631 2.08556 -1.63435
H -1.47281 1.97469 -2.29991
H -3.23934 2.19619 -2.27064
H -2.20122 3.02803 -1.08102
C -0.55901 -1.25433 1.86365
H -0.08021 -0.47460 2.48055
H -0.97291 -2.01289 2.54813
H   0.24751 -1.72899 1.27933
C -5.36322 -1.45282 0.32916
H -6.17242 -0.73594 0.11136
H -5.44471 -2.27012 -0.41169
H -5.55204 -1.89445 1.32177
C   2.34673 2.08621 1.63352
H   2.20214 3.02855 1.07984
H   1.47309 1.97595 2.29898
H   3.23972 2.19672 2.26992
C   0.55876 -1.25499 -1.86295
H   0.97256 -2.01375 -2.54726
H -0.24763 -1.72953 -1.27835
H   0.07981 -0.47546 -2.47997
C   5.36308 -1.45318 -0.32914
H   5.55606 -1.88571 -1.32499
H   6.17171 -0.73889 -0.10112
H   5.44069 -2.27755 0.40425

NHC-^{i}Pr
SCF (BP86) Energy = -540.685271357
Enthalpy 0K = -540.398003
Enthalpy 298K = -540.381216
Free Energy 298K = -540.440295
Lowest Frequency = 34.2969 cm^{-1}
Second Frequency = 46.8042 cm^{-1}
SCF (BP86-D3BJ) Energy = -540.739499622
SCF (Toluene) Energy = -540.688639734
SCF (BS2) Energy = -540.818289076

C -0.00001 -1.26967 -0.16946
C -0.69041 0.94313 0.06082
C   0.69044 0.94312 0.06077
C   1.61990 2.11626 0.15565
H   2.38598 1.98724 0.93975
H   2.15146 2.31474 -0.79290
H   1.05307 3.02817 0.40442
C -1.61987 2.11626 0.15572
H -2.38604 1.98712 0.93972
H -1.05307 3.02813 0.40472
H -2.15131 2.31488 -0.79287
N -1.07390 -0.40490 -0.08263
N   1.07390 -0.40491 -0.08272
C -2.43931 -0.97184 -0.10551
H -2.23473 -2.03896 -0.30039
C   2.43929 -0.97187 -0.10551
H   2.23474 -2.03897 -0.30047
C -3.29616 -0.42608 -1.26063
H -4.22094 -1.02152 -1.34878
H -3.59521 0.62359 -1.10390
H -2.75122 -0.49531 -2.21623
C -3.13702 -0.86431 1.26223
H -4.07901 -1.43911 1.24931
H -2.49156 -1.27540 2.05512
H -3.38936 0.17733 1.52425
C   3.13685 -0.86438 1.26231
H   2.49133 -1.27563 2.05508
H   4.07892 -1.43905 1.24946
H   3.38898 0.17728 1.52444
C   3.29629 -0.42606 -1.26052
H   4.22101 -1.02160 -1.34864
H   2.75143 -0.49514 -2.21617
H   3.59545 0.62356 -1.10365

^{3}Bpin^{-}
SCF (BP86) Energy = -411.088791767
Enthalpy 0K = -410.919961
Enthalpy 298K = -410.908860
Free Energy 298K = -410.954798
Lowest Frequency = 66.1874 cm^{-1}
Second Frequency = 199.3864 cm^{-1}
SCF (BP86-D3BJ) Energy = -411.121979103
SCF (Toluene) Energy = -411.137444083
SCF (BS2) Energy = -411.249556127

O   1.08533 1.31149 0.13649
B -0.15396 1.99969 -0.32332
O -1.07713 0.98224 -0.84269
C   0.80339 -0.08527 0.07798
C   1.46256 -0.64314 -1.20786
H   2.52870 -0.35337 -1.20965
H   1.39577 -1.74734 -1.29781
H   0.97575 -0.18441 -2.08448
C   1.41629 -0.76646 1.31657
H   1.18234 -1.84780 1.35926
H   2.51692 -0.65584 1.29225
H   1.05018 -0.28808 2.23932
C -0.78866 -0.17188 -0.01875
C -1.32972 -1.42128 -0.72956
H -1.01988 -2.35278 -0.21943
H -2.43740 -1.39381 -0.73172
H -0.98595 -1.45719 -1.78047
C -1.48335 0.02370 1.34716
H -2.57944 -0.08278 1.21464
H -1.16218 -0.68259 2.13369
H -1.24370 1.08375 1.63744

$^{3}BO_2^{-}$
SCF (BP86) Energy = -175.265749117
Enthalpy 0K = -175.259274
Enthalpy 298K = -175.255213
Free Energy 298K = -175.283435
Lowest Frequency = 467.9934 cm^{-1}
Second Frequency = 1183.6882 cm^{-1}
SCF (BP86-D3BJ) Energy = -175.267358394
SCF (Toluene) Energy = -175.325283605
SCF (BS2) Energy = -175.365631893

B   0.00000 0.00000 0.54612
O   0.00000 1.14940 -0.17066
O   0.00000 -1.14940 -0.17066

References

[1] A. P. Dove, V. C. Gibson, P. Hormnirun, E. L. Marshall, J. A. Segal, A. J. P. White, D. J. Williams, D. J. *Dalton. Trans.* **2003**, 3088.

[2] A. F. Pécharman, A. Colebatch, M. S. Hill, C. L. McMullin, M. F. Mahon, C. Weetman, *Nature Commun.* **2017**, *8*, 15022.

[3] N. Kuhn, T. Kratz, *Synthesis-Stuttgart* **1993**, 561-562.

[4] H. Asakawa, K. H. Lee, Z. Y. Lin, M. Yamashita, *Nature Commun.* **2014**, *5*, 4245.

[5] L. J. Bourhis, O. V. Dolomanov, R. J. Gildea, J. A. K. Howard, H. Puschmann, H. *Acta Cryst. A* **2015** *71*, 59-75.

[6] G. M. Sheldrick, *Acta Cryst. C* **2015**, *71*, 3-8.

[7] D. Kratzert, J. J. Holstein, I. Krossing, *J. Appl. Cryst*. **2015**, *48*, 933-938.

[8] Frisch, M. J.; Trucks, G. W.; Schlegel, H. B.; Scuseria, G. E.; Robb, M. A.; Cheeseman, J. R.; Scalmani, G.; Barone, V.; Mennucci, B.; Petersson, G. A.; Nakatsuji, H.; Caricato, M.; Li, X.; Hratchian, H. P.; Izmaylov, A. F.; Bloino, J.; Zheng, G.; Sonnenberg, J. L.; Hada, M.; Ehara, M.; Toyota, K.; Fukuda, R.; Hasegawa, J.; Ishida, M.; Nakajima, T.; Honda, Y.; Kitao, O.; Nakai, H.; Vreven, T.; Montgomery, J. A., Jr.; Peralta, J. E.; Ogliaro, F.; Bearpark, M.; Heyd, J. J.; Brothers, E.; Kudin, K. N.; Staroverov, V. N.; Kobayashi, R.; Normand, J.; Raghavachari, K.; Rendell, A.; Burant, J. C.; Iyengar, S. S.; Tomasi, J.; Cossi, M.; Rega, N.; Millam, J. M.; Klene, M.; Knox, J. E.; Cross, J. B.; Bakken, V.; Adamo, C.; Jaramillo, J.; Gomperts, R.; Stratmann, R. E.; Yazyev, O.; Austin, A. J.; Cammi, R.; Pomelli, C.; Ochterski, J. W.; Martin, R. L.; Morokuma, K.; Zakrzewski, V. G.; Voth, G. A.; Salvador, P.; Dannenberg, J. J.; Dapprich, S.; Daniels, A. D.; Farkas, O.; Foresman, J. B.; Ortiz, J. V.; Cioslowski, J.; Fox, D. J. Gaussian 09 (Revision D.01); Gaussian Inc.: Wallingford, CT, 2009.

[9] D. Andrae, U. Häußermann, M. Dolg, H. Stoll, H. Preuß, *Theor. Chim. Acta* **1990**, *77*, 123−141.

[10] (a) P. C. Hariharan, J. A. Pople, *Theor. Chim. Acta* **1973**, *28*, 213−222. (b) W. J. Hehre, R. Ditchfield, J. A. Pople, *J. Chem. Phys.* **1972**, *56*, 2257.

[11] (a) A. D. Becke, *Phys. Rev. A: At., Mol., Opt. Phys.* **1988**, *38*, 3098. (b) J. P. Perdew, *Phys. Rev. B: Condens. Matter Mater. Phys.* **1986**, *33*, 8822−8824.

[12] J. Tomasi, B. Mennucci, R. Cammi, *Chem. Rev.* **2005**, *105*, 2999−3094.

[13] S. Grimme, S. Ehrlich, L. Goerigk, *J. Comp. Chem.* **2011**, *32*, 1456-1465.
